# Understanding reaction to corporate activism: The moderating role of polarization

**DOI:** 10.1093/pnasnexus/pgae313

**Published:** 2024-10-15

**Authors:** Luiza Braga, Amir Grinstein, Matheus Tardin, Marcelo Perin

**Affiliations:** FGV EAESP, Department of Marketing, Sao Paulo, 01313-902, Brazil; Northeastern University, Department of Marketing, Boston, 02115, USA; Vrije Universiteit Amsterdam, Department of Marketing, Amsterdam, 1081 HV, The Netherlands; FGV EAESP, Department of Marketing, Sao Paulo, 01313-902, Brazil; FGV EAESP, Department of Marketing, Sao Paulo, 01313-902, Brazil

**Keywords:** corporate activism, polarization, political orientation, meta-analysis

## Abstract

In our polarized societies, more companies are taking a stand on divisive sociopolitical issues. However, given the mixed findings from previous studies, it remains unclear whether Corporate Activism (CA) is more likely to hurt or help a company's performance and reputation, or shape the public's attitudes toward the sociopolitical issue involved. To better understand the impact of CA in polarized societies, it is valuable to study moderating factors, especially those linked to polarization. A meta-analysis of 72 scholarly works is conducted to examine the impact of CA on various outcomes (e.g. ads and social media engagement, cognitive and attitudinal reactions, public's intentions and actions, emotional reactions, social and ethical engagement, workplace, and employee perceptions) and the role of moderators (a sociopolitical issue's political leaning and controversy level, political orientation of the target audience, key demographics). The analysis reveals a positive, albeit small, effect size (0.085 [95% CI (0.0542, 0.1158)]) with the most impact on two outcomes: cognitive and attitudinal reactions, and emotional reactions. It further reveals that companies adopting liberal-leaning CAs elicit more favorable responses than those adopting conservative CAs and that liberals respond positively to CA, while conservatives are more neutral. However, when there is alignment between the CA's political leaning and the audience's political orientation, conservatives have a stronger positive response than liberals. Also, younger audiences view CA more positively. Finally, per national culture, while power distance and individualism positively moderate the reaction to CA, uncertainty avoidance has a negative effect.

**Significance Statement**

This research applies meta-analysis to examine Corporate Activism (CA)—the growing trend of companies taking a stand on sociopolitical divisive issues—and its outcomes amid polarized societies. It reveals that CA elicits a small positive response, and that this effect is influenced by moderators linked to polarization. These moderators include CA's political leaning and controversy level, the political orientation of the target audience, and the interaction of these factors. The research also emphasizes the impact of demographic and cultural dimensions, such as age, individualism, power distance, and uncertainty avoidance. Overall, this research consolidates existing and mixed empirical findings on CA in today's polarized environment and proposes directions for future research.

## Introduction

Our societies are becoming increasingly polarized.^1^ In this context, there is a growing trend involving the expectation that companies—typically following pressure from stakeholders such as the general public, customers, and employees—take a public stand on divisive, controversial sociopolitical issues (2). We label this phenomenon Corporate Activism (CA). CA can be manifested in various forms: through marketing communication, executives speaking up on sociopolitical issues, sponsorship of events or organizations dedicated to social causes, or changes in corporate policies, products, or practices that demonstrate a commitment to sociopolitical causes (3). This trend reflects the evolving role of companies in the political and social discourse. Some companies adopt CA proactively, incorporating it as a core aspect of their business, as a differentiation strategy, and/or as an expression of their corporate values (4). Other companies engage in CA reactively (5, 6), often to mitigate negative publicity or address stakeholder demands. Furthermore, as research on collective action and its interaction with the branding literature suggests, companies can leverage collective action to engage with emerging sociopolitical issues and collaborate with various stakeholders and social movement organizations (7–9).

Although CA shares similarities with related concepts such as Corporate Social Responsibility (CSR), which also links companies to sociopolitical issues, it differs in its intent and the issues it addresses. The primary goal of CSR is to allow companies to be socially responsible by balancing economic performance with social and environmental well-being. Conversely, CA's primary goals include a focus on influencing public policy or moral standards and to explicitly clarify a company's position on specific sociopolitical issues (2, 10). Moreover, CSR typically focuses on broadly supported social and environmental initiatives, whereas CA involves taking stances on divisive sociopolitical issues (2, 11).

Companies have adopted stances that align across the liberal-conservative spectrum, reflecting their diverse political orientations (5, 12). Recent examples of companies adopting a liberal-leaning CA include Nike's campaign featuring Colin Kaepernick, which was associated with the fight against racism and police brutality (13, 14), Bud Light's use of a transgender influencer to become more inclusive (15), Disney's opposition to Florida's “Don’t Say Gay” bill (16), and Ben & Jerry's support for a range of causes, including climate change, refugee rights, and racial justice (17). Conservative-leaning CA cases include Chick-fil-A's donations to organizations that oppose same-sex marriage (18), Spotify's support for freedom of speech in the case of Joe Rogan (19), and Goya Foods CEO's public support for President Trump (20).

One useful theoretical lens to guide the study of CA and its impact is signaling theory (21, 22). This theory sheds light on the signal companies are sending to highlight their values and moral position (CA), the signaler (the company), the recipient of the signal (the target audience for the signal, i.e. various stakeholders), the signaling environment (the environment in which the different actors operate), and the feedback to the signal (how various stakeholders respond to the signal). According to signaling theory, information asymmetry gaps exist between companies and their stakeholders (23). When a company takes a stand on polarized sociopolitical issues by applying CA, it addresses the informational asymmetry gap. CA serves as a signal that discloses a company's identity as an active societal participant, as well as its morals and values. This behavior is also aimed at garnering favorable evaluations from both internal and external audiences (2).

Further, we highlight the role of lifestyle politics theory and social identity theory (SIT) to better understand how different target audiences react to the signal (CA) and the conditions under which CA will likely be effective in a polarized society. Lifestyle politics theory suggests that factors such as globalization and digitalization have heightened individual self-reflexivity and political expression (24–26), making consumers more likely to engage with companies that mirror their political and social values. This is particularly true for individuals with strong political convictions, who may engage in political consumerism—the intentional purchase or avoidance of products or services based on political motives. These individuals are likely to support companies aligned with their ideologies (24) and oppose those that represent contrary values (26).

SIT adopts a collective view to explain reactions to CA. A social identity is characterized as a component of an individual's self-concept derived from their awareness of belonging to one or more social groups, along with the associated values and emotional importance of such memberships (27). According to this viewpoint, any behaviors, either by the individual or by members of their group, that conflict with this identity can pose a threat to one's self-concept (28). SIT offers insights into how individuals endorse a company's identity, building their self-identity through their affiliations with various groups, including their workplace or brands (29). This theory highlights how connections with different social entities shape self-perception. Specifically, research has demonstrated how individuals embrace a company's identity as part of their own identity (30). Furthermore, social identity impacts behavior through social norms, which are the shared beliefs within a group about how members should act. These norms create expectations within the group and motivate conformity, sometimes at personal costs, to align with these shared beliefs (28). Along with the SIT framework, one particular concept that is relevant to our discussion is affective polarization (31).^2^ It extends the idea of polarization beyond simple policy disagreements to manifest as profound emotional divisions between groups. This division, in turn, can lead individuals to view those with opposing viewpoints with disdain or hostility (32). This emotional divide highlights how political considerations extend into nonpolitical spheres, affecting how individuals interact with others, including corporate entities (33).

But what do we currently know about how the public in polarized environments responds to CA? Emerging research reveals mixed findings, with both positive and negative effects on corporate reputation and image (17, 34), intentions to boycott as well as buycott (35, 36), and different attitudes toward sociopolitical issues (37, 38). Thus, it is still unclear how CA influences business and marketing outcomes such as performance and brand reputation, as well as public attitudes toward sociopolitical issues.

The above highlights the need to uncover the moderating conditions under which CA can lead to more positive company and societal outcomes in polarized societies. Potential moderators are guided by signaling theory—capturing aspects of the signal, the signaler, recipients of the signal, and the signaling environment—ultimately evaluating the reaction to the signal. Also, the moderators include individual-level variables and how they interact with group-level variables, inspired by lifestyle politics theory and SIT. Specifically, per substantive moderators, the current research focuses on two groups: (i) CA characteristics, including CA political leaning and sociopolitical issue polarization level; (ii) target audience characteristics, including political orientation, age, gender, income, education, racial diversity, and country of residence and the associated national culture. The current work also studies methodological moderators related to characteristics of the research work, including whether the work has been published or not, publication quality, year of publication, and the method used. Finally, per the outcomes studied—i.e. reaction to CA—this research focuses on multiple outcomes, including ads and social media engagement (e.g. ad attitude, click-through rates), cognitive and attitudinal reactions (e.g. brand awareness, loyalty), public's intentions and actions (e.g. boycott, willingness to pay), emotional reactions (e.g. brand love, empathy), social and ethical engagement (e.g. issue advocacy, donation), and workplace and employee perceptions (e.g. employer attractiveness, quality of work).

Per CA characteristics, as evident, some of the CA efforts lean liberal and others lean conservative; still, whether CA effectiveness depends on the political leaning of CA is unclear. Also, the target audience that is exposed to CA, and especially its political orientation, is an important factor in understanding responses to CA (10, 39). Prior empirical evidence shows that stakeholders with political views that align with the company's stance are more likely to respond positively. For example, previous work has found such alignment to lead stakeholders to see the company's actions as affirmations of their values (e.g. (10)), and increase purchase intention (25), positive attitudes toward the company (26) or the sociopolitical issue (27). Conversely, those whose political orientations diverge from the company's stance may view CA initiatives with skepticism (12) or outright opposition (14, 35, 36). This interplay suggests that aligning a company's CA with the political orientation of its stakeholders, especially the CA's target audience, is valuable.

Prior work jointly studying the political leaning of CA and the political orientation of the target audience has shown nuanced empirical evidence. Some research indicates that conservative CA initiatives yield favorable outcomes in the eyes of conservatives when it is a buycotting campaign, whereas it can yield favorable outcomes in the eyes of liberals pursuing a boycotting campaign (e.g. (20)). Moreover, distinct patterns can emerge based on political orientations, where liberals tend to react positively to CA that resonates with liberal viewpoints, whereas conservatives, on average, exhibit a more neutral response to conservative-leaning CA (e.g. (40, 41)). Based on lifestyle politics theory, we argue that liberal audiences tend to respond positively to CA as it often aligns with their lifestyle politics that prioritize progressive change and social issues. For conservatives, the impact of CA may be less significant because their lifestyle politics prioritize different, often more traditional values that are less frequently addressed by typical CA initiatives.

Beyond CA's political leaning and the political orientation of the target audience, it is valuable to acknowledge that different sociopolitical issues may have different controversial levels (e.g. abortion vs. climate change) (4, 42, 43). By controversial, we mean issues that elicit widespread deep disagreement and strongly divided opinions among the public. Although alienation risk is intrinsic to CA (44), this risk is expected to increase the more controversial the sociopolitical issue is (10, 12, 45).

Demographic characteristics may also play an important role in shaping response to CA. According to SIT, people's identification with social groups based on race, gender, ethnicity, and other demographic factors significantly influences their attitudes and behaviors (46) and determines their engagement in political participation and political consumerism (24). For instance, the younger generation, often characterized by a heightened awareness of social justice and environmental concerns, is likely to respond more positively to CA (47). Similarly, individuals with higher income and education levels, which are often associated with greater access to information and a broader understanding of sociopolitical issues (48), may also respond more favorably to CA. Racial diversity, including individuals from marginalized communities, may also show more positive engagement with CA efforts, given their motivation to improve livelihoods and create social change (49).

The country of residence can also play a significant role, with varying reactions to CA potentially emerging based on national and cultural characteristics. For instance, in countries with high political polarization, the response to CA might differ from countries with a more consensus-driven political culture (50). Moreover, in countries where citizens are more involved in politics (51), CA might be subjected to greater scrutiny and debate. Further, national cultural differences (52) can also influence how CA is received. In cultures with elevated levels of uncertainty avoidance, for instance, CA initiatives—that are perceived as riskier (27)—might be met with resistance. In contrast, cultures that score high on individualism might see CA as an affirmation of personal freedoms and rights, enhancing the potential for positive engagement.

**Fig. 1.**
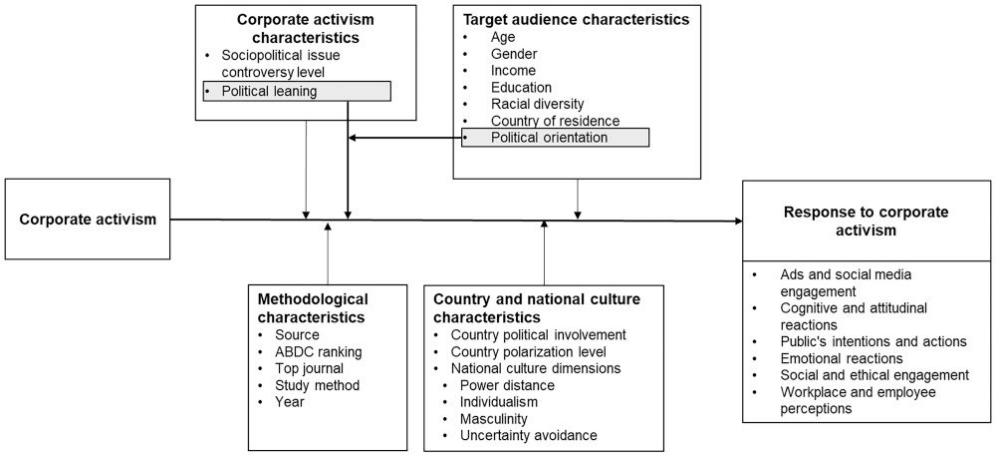
Conceptual framework.

The conceptual framework that summarizes the studied variables and proposed linkages is outlined in Fig. 1.

## Analysis and results

The current research employed a meta-analysis, a statistical approach that aggregates findings from prior research to identify the association between variables of interest. Additionally, it permits the comparison of research outcomes across various contextual and methodological factors, thereby identifying areas of inconsistency that require further exploration. Furthermore, it enables empirical generalizations, which in turn facilitate theory improvement and inspire the conduct of additional empirical work (38, 39). Such analysis can help explain the heterogeneity in CA effectiveness, particularly when considering moderators that earlier independent studies could not have addressed or that demonstrated mixed findings about their impact (40).

The meta-analysis was based on data from 72 scholarly works in economics, management, marketing, public relations, political science, psychology, and cognitive sciences. The sample includes articles published in peer-reviewed journals (*n* = 52), working papers (*n* = 3), conference proceedings (*n* = 3), doctoral dissertations (*n* = 5), and master theses (*n* = 9). A total of 448 effect sizes (ES) were collected from the 72 works analyzed, derived from *N* = 4,432,309 individual observations. The details of the meta-analytical data and procedure are provided in the Materials and Methods section. See SI Appendix A for further information on the coding and data.

### Univariate analysis

The overall meta-analytical result, the correlation between CA and response to CA (an aggregate measure of all outcome categories), was calculated using Pearson's correlation coefficient. This revealed a positive, albeit small, ES of 0.085 (95% CI [0.0542, 0.1158]). In terms of common language effect size (53), this indicates a 54.3% probability that a randomly selected individual exposed to CA will have a more favorable response than a randomly selected individual not exposed to CA. This indicates a modest, yet statistically significant, positive impact of CA. Further, the analysis suggests considerable heterogeneity across studies, as demonstrated by an *I*² of 98.80%. Univariate results are presented in SI Appendix B.

### Hierarchical meta-regressions

**Fig. 2.**
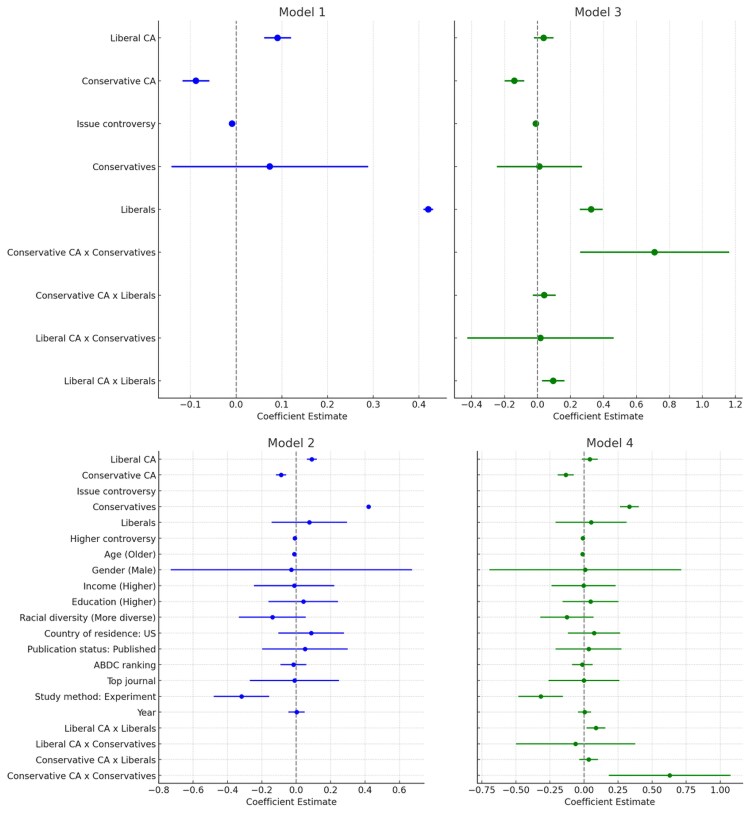
Coefficient plots.

Although univariate analysis offers valuable insights into individual variable effects, it falls short by not accounting for the interactions among multiple variables. Consequently, we shift our focus to multivariate analysis, which allows us to examine various factors concurrently, thereby capturing the dynamics and mutual dependencies influencing CA's outcomes. In this context, we conducted a series of meta-regressions. To account for variability, we incorporated random-effects at the study level to capture variations in outcome measures across different studies. Findings are reported in Table 1 and illustrated in Fig. 2.

**Table 1.** Hierarchical meta-regressions on response to CA.

Variables/Model	Model 1	Model 2	Model 3	Model 4
**Main variables**				
*CA political leaning*				
Liberal	0.0906^c^	0.0904^c^	0.0373	0.0430
	(0.0149)	(0.0149)	(0.0302)	(0.0302)
Conservative	−0.0880^c^	−0.0882^c^	−0.1401^c^	−0.1344^c^
	(0.0150)	(0.0150)	(0.0302)	(0.0303)
*Target audience political orientation*				
Liberals	0.4205^c^	0.4208^c^	0.3253^c^	0.3329^c^
	(0.0054)	(0.0054)	(0.0354)	(0.0355)
Conservatives	0.0736	0.0763	0.0115	0.0529
	(0.1098)	(0.1120)	(0.1316)	(0.1331)
*Sociopolitical issue controversy level*	−0.0089^c^	−0.0089^c^	−0.0101^c^	−0.0102^c^
	(0.0021)	(0.0021)	(0.0022)	(0.0022)
*Interactions*				
Liberal CA × Liberals			0.0954^b^	0.088^a^
			(0.0350)	(0.0351)
Liberal CA × Conservatives			0.0182	−0.062
			(0.2261)	(0.2235)
Conservative CA × Liberals			0.0407	0.0336
			(0.0351)	(0.0352)
Conservative CA × Conservatives			0.7097^b^	0.6296^b^
			(0.2304)	(0.2277)
*Target audience demographics*				
Age		−0.0120^a^		−0.0118^a^
		(0.0057)		(0.0058)
Gender (Male)		−0.0284		0.0094
		(0.3580)		(0.3597)
Income		−0.0123		−0.0031
		(0.1194)		(0.12)
Education		0.0410		0.0481
		(0.1035)		(0.1053)
Racial diversity (More diverse)		−0.1387		−0.1258
		(0.0995)		(0.1000)
Country of residence: US (vs. notvUS)		0.0866		0.0735
		(0.0975)		(0.0979)
**Methodological characteristics**				
Publication status: Published (vs. Unpublished)		0.0505		0.0336
		(0.1270)		(0.1232)
ABDC ranking		−0.0160		−0.0127
		(0.0384)		(0.0388)
Top journal: Yes (vs. No)		−0.0104		−0.0012
		(0.1320)		(0.1326)
Study method: Experiment (vs. No)		−0.3182^c^		−0.3182^c^
		(0.0824)		(0.0831)
Year		0.0020		0.0043
		(0.0243)		(0.0248)
Intercept	−0.7201^c^	−4.5602	−0.7619^c^	−1.8532
	(0.1583)	(4.9146)	(0.1599)	(4.7886)
*k*	448	448	448	448

Variables definitions are available in Appendix A Table S2. The values in parentheses are the standard errors.

^a^ *P* < 0.05.

^b^ *P* < 0.01.

^c^ *P* < 0.001.

Our basic model (model 1 including only CA political leaning, sociopolitical issue controversy level, and target audience's political orientation) illustrates that companies taking a liberal CA stand, as opposed to a conservative one, show a notable positive effect. Specifically, conservative-leaning CAs have a negative moderating effect (*B* = −0.0880, SE = 0.0150, *P* < 0.001), while liberal-leaning CAs show a positive impact (*B* = 0.0906, SE = 0.0149, *P* < 0.001). With the target audience, liberals exhibit a stronger positive response to CA (*B* = 0.4205, SE = 0.0054, *P* < 0.001) compared to conservatives (*B* = 0.0736, SE = 0.1098, *P* < 0.010). Further, the controversy level of the sociopolitical issue supported shows an inverse relationship with response to CA (*B* = −0.0089, SE = 0.0021, *P* < 0.001).

Our extended model (model 2, which also includes target audience demographics and methodological characteristics) shows that the main results from model 1 are robust. Results also reveal age as a significant factor, with younger individuals responding more positively to CA (*B* = 0.0120, SE = 0.0057, *P* < 0.01). The influence of other demographic characteristics is not significant. This is also the case with most methodological characteristics, except study method. Specifically, experiments—in contrast with observational studies—present a negative effect on outcomes (*B* = −0.3182, SE = 0.0824, *P* < 0.05).

In model 3, we introduce interaction terms to explore the joint effect of CA political leaning and the target audience's political orientation on response to CA. Liberal-leaning CA interacting with liberals shows a significant positive effect (*B* = 0.0954, SE = 0.0350, *P* < 0.01). When conservative-leaning CA interacts with conservatives, we observe a stronger positive effect (*B* = 0.7097, SE = 0.2304, *P* < 0.01). For the interaction between conservative-leaning CA and liberals (*B* = 0.0407, SE = 0.0351, *P* > 0.05) and the interaction between liberal-leaning CA and conservatives (*B* = 0.0182, SE = 0.2261, *P* > 0.05), the results show nonsignificant effects. Interaction results are robust when including demographic and methodological characteristics (model 4).

### Additional analysis

We performed two additional analyses: one for country and national culture effects and one for the six categories of the dependent variable. In the context of the country and national culture effects (SI Appendix C, Table S6), studies in our sample were conducted in 16 different countries; however, most of them (338 ES, 77%) are from the United States. Nonetheless, to explore country-level effects, three distinct factors were tested: Hofstede's cultural dimensions (power distance, individualism, masculinity, and uncertainty avoidance) (52), the World Values Survey's level of political involvement (51), and Edelman's country polarization level (50). When our main variables (CA political leaning, sociopolitical issue controversy, and target audience's political orientation) were included in the meta-regressions, only the effects associated with Hofstede's dimensions of power distance (*B* = 0.0220, SE = 0.0052, *P* = 0.0017), individualism (*B* = 0.0113, SE = 0.0052, *P* = 0.0294), and uncertainty avoidance (*B* = −0.0073, SE = 0.0033, *P* = 0.0252) remained significant^3^.

In the context of the six outcome categories (SI Appendix C, Table S8), results should be viewed cautiously, given that some categories include a small number of ES. Our meta-regression analysis reveals that CA significantly impacts cognitive and attitudinal reactions (*B* = 0.1682, SE = 0.0412, *P* < 0.0001) and emotional reactions (*B* = 0.1337, SE = 0.0614, *P* = 0.0295). The effects of ads and social media engagement (*B* = −0.0419, SE = 0.1023), public's intentions and actions (*B* = 0.073, SE = 0.0395), social and ethical engagement (*B* = 0.1724, SE = 0.0937), and workplace and employee perceptions (*B* = 0.0262, SE = 0.0892) are directionally positive but not statistically significant.

### Sensitivity analysis

To test the robustness of our effects, we performed multiple sensitivity analyses. First, we addressed the issue of outliers. We ran the overall (univariate) analysis without three outliers (20, 39, 54), with *N* = 2,779,338 households, *N* = 1,563,885 mobile phone locations, and *N* = 25,514 Facebook users, respectively. Apart from those samples, all other studies collected survey data (*N* = 63,572 individuals), leaving an effective *k* = 438. The analysis did not change the nature of our results (ES of 0.088 [95% CI (0.0568; 0.1196)]). Further, when analyzing the entire sample, we found no correlation between sample size and ES (*r* = −0.034, *P* = 0.457), indicating that outliers are not a concern in our data. Finally, our analysis uses the “metacor” package (55). This package indirectly accounts for sample size by using the inverse of the variance of ES as weights in the model. Since the variance typically decreases with larger sample sizes, this method naturally integrates sample size considerations into the estimation of the overall ES (56).

Next, we focused on alternative coding of our main variables. First, we tested the target audience's political orientation as a continuous variable, where higher values indicate a more conservative sample (SI Appendix D, Table S9). We find consistent evidence that the more conservative the sample, the more negative the response to CA (*B* = −2.9515, SE = 0.1848, *P* < 0.001). Similarly, the interaction with a liberal CA yields a negative response (*B* = −1.1639, SE = 0.1015, *P* < 0.001), while interaction with a conservative CA yields a positive response (*B* = 1.4849, SE = 0.1047, *P* < 0.001). We further tested for a direct contrast of liberals and conservatives for both CA and the target audience's political orientation by including an “unknown” category. “Unknown” codes were given to observations when data for a moderator variable was unavailable due to limited information in the original studies (e.g. (57)), (SI Appendix D, Table S10). Again, results are consistent, such that the effect for liberal CAs is positive when compared with conservative CAs (*B* = 0.1786, SE = 0.0053, *P* < 0.001) and positive for liberals when compared with conservatives (*B* = 0.3469, SE = 0.1098, *P* < 0.01). Finally, we tested the alignment between CA political leaning and the target audience's political orientation using a different measure of alignment/misalignment. Specifically, when the study considered whether the audience agrees or disagrees with the CA or whether their values are congruent with the CA's values (e.g. (58, 59)), (SI Appendix D, Table S11). Results show a positive effect for aligned audiences (*B* = 0.0141, SE = 0.0006, *P* < 0.001) and a negative effect for misaligned audiences (*B* = −0.0049, SE = 0.0006, *P* < 0.001).

Finally, given the meaningful number of nonpublished research work in our sample (20 out of 72) we also analyzed the data only with published research. This analysis did not alter the nature of the original results (see univariate results in SI Appendix B).

## Discussion

The current meta-analysis on CA and its outcomes shows that this relationship is influenced by multiple moderators linked to polarization. Findings on the political leaning of CA initiatives reveal that liberal-leaning CAs generally receive more favorable reactions than those with a conservative-leaning. One explanation can be that the majority of CA efforts tend to be liberal-leaning (2, 5), which may foster a social norm or pressure to support such behavior, as it is perceived to be more common or aligned with societal expectations. A second explanation may be methodological, given that some studies utilize relatively young respondents (the average age in our sample is 34.7 years), and younger people, on average, are more liberal (60, 61).

When considering the target audience's political orientation, liberals react more positively than conservatives to CA. This can be explained by the notion that liberals are more likely to engage in political participation as a form of protest to challenge the status quo, driven by lifestyle politics that aligns with progressive ideologies (24, 62). This predisposition may make them more responsive to CA, viewing it as an extension of their political and social engagement. Conversely, conservatives may exhibit a weaker reaction to CA, possibly due to a preference for traditional forms of political participation and a perception of consumer choices as separate from their political expression.

Another set of findings refers to the importance of alignment between CA and its target audience. Specifically, individuals respond more positively to CA when there is a fit between the CA's political leaning and the target audience's political orientation. Dual-process theory can explain this (63, 64). Aligned audiences engage in central (systematic) processing, dedicating more cognitive resources to understand and evaluate the CA initiative. This deep processing, motivated by the congruence between the audience's values and the company's activism, leads to a more favorable evaluation and stronger support for the company and its actions.

Interestingly, conservatives respond much more positively to CA efforts aligned with their values than liberals responding to liberal-leaning CAs. The scarcity of conservative-leaning CAs in the current sociopolitical landscape coupled with the contrast effect (65, 66) could offer a possible explanation. Given that CA is more frequently associated with liberal causes, conservatives might perceive aligned CA efforts as more distinctive, noteworthy, and valuable. A heightened sense of community and solidarity may emerge when conservatives’ values are publicly supported, unlike liberals who may view liberal-leaning CA as expected or normative, thus eliciting a less intensified reaction.

Finally, in this context of (mis)alignment, the nonsignificant interactions involving politically misaligned audiences could also be explained by dual-process theory (63, 64), suggesting that these audiences may resort to peripheral (heuristic) processing. Lacking motivation to deeply engage with the CA content that contradicts their political orientation, misaligned audiences are likely to rely on general perceptions of the company or external opinions rather than a detailed critique of the CA initiative. This peripheral processing may result in a neutral or minimal impact on the audience's behavior and attitudes toward the company, as their political misalignment does not trigger a significant negative response. Conversely, other factors, such as convenience or product satisfaction, may play a more central role in their decision-making.

CA impacts different outcome variables differently. Cognitive and attitudinal reactions, and emotional reactions are outcome categories where CA has a more positive impact. CA appears to have less effect on several forms of engagement, such as ads and social media activity, social and ethical engagement, as well as workplace and employee perceptions. This could imply that while CA effectively influences mindsets and emotions, these effects do not directly or strongly translate into actions, either in the digital space, the work environment, or with the cause itself.

Another finding is that CA of sociopolitical issues with low controversy level receives more positive responses. Such issues typically enjoy broader consensus, reducing the risk of alienating the target audience. Notably, even this CA effort has not produced a strong positive effect. A possible reason is that noncontroversial CA might be viewed as more similar to CSR and, therefore less distinctive, making it more in line with the public's expectations (10, 67).

Findings also suggest that only some of the target audience demographic characteristics—gender, income, education, racial diversity, country of residence, and the associated national culture—showed consistent effects across the univariate and multivariate analyses. First, younger audiences are more likely to have positive responses to CA. This finding potentially reflects a generational shift toward a more pronounced expectation of corporate responsibility and active engagement in sociopolitical issues (68). The positive response from younger demographics might also stem from a broader inclination toward social engagement, potentially seen as fashionable or normative behavior within their peer groups (47). Second, we find that three national culture dimensions moderate the impact of CA, namely power distance, individualism, and uncertainty avoidance. The influence of national culture on the effectiveness of CA highlights the interplay between a company's sociopolitical stance and the cultural context in which it operates. Thus, companies may highlight, for instance, in more individualist cultures, the way CA is linked to one's freedom of choice and the ability to make a (social) change, or in cultures high on power distance, emphasize engagement with CA as a social status signal. Conversely, in high uncertainty avoidance cultures, companies may decide not to launch CA efforts or motivate engagement with CA by highlighting the benefits over the risks.

Per the methodological moderators, findings indicate that the response to CA is not significantly different across journal quality or publication status (although, still, better quality outlets seem to demonstrate directionally better outcomes for CA). Results show that the study method is a relevant moderator. Specifically, studies applying experiments tend to report smaller effects than those using other methods, such as surveys and big data. This finding might be attributed to the precise control over variables and the artificial settings of experimental studies (69). It can also be linked to the notion that, in reality, firms and managers are more likely to be risk-averse in their CA, whereas scholars who design experiments can engage in hypothetical CA without such risk considerations.

### Contributions

This research presents valuable theoretical and practical contributions to understanding CA in polarized societies. It systematically consolidates prior empirical research and addresses previous inconsistencies, providing a robust framework to understand the dynamics of CA and its outcomes. Theoretically, it develops the literature by examining the role of political orientation and polarization in shaping response to CA. The findings highlight the significant role of CA characteristics such as political leaning and the level of sociopolitical issue controversy. Moreover, through the lens of lifestyle politics theory and SIT, we allow for a better understanding of the willingness of liberals to positively engage with CA compared to conservatives. This research also contributes to work on the globalization of companies and cross-cultural research by exploring the country and national culture dimensions that are more meaningful for companies as they consider their CA efforts and impact across countries and cultures.

For practice, this study offers actionable insights for companies navigating the complexity of sociopolitical engagement. It suggests that for CA to be effective, companies must carefully consider their stand on divisive issues, ensuring alignment with their target audiences’ political and societal values. Second, our analysis highlights the relevance of the controversy level of the sociopolitical issue at hand, with low controversial issues being better received by audiences. It seems that an optimal CA strategy for companies can be to focus on somewhat sociopolitical controversial issues—to create attention and differentiation—while avoiding extremely controversial issues but also “CSR like”—noncontroversial enough—issues. Third, examining demographic factors—especially age and cultural background—provides a nuanced understanding of the reaction to CA of different segments. Companies can expect younger audiences to react more positively to CA, which can be critical as companies target them as prospective employees and consumers. Further, the findings that power distance, individualism, and uncertainty avoidance moderate the response to CA may have implications for global companies as they consider their global CA strategy.

The implications of this research extend beyond the corporate world, offering insights for policymakers and societal leaders seeking to understand the complex interactions between corporate behavior, polarization, and public sentiment. For policymakers and society at large, the implications may be linked to governance and community relations, for example, having the potential for CA to act as a bridge within communities, depending on how well corporate positions resonate with or antagonize these communities’ morals and values. Policymakers could leverage these insights to better understand the impact of corporate behavior on social cohesion and to develop plans or frameworks that guide corporate engagement in sociopolitical issues in ways that promote unity and constructive dialogue.

### Limitations and future research directions

The limitations of our research, primarily the aggregated analysis, mostly relying on cross-sectional studies and Western-centric samples, point to multiple future research directions. First, future research can conduct non-US and cross-cultural studies of CA to better understand the sustained impact on company reputation and target audience attitudes and behaviors, particularly in non-Western contexts where different dynamics may be at play. Second, given the current focus on short-term stakeholder reaction and cross-sectional designs, subsequent studies could examine long-term outcomes for consumer behavior, company reputation, and society. In that context, very few studies identified a diminishing influence of CA over time (20, 54). Studying this in tandem with the target audience's political orientation may be valuable: Do conservatives/liberals react differently to CA over time? Third, there is also a valuable opportunity to explore further how CA affects not only market performance but also corporate governance, ethical business practices, and impact on the sociopolitical issues highlighted. Our initial findings about CA impact—mostly on cognitive and emotional outcomes, not behavioral ones—need to be further explored, especially if we want to learn how much of a societal impact CA actually has. Fourth, future research can also explore the different communication channels influencing CA outcomes. As sociopolitical issues continue to evolve and as the role of social media in CA expands, it is valuable to understand how companies navigate CA's complexities within different channels. Finally, research can explore the linkages between policymaking and companies’ CA, for example, what type of collaboration will enhance social welfare and the political or economic risks associated with such collaboration.

## Materials and methods

This study follows three main standard steps in meta-analysis (e.g. (57, 70)): (i) identifying all existing studies that empirically and quantitatively analyzed the relationship between CA and reactions to it; (ii) systematically coding the studies and converting statistics into correlation coefficients; (iii) estimating univariate and multivariate meta-analysis to understand the effects of CA.

### Data

To ensure a comprehensive understanding of CA and its response, we conducted an extensive search for empirical studies on CA to identify as many published and unpublished works as possible (56, 71, 72). We first searched for scholarly works on the central electronic databases: Web of Science, Scopus, JSTOR, and EBSCO. The search terms (in the title, abstract, and keywords fields) were: “corporate * activism” OR “corporate * advocacy” OR “brand * activism” OR “brand * advocacy” OR “CEO * activism” OR “CEO * advocacy”.^4^ Additionally, to include ongoing research, preliminary reports, and dissertations—critical for a developing field like CA—we also searched on Google Scholar, SSRN, ResearchGate, and dissertation and thesis databases. Data collection ended in January 2024. In this initial data collection phase, we applied no additional filters regarding the publication date, geographic focus, or study design, aiming to cast the broadest possible net to capture all relevant studies on the topic.

To be included in our analysis, the studies needed to: (i) directly address the relationship between CA and the reactions of target audiences^5^; (ii) provide quantifiable outcomes such as Pearson's correlation coefficients, regression coefficients, or *t* statistics, which are necessary for meta-analysis calculations (e.g. (56)); and (iii) contain data on at least one of our predefined variables of interest. This process yielded a comprehensive dataset comprising 72 scholarly works between 2013 and 2024. From these sources, we extracted data from 121 independent studies, amounting to 448 ES, as detailed in SI Appendix A, Table S4. Importantly, our sample size is similar to recent meta-analyses on similar fields (e.g. (70, 72)).

### Coding

Two researchers conducted the coding process independently in three phases to ensure thorough data capture and analysis. Initially, we captured the various outcomes associated with CA and categorized them into six broader categories encompassing the commonalities between the variables (see SI Appendix A, Table S1). Following this, the main factors that could influence the ES were registered. Among CA, target audience, and methodological characteristics, we coded 30 distinct variables (see SI Appendix A, Table S2, and SA4) used in the various meta-analytical models evaluated. Lastly, we focused on the ES derived from the studies. Most studies contain correlation coefficients; for the remainder, we use the statistical information reported, such as mean ratings and standard deviations, *t* tests, *F* tests, and regression coefficients. This information was then transformed into correlation coefficients (56, 70) (see SI Appendix A, Table S4).

### Meta-analysis

We first converted the statistics from each ES into r correlation coefficients to perform our meta-analysis. Subsequently, the correlations were transformed into Fisher's *z*-coefficient (56, 73). This step is recommended because the correlations are usually not normally distributed. Fisher's *z* transformation converts these correlations into a metric with a more consistent variance, making it easier and more reliable to analyze. The results were converted back to correlations for presentation.

Further, we adopted a random-effects approach. Unlike fixed-effects, the random-effects model does not assume uniformity in ES across studies. It recognizes and accommodates the inherent variability between studies, providing more reliable estimates considering the broader population of studies (74). Our dataset has multiple ES derived from a single study, so we expect dependencies within the data. ESs from the same study are typically more like each other than those from different studies. We incorporated a random intercept for each study to oversee this interdependence. This modeling choice ensures we capture the underlying variability within and between studies, offering more robust and accurate estimates (71).

Our analytical approach started with a univariate meta-analysis, examining each moderator individually. This approach is valuable as it allows for a granular understanding of the individual effect of each moderator without the confounding influences of other variables (75). However, such univariate analyses can be limiting, as it does not consider the simultaneous impact of multiple moderators, thus potentially leading to biased or incomplete insights (57, 71). To address this limitation and offer a more holistic and robust perspective, we proceeded to a hierarchical meta-regression (76–78). The meta-regression permits the inclusion of multiple moderators in a single analysis, thereby providing a more comprehensive understanding of their collective influence on the outcome (56). Consistent with recent meta-analyses (e.g. (57, 70)), we rely on the “metaphors” R package (55) for model estimations.

### Publication bias

To test for publication bias, we start with a funnel plot (see SI Appendix E) (56). While the plot appears symmetrical around the vertical axis, it shows a slight asymmetry, suggesting potential small-study effects bias. To further investigate this concern, we conducted Egger test (79). The results show a nonsignificant result (*z* = 0.3234, *P* = 0.7464), indicating no statistical evidence of a correlation between study ESs and their standard errors. This lack of correlation typically suggests that publication bias is not a significant factor in the dataset. Furthermore, the estimated ES (*b* = 0.0739) with its confidence interval including zero (−0.0018, 0.1496) when the standard error approaches zero implies no significant small-study effect. Consequently, the meta-analysis appears to be free of the systematic bias typically associated with the selective publication of studies based on their outcomes.

## Supplementary Material

pgae313_Supplementary_Data

## Data Availability

SI Appendix A, Table S4, includes all study data.

## Footnotes

1 We adopt the view that polarization is the process by which a society or group becomes divided into two or more contrasting groups or sets of opinions or beliefs, with differences increasingly aligning along a single dimension and reinforcing an “us” vs. “them,” whether controversial or not (1).

2 While SIT and affective polarization might not be related in their origins, SIT has been used to help understand some of the mechanisms of affective polarization. Indeed, work by Iyengar et al. (31, 32) has demonstrated that affective polarization can be explained through the lens of SIT by showing how individuals’ identification with political groups leads to stronger in-group favoritism and out-group animosity.

3 For additional results on country and national culture moderation without US observations see SI Appendix C, Table S7.

4 The asterisk (*) represents a wildcard character used to include any modifiers occurring between the words. For instance, “corporate * activism” captures phrases like “corporate social activism,” “corporate political activism,” or simply “corporate activism,” ensuring that our search results are comprehensive and include varied forms of CA and advocacy as expressed in the literature.

5 This includes studies that either directly measure CA’s impact on various stakeholders or use CA as a framing mechanism in experiments to assess how such activism influences public perceptions and behaviors. For instance, a study might measure changes in consumer trust following a corporation’s public statement or might use experimental designs to assess reactions to distinct types of corporate statements on sociopolitical issues.
