# Correction to: Understanding reaction to corporate activism: The moderating role of polarization

**DOI:** 10.1093/pnasnexus/pgaf380

**Published:** 2025-12-09

This is a correction to: Luiza Braga, Amir Grinstein, Matheus Tardin, Marcelo Perin, Understanding reaction to corporate activism: The moderating role of polarization, *PNAS Nexus*, Volume 3, Issue 10, October 2024, https://doi.org/10.1093/pnasnexus/pgae313

In the originally published online version of this manuscript, there were errors in the Supplementary data.

The errors concern references for the Sociopolitical Issues Controversy Level Index presented in Tables S2 and S3 of the Supplementary material.

In both tables, it was stated that “The index is derived from 2023 and 2022 Edelman Trust Barometer reports [3, 4] and Hydock et al.'s research [5]”.

This is not the correct reference. The correct source is the Global Strategy Group (2016, 2020, 2023) reports (1–3).

Please find the corrected Supplementary file with attached.

References are:

1. Global Strategy Group, “Business & Politics: Do They Mix? Third Annual Study” (2016).

2. Global Strategy Group, “Call Out Culture: Brands and Politics Collide in 2020. 7th Annual Business & Politics Study” (2020).

3. Global Strategy Group, “The Shifting Politics of Doing Good in America” (2023).

The data in the tables is accurate (only the wrong reference was used) so that, with the change in reference also, there is no affect on the analysis, results, or conclusions of the main document.

The errors have been outlined only in this correction notice to preserve the version of record.

