# Supplementary material for: Understanding reaction to corporate activism: The moderating role of polarization
Source: PNAS Nexus. 2024 Oct 15;3(10):pgae313. doi: 10.1093/pnasnexus/pgae313 (PMC11475398; doi:10.1093/pnasnexus/pgae313)
Supplement: pgae313_Supplementary_Data [file pgae313_supplementary_data.docx]

**
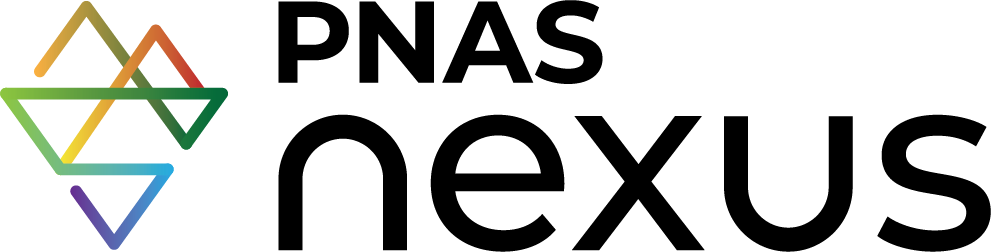
**

**Supplementary Information for**

Understanding Reaction to Corporate Activism: The Moderating Role of Polarization.

Luiza Braga, Amir Grinstein, Matheus Tardin, Marcelo Perin.

Amir Grinstein

Email: a.grinstein@northeastern.edu

**This PDF file includes:**

Table of Contents

Figure S1-S3

Tables S1 to S11

SI References

**Contents**

[Appendix A: Data and coding 3](#_Toc172275918)

[Table S1. Coding of dependent variables 3](#_Toc172275919)

[Table S2. Variables, codes, and descriptive statistics 6](#_Toc172275920)

[Table S3. Sociopolitical issues controversy level index 8](#_Toc172275921)

[Table S4. Meta-analysis data 9](#_Toc172275922)

[Appendix B: Univariate analysis 25](#_Toc172275923)

[Table S5. Univariate analysis 25](#_Toc172275924)

[Appendix C: Additional analyses 27](#_Toc172275925)

[Table S6. Country and national culture moderation analysis on response to CA 27](#_Toc172275926)

[Table S7. Country and national culture moderation without US 28](#_Toc172275927)

[Table S8. Dependent variable – by category of outcomes 29](#_Toc172275928)

[Appendix D: Sensitivity analysis 30](#_Toc172275929)

[Table S9. Models of response to CA with continuous target audience political orientation 30](#_Toc172275930)

[Table S10. Models of response to CA with unknown categories for main variables 31](#_Toc172275931)

[Table S11. Models of response to CA for an alternative measure of target audience alignment with CA 32](#_Toc172275932)

[Appendix E: Funnel plots 33](#_Toc172275933)

[Figure S1. Funnel plot of all effect sizes. 33](#_Toc172275934)

[Figure S2. Funnel plots of alignment between CA and audience political orientations (aligned vs. misaligned). 33](#_Toc172275935)

[Figure S3. Funnel plots of study methodology (experimental vs. observational). 34](#_Toc172275936)

[References 35](#_Toc172275937)

[References included in the meta-analysis (Table S4) 35](#_Toc172275938)

# Appendix A: Data and coding

To provide a deep understanding of the aggregated outcome measures, we detail the categorization and aggregation process of the original outcome variables in Table SA1. Each category represents a group of dependent variables systematically categorized based on their conceptual similarities and relevance. For instance, 'Ads and Social Media Engagement' encompasses variables related to consumers' interaction with brand advertisements and social media activities, such as ad attitude, click-through rates, and social media engagement intentions. Similarly, 'Cognitive and Attitudinal Reactions' includes variables reflecting consumers' attitudes and perceptions towards brands, such as brand attitude, brand loyalty, and corporate credibility. The table includes the number of times each unique outcome variable appeared in our sample.

We treated all these variables collectively in our main model (Table 1). By aggregating the variables, we aimed to capture the overall effect of CA without distinguishing between categories of outcomes. This approach provides a comprehensive view of the studied relationship and allows for generalizability. Still, in both Tables SB1 and SC3, we report separate analyses for each category of dependent variables.

## Table S1. Coding of dependent variables

| **Dependent variables dimensions** | **Original variable** | **K** |
| --- | --- | --- |
| Ads and social media engagement |  | **13** |
|  | Ad attitude | 3 |
|  | Attention | 1 |
|  | Click-through rates | 1 |
|  | Comment brand advertisement | 1 |
|  | Like brand advertising | 1 |
|  | Messages brand advertisement (comment length) | 1 |
|  | Online engagement | 2 |
|  | Sharing brand advertisement | 1 |
|  | Social media engagement intentions | 2 |
| Cognitive and attitudinal reactions |  | **179** |
|  | Attitude towards the brand/company | 10 |
|  | Attitudinal loyalty | 1 |
|  | Brand attitude | 41 |
|  | Brand authenticity | 19 |
|  | Brand autonomy | 1 |
|  | Brand awareness | 1 |
|  | Brand community | 1 |
|  | Brand consciousness | 1 |
|  | Brand credibility | 1 |
|  | Brand engagement | 1 |
|  | Brand equity | 12 |
|  | Brand image | 4 |
|  | Brand image change | 1 |
|  | Brand influence | 1 |
|  | Brand loyalty | 7 |
|  | Brand power | 1 |
|  | Brand recall | 1 |
|  | Brand recognition | 1 |
|  | Brand risk-taking | 1 |
|  | Brand trust | 2 |
|  | Cognitive dissonance | 5 |
|  | Cognitive engagement | 2 |
|  | Company attitudes | 1 |
|  | Consumer attitude | 1 |
|  | Consumer attitude towards to the company | 1 |
|  | Consumer-company identification | 9 |
|  | Consumer loyalty | 2 |
|  | Consumer organization relationships | 2 |
|  | Consumer skepticism | 1 |
|  | Corporate credibility | 1 |
|  | Corporate reputation | 12 |
|  | Distrust | 1 |
|  | Memory (encoding) | 2 |
|  | Organizational trust | 2 |
|  | Organization-public relationships | 2 |
|  | Positive opinion of the firm | 20 |
|  | Relational trust | 1 |
|  | Stakeholder perception of a company's social responsibility | 5 |
|  | Trust | 1 |
| Public's intentions and actions |  | **146** |
|  | Behavioral intention | 1 |
|  | Boycott | 13 |
|  | Brand choice | 22 |
|  | Buycott | 4 |
|  | Communicative behavior | 2 |
|  | Consumer visits to stores | 3 |
|  | Intention to boycott | 9 |
|  | Intention to buycott | 9 |
|  | Negative behavioral intentions | 1 |
|  | Negative word-of-mouth | 7 |
|  | Portfolio allocation | 9 |
|  | Positive word-of-mouth | 4 |
|  | Purchase | 4 |
|  | Purchase intention | 41 |
|  | Purchase loyalty | 1 |
|  | Sales | 3 |
|  | Willingness to pay | 7 |
|  | Word-of-mouth | 6 |
| Emotional reactions |  | **35** |
|  | Affective engagement/empathy | 2 |
|  | Brand feelings | 1 |
|  | Brand hate | 1 |
|  | Brand love | 19 |
|  | Condemning emotions | 5 |
|  | Emotional appeal | 2 |
|  | Praising emotions | 5 |
| Social and ethical engagement |  | **28** |
|  | Attitude change | 9 |
|  | Attitude change towards the issue/CA | 6 |
|  | Consumer advocacy | 2 |
|  | Digital activism | 1 |
|  | Donation amount to the cause | 3 |
|  | Issue advocacy | 3 |
|  | Political participation intentions | 2 |
|  | Public support for climate change policy | 1 |
|  | Supportive behavioral intentions | 1 |
| Workplace and employee perceptions |  | **47** |
|  | Desire to work for the firm | 20 |
|  | Employee motivation | 6 |
|  | Employer attractiveness | 6 |
|  | Employer brand attractiveness | 2 |
|  | Employer rejection | 3 |
|  | Intention to work | 2 |
|  | Job pursuit intention | 2 |
|  | Quality of required work | 6 |

**Note**: K = number of effect sizes; values in bold represent the sum of ES for each category.

## Table S2. Variables, codes, and descriptive statistics

| **Moderator group** | **Variable** | **Definition** | **Category** | **Codes** | **Mean** | **SD** |
| --- | --- | --- | --- | --- | --- | --- |
| CA characteristics | CA political leaning | When CA supports a liberal cause or opposes a conservative cause. | Liberal | 1 if yes, 0 otherwise | 0.55 | 0.50 |
|  | CA political leaning | When CA supports a conservative cause or opposes a liberal cause. | Conservative | 1 if yes, 0 otherwise | 0.11 | 0.32 |
|  | Sociopolitical issue controversy level | The extent to which a sociopolitical issue is perceived as less/more controversial/divisive and is expected by stakeholders to be addressed by a company. Lower values mean less people agree that companies should speak up about the specific issue, meaning that it is more controversial/divisive; while higher values mean more people agree that companies should speak up about the specific issue, indicating that it is less controversial/divisive. For the values of this variable, we utilized data from the Global Strategy Group (2016, 2020, 2023) reports (1–3). For more information see Table SA3. | Continuous | Scores in our sample ranges from 46 to 89 (only for the univariate analysis, we categorized issue controversy level into "Lower" and "Higher" groups based on the median value) | 70.82 | 9.09 |
| Target audience characteristics | Target audience political orientation | Whether the sample has a majority of liberal respondents, that is, when more than 50% of the participants hold or support liberal views, ideologies, or policies. This indicates a progressive or left-leaning stance. | Liberal | 1 if yes, 0 otherwise | 0.28 | 0.45 |
|  | Target audience political orientation | Whether the sample has a majority of conservative respondents, that is, when more than 50% of the participants hold or support conservative views, ideologies, or policies. This indicates a conservative or right-leaning stance. | Conservative | 1 if yes, 0 otherwise | 0.09 | 0.28 |
|  | Age | The average age of respondents in the sample. We replaced the few unknown values with the average value. | Continuous | (only for the univariate analysis we categorized age into "Younger" and "Older" groups based on the median value) | 35.01 | 6.91 |
|  | Gender | A variable indicating the study proportion of male respondents. In the few cases that it was not reported, we assumed a 50-50 male-female gender distribution (Lenk 2024). | Continuous | (only for the univariate analysis we categorized gender into "Male" if > .5 and "Female" if < .5) | 0.47 | 0.50 |
|  | Income | Whether the sample has over 50% of respondents with annual income greater than $50,000. . | Dummy | 1 if yes, 0 otherwise | 0.98 | 0.13 |
|  | Education | Whether most of the sample has a bachelor or higher degree. | Dummy | 1 if yes, 0 otherwise |  |  |
|  | Racial diversity | Whether the sample has a majority of white respondents. | Dummy | 1 if yes, 0 otherwise | 0.32 | 0.47 |
|  | Country of residence | Whether the sample was drawn from the United States. | Dummy | 1 if yes, 0 otherwise | 0.78 | 0.42 |
| Country and national culture characteristics | Cultural dimension: Power Distance | Measures the extent to which power differences are accepted and expected within a society. It reflects the degree to which less powerful members of organizations and institutions (like the family) accept and expect that power is distributed unequally. All four cultural dimensions follows Hofstede (4). | Continuous | Scores in our sample ranges from 31 to 80 | 43.93 | 8.09 |
|  | Cultural dimension: Individualism | The degree to which people in a society perceive the role of their individuality vs. the group they are part of. In more individualistic societies, the ties between individuals are loose, and everyone is expected to look after themselves and care less about the collective. | Continuous | Scores in our sample ranges from 5 to 100 | 60.49 | 10.09 |
|  | Cultural dimension: Masculinity | Reflects the distribution of roles between genders. It compares societies where masculine social values are predominant—emphasizing displays of success, performance, visible achievements, and financial gain—with societies where feminine social values prevail, prioritizing relationships over money, quality of life, environmental preservation, and helping others, especially those in need. | Continuous | Scores in our sample ranges from 5 to 95 | 59.94 | 13.09 |
|  | Cultural dimension: Uncertainty Avoidance | The degree of tolerance for uncertainty and ambiguity within a society. It indicates to what extent a culture programs its members to feel either uncomfortable or comfortable in unstructured situations. | Continuous | Scores in our sample ranges from 29 to 99 | 50.16 | 13.83 |
|  | Country political involvement | The extent to which the general population of a country is actively engaged in political processes, including voting, political discussions, and participation in public policy issues. It follows World Values Survey, round seven (5). | Continuous | Scores in our sample ranges from -0.337 to 0.421 | 0.12 | 1.55 |
|  | Country polarization level | This measures the extent of political division within a country, indicating how polarized public opinions are on major political and social issues. It follows 2023 Edelman Trust Barometer (6). | Continuous | Scores in our sample ranges from 1 to 4 | 3.75 | 0.57 |
| Methodological characteristics | Publication status | Whether the study is published in a peer-reviewed journal. | Dummy | 1 if yes, 0 otherwise | 0.72 | 0.45 |
|  | ABDC ranking | The ABDC ranking of the journal. Not Applicable for unranked journals or unpublished papers. | Dummy | A*, A, B, C, Not Applicable (for the multivariate analysis we used a continuous variation, from 0 for Not applicable to 4 for A*) | 1.96 | 1.52 |
|  | Top journal | The ranked journals belong to the Financial Times 50 Ranking. | Dummy | 1 if yes, 0 otherwise | 0.12 | 0.33 |
|  | Study method | Whether the study design was experimental. | Dummy | 1 if yes, 0 otherwise | 0.74 | 0.44 |
|  | Year | The year in which the study was published or completed. | Dummy | 2013 to 2024 (for the multivariate analysis, we used a continuous variation, from 2013 to 2024) | 2022 | 1.3 |

## Table S3. Sociopolitical issues controversy level index

| **Sociopolitical issues** | **ES** | **Index** | **Controversy dummy** |
| --- | --- | --- | --- |
| Abortion | 29 | 67 | Higher |
| BLM | 26 | 83 | Lower |
| Climate change | 11 | 86 | Lower |
| Diversity/Equality | 10 | 89 | Lower |
| Environment | 5 | 86 | Lower |
| Gender bathroom | 19 | 49 | Higher |
| Geopolitics | 4 | 80 | Lower |
| Gun control | 42 | 73 | Lower |
| Health/Pandemic | 10 | 68 | Higher |
| Immigration | 56 | 67 | Higher |
| LGBTQ+ | 59 | 63 | Higher |
| Politics | 50 | 83 | Lower |
| Racism | 15 | 83 | Lower |
| Religion | 5 | 49 | Higher |
| Same sex marriage | 17 | 63 | Higher |
| Various/Generic/NA | 90 | 70 | Unknown |
| *Note:* The index is derived from the Global Strategy Group (2016, 2020, 2023) reports (1–3). In cases where the index for the same issue changed between reports, we computed the average value. The index measures the expectation for companies/CEOs to publicly address specific issues, with higher values indicating less polarized opinions. The value for the category 'Various/Generic/NA' represents the average index value from this sample. 'ES' stands for Effect Sizes. | | | |

## Table S4. Meta-analysis data

| **1** | **2** | **3** | **4** | **5** | **6** | **7** | **8** | **9** | **10** | **11** | **12** | **13** | **14** | **15** | **16** | **17** | **18** | **19** | **20** | **21** | **22** | **23** | **24** | **25** | **26** | **27** | **28** | **29** | **30** |
| --- | --- | --- | --- | --- | --- | --- | --- | --- | --- | --- | --- | --- | --- | --- | --- | --- | --- | --- | --- | --- | --- | --- | --- | --- | --- | --- | --- | --- | --- |
| (Appels 2022) | S1 | 1 | 0 | -67 | 0 | 0 | 3.72 | 31 | 0.44 | 0 | 0 | 0 | US | 1 | 0.14 | 4.0 | 40 | 60 | 62 | 46 | 1 | A* | 4 | 1 | 1 | 2022 | W | 302 | 0.290 |
| (Appels 2022) | S1 | 1 | 0 | -67 | 0 | 0 | 3.72 | 31 | 0.44 | 0 | 0 | 0 | US | 1 | 0.14 | 4.0 | 40 | 60 | 62 | 46 | 1 | A* | 4 | 1 | 1 | 2022 | W | 302 | -0.432 |
| (Appels 2022) | S2 | 0 | 0 | -71 | 0 | 0 | 3.72 | 31 | 0.38 | 0 | 0 | 0 | US | 1 | 0.14 | 4.0 | 40 | 60 | 62 | 46 | 1 | A* | 4 | 1 | 1 | 2022 | W | 359 | -0.090 |
| (Appels 2022) | S2 | 0 | 0 | -71 | 0 | 0 | 3.72 | 31 | 0.38 | 0 | 0 | 0 | US | 1 | 0.14 | 4.0 | 40 | 60 | 62 | 46 | 1 | A* | 4 | 1 | 1 | 2022 | W | 359 | 0.020 |
| (Appels 2022) | S3A | 1 | 0 | -74 | 0 | 0 | 3.72 | 36 | 0.49 | 0 | 0 | 0 | US | 1 | 0.14 | 4.0 | 40 | 60 | 62 | 46 | 1 | A* | 4 | 1 | 1 | 2022 | W | 301 | 0.220 |
| (Appels 2022) | S3A | 0 | 1 | -74 | 0 | 0 | 3.72 | 36 | 0.49 | 0 | 0 | 0 | US | 1 | 0.14 | 4.0 | 40 | 60 | 62 | 46 | 1 | A* | 4 | 1 | 1 | 2022 | W | 301 | -0.310 |
| (Appels 2022) | S3A | 0 | 1 | -74 | 0 | 0 | 3.72 | 36 | 0.49 | 0 | 0 | 0 | US | 1 | 0.14 | 4.0 | 40 | 60 | 62 | 46 | 1 | A* | 4 | 1 | 1 | 2022 | W | 301 | 0.369 |
| (Appels 2022) | S3A | 1 | 0 | -74 | 0 | 0 | 3.72 | 36 | 0.49 | 0 | 0 | 0 | US | 1 | 0.14 | 4.0 | 40 | 60 | 62 | 46 | 1 | A* | 4 | 1 | 1 | 2022 | W | 301 | -0.462 |
| (Appels 2022) | S3B | 0 | 1 | -50 | 0 | 0 | 3.72 | 32 | 0.46 | 0 | 0 | 0 | US | 1 | 0.14 | 4.0 | 40 | 60 | 62 | 46 | 1 | A* | 4 | 1 | 1 | 2022 | W | 201 | -0.120 |
| (Appels 2022) | S3B | 0 | 1 | -50 | 0 | 0 | 3.72 | 32 | 0.46 | 0 | 0 | 0 | US | 1 | 0.14 | 4.0 | 40 | 60 | 62 | 46 | 1 | A* | 4 | 1 | 1 | 2022 | W | 201 | 0.203 |
| (Appels 2022) | S3B | 0 | 1 | -50 | 0 | 0 | 3.72 | 32 | 0.46 | 0 | 0 | 0 | US | 1 | 0.14 | 4.0 | 40 | 60 | 62 | 46 | 1 | A* | 4 | 1 | 1 | 2022 | W | 201 | 0.340 |
| (Atanga et al. 2022) | S1 | 1 | 0 | -71 | 0 | 0 | 3.72 | 35 | 0.75 | 0 | 0 | 0 | US | 1 | 0.14 | 4.0 | 40 | 60 | 62 | 46 | 1 | A* | 4 | 0 | 1 | 2022 | C | 88 | -0.254 |
| (Atanga et al. 2022) | S1 | 1 | 0 | -71 | 0 | 0 | 3.72 | 35 | 0.75 | 0 | 0 | 0 | US | 1 | 0.14 | 4.0 | 40 | 60 | 62 | 46 | 1 | A* | 4 | 0 | 1 | 2022 | C | 88 | 0.090 |
| (Atanga et al. 2022) | S1 | 1 | 0 | -71 | 0 | 0 | 3.72 | 35 | 0.75 | 0 | 0 | 0 | US | 1 | 0.14 | 4.0 | 40 | 60 | 62 | 46 | 1 | A* | 4 | 0 | 1 | 2022 | C | 88 | 0.228 |
| (Atanga et al. 2022) | S2 | 1 | 0 | -71 | 0 | 0 | 3.72 | 34 | 0.77 | 0 | 0 | 0 | US | 1 | 0.14 | 4.0 | 40 | 60 | 62 | 46 | 1 | A* | 4 | 0 | 1 | 2022 | C | 90 | 0.364 |
| (Atanga et al. 2022) | S2 | 1 | 0 | -71 | 0 | 0 | 3.72 | 34 | 0.77 | 0 | 0 | 0 | US | 1 | 0.14 | 4.0 | 40 | 60 | 62 | 46 | 1 | A* | 4 | 0 | 1 | 2022 | C | 90 | 0.603 |
| (Atanga and Mattila 2023) | S1 | 1 | 0 | -63 | 0 | 0 | 3.72 | 38 | 0.66 | 0 | 1 | 1 | US | 1 | 0.14 | 4.0 | 40 | 60 | 62 | 46 | 1 | A* | 4 | 0 | 1 | 2023 | C | 114 | 0.463 |
| (Atanga and Mattila 2023) | S1 | 1 | 0 | -63 | 0 | 0 | 3.72 | 38 | 0.66 | 0 | 1 | 1 | US | 1 | 0.14 | 4.0 | 40 | 60 | 62 | 46 | 1 | A* | 4 | 0 | 1 | 2023 | P | 114 | 0.407 |
| (Atanga and Mattila 2023) | S2 | 1 | 0 | -83 | 0 | 0 | 3.72 | 35 | 0.75 | 0 | 1 | 1 | US | 1 | 0.14 | 4.0 | 40 | 60 | 62 | 46 | 1 | A* | 4 | 0 | 1 | 2023 | P | 118 | 0.418 |
| (Burbano 2021) | - | 1 | 0 | -50 | 0 | 0 | 3.72 | 36 | 0.60 | 0 | 0 | 0 | Various | 0 | 0.00 | 2.6 | 70 | 30 | 40 | 30 | 1 | A* | 4 | 1 | 1 | 2021 | W | 239 | -0.118 |
| (Burbano 2021) | - | 0 | 1 | -50 | 0 | 0 | 3.72 | 36 | 0.60 | 0 | 0 | 0 | Various | 0 | 0.00 | 2.6 | 70 | 30 | 40 | 30 | 1 | A* | 4 | 1 | 1 | 2021 | W | 294 | -0.135 |
| (Burbano 2021) | - | 1 | 0 | -50 | 0 | 0 | 3.72 | 36 | 0.60 | 0 | 0 | 0 | Various | 0 | 0.00 | 2.6 | 70 | 30 | 40 | 30 | 1 | A* | 4 | 1 | 1 | 2021 | W | 239 | -0.148 |
| (Burbano 2021) | - | 0 | 1 | -50 | 0 | 0 | 3.72 | 36 | 0.60 | 0 | 0 | 0 | Various | 0 | 0.00 | 2.6 | 70 | 30 | 40 | 30 | 1 | A* | 4 | 1 | 1 | 2021 | W | 296 | -0.156 |
| (Burbano 2021) | - | 1 | 0 | -50 | 0 | 0 | 3.72 | 36 | 0.60 | 0 | 0 | 0 | Various | 0 | 0.00 | 2.6 | 70 | 30 | 40 | 30 | 1 | A* | 4 | 1 | 1 | 2021 | W | 296 | 0.027 |
| (Burbano 2021) | - | 1 | 0 | -50 | 0 | 0 | 3.72 | 36 | 0.60 | 0 | 0 | 0 | Various | 0 | 0.00 | 2.6 | 70 | 30 | 40 | 30 | 1 | A* | 4 | 1 | 1 | 2021 | W | 294 | -0.008 |
| (Burbano 2021) | - | 1 | 0 | -50 | 0 | 0 | 3.72 | 36 | 0.60 | 0 | 0 | 0 | Various | 0 | 0.00 | 2.6 | 70 | 30 | 40 | 30 | 1 | A* | 4 | 1 | 1 | 2021 | W | 195 | -0.038 |
| (Burbano 2021) | - | 1 | 0 | -50 | 0 | 0 | 3.72 | 36 | 0.60 | 0 | 0 | 0 | Various | 0 | 0.00 | 2.6 | 70 | 30 | 40 | 30 | 1 | A* | 4 | 1 | 1 | 2021 | W | 195 | 0.087 |
| (Burbano 2021) | - | 0 | 1 | -50 | 0 | 0 | 3.72 | 36 | 0.60 | 0 | 0 | 0 | Various | 0 | 0.00 | 2.6 | 70 | 30 | 40 | 30 | 1 | A* | 4 | 1 | 1 | 2021 | W | 239 | 0.049 |
| (Burbano 2021) | - | 0 | 1 | -50 | 0 | 0 | 3.72 | 36 | 0.60 | 0 | 0 | 0 | Various | 0 | 0.00 | 2.6 | 70 | 30 | 40 | 30 | 1 | A* | 4 | 1 | 1 | 2021 | W | 239 | 0.004 |
| (Burbano 2021) | - | 0 | 1 | -50 | 0 | 0 | 3.72 | 36 | 0.60 | 0 | 0 | 0 | Various | 0 | 0.00 | 2.6 | 70 | 30 | 40 | 30 | 1 | A* | 4 | 1 | 1 | 2021 | W | 195 | -0.050 |
| (Burbano 2021) | - | 0 | 1 | -50 | 0 | 0 | 3.72 | 36 | 0.60 | 0 | 0 | 0 | Various | 0 | 0.00 | 2.6 | 70 | 30 | 40 | 30 | 1 | A* | 4 | 1 | 1 | 2021 | W | 195 | -0.010 |
| (Hou and Poliquin 2022) | - | 1 | 0 | -74 | 1 | 0 | 3.94 | 36 | 0.50 | 0 | 0 | 0 | US | 1 | 0.14 | 4.0 | 40 | 60 | 62 | 46 | 1 | A* | 4 | 1 | 0 | 2022 | P | 1563885 | -0.062 |
| (Hou and Poliquin 2022) | - | 1 | 0 | -74 | 1 | 0 | 3.94 | 36 | 0.50 | 0 | 0 | 0 | US | 1 | 0.14 | 4.0 | 40 | 60 | 62 | 46 | 1 | A* | 4 | 1 | 0 | 2022 | P | 1563885 | 0.000 |
| (Hou and Poliquin 2022) | - | 1 | 0 | -74 | 0 | 0 | 3.72 | 36 | 0.50 | 0 | 0 | 0 | US | 1 | 0.14 | 4.0 | 40 | 60 | 62 | 46 | 1 | A* | 4 | 1 | 0 | 2022 | P | 34753 | -0.425 |
| (Hydock et al. 2020) | S1a | 0 | 0 | -50 | 0 | 0 | 3.72 | 20 | 0.53 | 0 | 0 | 0 | US | 1 | 0.14 | 4.0 | 40 | 60 | 62 | 46 | 1 | A* | 4 | 1 | 1 | 2020 | P | 509 | -0.124 |
| (Hydock et al. 2020) | S1a | 0 | 0 | -50 | 0 | 0 | 3.72 | 20 | 0.53 | 0 | 0 | 0 | US | 1 | 0.14 | 4.0 | 40 | 60 | 62 | 46 | 1 | A* | 4 | 1 | 1 | 2020 | P | 509 | -0.133 |
| (Hydock et al. 2020) | S1a | 0 | 0 | -50 | 0 | 0 | 3.72 | 20 | 0.53 | 0 | 0 | 0 | US | 1 | 0.14 | 4.0 | 40 | 60 | 62 | 46 | 1 | A* | 4 | 1 | 1 | 2020 | P | 263 | 0.140 |
| (Hydock et al. 2020) | S1a | 0 | 0 | -50 | 0 | 0 | 3.72 | 20 | 0.53 | 0 | 0 | 0 | US | 1 | 0.14 | 4.0 | 40 | 60 | 62 | 46 | 1 | A* | 4 | 1 | 1 | 2020 | P | 246 | -0.134 |
| (Hydock et al. 2020) | S1b | 0 | 0 | -76 | 0 | 0 | 3.72 | 34 | 0.29 | 0 | 0 | 0 | UK | 0 | 0.08 | 3.0 | 35 | 76 | 66 | 35 | 1 | A* | 4 | 1 | 1 | 2020 | P | 1007 | -0.168 |
| (Hydock et al. 2020) | S1b | 0 | 0 | -76 | 0 | 0 | 3.72 | 34 | 0.29 | 0 | 0 | 0 | UK | 0 | 0.08 | 3.0 | 35 | 76 | 66 | 35 | 1 | A* | 4 | 1 | 1 | 2020 | P | 521 | 0.165 |
| (Hydock et al. 2020) | S1b | 0 | 0 | -76 | 0 | 0 | 3.72 | 34 | 0.29 | 0 | 0 | 0 | UK | 0 | 0.08 | 3.0 | 35 | 76 | 66 | 35 | 1 | A* | 4 | 1 | 1 | 2020 | P | 486 | -0.173 |
| (Hydock et al. 2020) | S2 | 0 | 0 | -68 | 0 | 0 | 3.72 | 37 | 0.50 | 0 | 0 | 0 | US | 1 | 0.14 | 4.0 | 40 | 60 | 62 | 46 | 1 | A* | 4 | 1 | 1 | 2020 | P | 484 | -0.154 |
| (Hydock et al. 2020) | S2 | 0 | 0 | -68 | 0 | 0 | 3.72 | 37 | 0.50 | 0 | 0 | 0 | US | 1 | 0.14 | 4.0 | 40 | 60 | 62 | 46 | 1 | A* | 4 | 1 | 1 | 2020 | P | 484 | 0.334 |
| (Hydock et al. 2020) | S2 | 0 | 0 | -68 | 0 | 0 | 3.72 | 37 | 0.50 | 0 | 0 | 0 | US | 1 | 0.14 | 4.0 | 40 | 60 | 62 | 46 | 1 | A* | 4 | 1 | 1 | 2020 | P | 247 | 0.131 |
| (Hydock et al. 2020) | S2 | 0 | 0 | -68 | 0 | 0 | 3.72 | 37 | 0.50 | 0 | 0 | 0 | US | 1 | 0.14 | 4.0 | 40 | 60 | 62 | 46 | 1 | A* | 4 | 1 | 1 | 2020 | P | 237 | -0.189 |
| (Hydock et al. 2020) | S2 | 0 | 0 | -68 | 0 | 0 | 3.72 | 37 | 0.50 | 0 | 0 | 0 | US | 1 | 0.14 | 4.0 | 40 | 60 | 62 | 46 | 1 | A* | 4 | 1 | 1 | 2020 | P | 236 | 0.333 |
| (Hydock et al. 2020) | S2 | 0 | 0 | -68 | 0 | 0 | 3.72 | 37 | 0.50 | 0 | 0 | 0 | US | 1 | 0.14 | 4.0 | 40 | 60 | 62 | 46 | 1 | A* | 4 | 1 | 1 | 2020 | P | 248 | -0.387 |
| (Hydock et al. 2020) | S3 | 0 | 0 | -74 | 1 | 0 | 3.84 | 41 | 0.42 | 0 | 0 | 0 | US | 1 | 0.14 | 4.0 | 40 | 60 | 62 | 46 | 1 | A* | 4 | 1 | 1 | 2020 | P | 1002 | -0.030 |
| (Hydock et al. 2020) | S3 | 0 | 0 | -74 | 1 | 0 | 3.84 | 41 | 0.42 | 0 | 0 | 0 | US | 1 | 0.14 | 4.0 | 40 | 60 | 62 | 46 | 1 | A* | 4 | 1 | 1 | 2020 | P | 1002 | 0.136 |
| (Hydock et al. 2020) | S3 | 0 | 0 | -74 | 1 | 0 | 3.84 | 41 | 0.42 | 0 | 0 | 0 | US | 1 | 0.14 | 4.0 | 40 | 60 | 62 | 46 | 1 | A* | 4 | 1 | 1 | 2020 | P | 303 | 0.115 |
| (Hydock et al. 2020) | S3 | 0 | 0 | -74 | 1 | 0 | 3.84 | 41 | 0.42 | 0 | 0 | 0 | US | 1 | 0.14 | 4.0 | 40 | 60 | 62 | 46 | 1 | A* | 4 | 1 | 1 | 2020 | P | 300 | -0.254 |
| (Hydock et al. 2020) | S3 | 0 | 0 | -74 | 1 | 0 | 3.84 | 41 | 0.42 | 0 | 0 | 0 | US | 1 | 0.14 | 4.0 | 40 | 60 | 62 | 46 | 1 | A* | 4 | 1 | 1 | 2020 | P | 300 | -0.138 |
| (Hydock et al. 2020) | S3 | 0 | 0 | -74 | 1 | 0 | 3.84 | 41 | 0.42 | 0 | 0 | 0 | US | 1 | 0.14 | 4.0 | 40 | 60 | 62 | 46 | 1 | A* | 4 | 1 | 1 | 2020 | P | 299 | -0.094 |
| (Hydock et al. 2020) | S3 | 0 | 0 | -74 | 1 | 0 | 3.84 | 41 | 0.42 | 0 | 0 | 0 | US | 1 | 0.14 | 4.0 | 40 | 60 | 62 | 46 | 1 | A* | 4 | 1 | 1 | 2020 | P | 1002 | -0.171 |
| (Hydock et al. 2020) | S3 | 0 | 0 | -74 | 1 | 0 | 3.84 | 41 | 0.42 | 0 | 0 | 0 | US | 1 | 0.14 | 4.0 | 40 | 60 | 62 | 46 | 1 | A* | 4 | 1 | 1 | 2020 | P | 1002 | -0.206 |
| (Hydock et al. 2020) | S5 | 0 | 0 | -67 | 0 | 0 | 3.72 | 36 | 0.50 | 0 | 0 | 0 | US | 1 | 0.14 | 4.0 | 40 | 60 | 62 | 46 | 1 | A* | 4 | 1 | 1 | 2020 | A | 25514 | -0.012 |
| (Liaukonytė et al. 2023) | - | 0 | 1 | -80 | 1 | 0 | 2.99 | 36 | 0.50 | 0 | 0 | 1 | US | 1 | 0.14 | 4.0 | 40 | 60 | 62 | 46 | 1 | A* | 4 | 1 | 0 | 2023 | P | 2779338 | 0.001 |
| (Liaukonytė et al. 2023) | - | 0 | 1 | -80 | 1 | 0 | 2.99 | 36 | 0.50 | 0 | 0 | 1 | US | 1 | 0.14 | 4.0 | 40 | 60 | 62 | 46 | 1 | A* | 4 | 1 | 0 | 2023 | P | 2779338 | 0.001 |
| (Liaukonytė et al. 2023) | - | 0 | 1 | -80 | 1 | 0 | 2.99 | 36 | 0.50 | 0 | 0 | 1 | US | 1 | 0.14 | 4.0 | 40 | 60 | 62 | 46 | 1 | A* | 4 | 1 | 0 | 2023 | P | 2779338 | 0.003 |
| (Liaukonytė et al. 2023) | - | 0 | 1 | -80 | 1 | 0 | 2.99 | 36 | 0.50 | 0 | 0 | 1 | US | 1 | 0.14 | 4.0 | 40 | 60 | 62 | 46 | 1 | A* | 4 | 1 | 0 | 2023 | P | 2779338 | 0.005 |
| (Liaukonytė et al. 2023) | - | 0 | 1 | -80 | 1 | 0 | 2.99 | 36 | 0.50 | 0 | 0 | 1 | US | 1 | 0.14 | 4.0 | 40 | 60 | 62 | 46 | 1 | A* | 4 | 1 | 0 | 2023 | P | 2779338 | 0.003 |
| (Liaukonytė et al. 2023) | - | 0 | 1 | -80 | 1 | 0 | 2.99 | 36 | 0.50 | 0 | 0 | 1 | US | 1 | 0.14 | 4.0 | 40 | 60 | 62 | 46 | 1 | A* | 4 | 1 | 0 | 2023 | P | 2779338 | 0.004 |
| (Mukherjee and Althuizen 2020) | S1a | 0 | 0 | -68 | 0 | 0 | 3.72 | 36 | 0.61 | 0 | 0 | 0 | US | 1 | 0.14 | 4.0 | 40 | 60 | 62 | 46 | 1 | A* | 4 | 0 | 1 | 2020 | C | 154 | 0.052 |
| (Mukherjee and Althuizen 2020) | S1a | 0 | 0 | -68 | 0 | 0 | 3.72 | 36 | 0.61 | 0 | 0 | 0 | US | 1 | 0.14 | 4.0 | 40 | 60 | 62 | 46 | 1 | A* | 4 | 0 | 1 | 2020 | C | 154 | -0.459 |
| (Mukherjee and Althuizen 2020) | S1b | 0 | 1 | -68 | 0 | 0 | 3.72 | 24 | 0.50 | 0 | 0 | 0 | France | 0 | -0.28 | 3.0 | 68 | 74 | 43 | 86 | 1 | A* | 4 | 0 | 1 | 2020 | C | 115 | 0.121 |
| (Mukherjee and Althuizen 2020) | S1b | 0 | 1 | -68 | 0 | 0 | 3.72 | 24 | 0.50 | 0 | 0 | 0 | France | 0 | -0.28 | 3.0 | 68 | 74 | 43 | 86 | 1 | A* | 4 | 0 | 1 | 2020 | C | 115 | -0.382 |
| (Mukherjee and Althuizen 2020) | S1b | 0 | 1 | -68 | 0 | 0 | 3.72 | 24 | 0.50 | 0 | 0 | 0 | France | 0 | -0.28 | 3.0 | 68 | 74 | 43 | 86 | 1 | A* | 4 | 0 | 1 | 2020 | P | 115 | -0.412 |
| (Mukherjee and Althuizen 2020) | S2 | 1 | 0 | -67 | 0 | 0 | 3.72 | 29 | 0.63 | 0 | 0 | 0 | Portugal | 0 | -0.34 | 3.3 | 53 | 59 | 31 | 99 | 1 | A* | 4 | 0 | 1 | 2020 | C | 197 | -0.291 |
| (Mukherjee and Althuizen 2020) | S2 | 1 | 0 | -67 | 0 | 0 | 3.72 | 29 | 0.63 | 0 | 0 | 0 | Portugal | 0 | -0.34 | 3.3 | 53 | 59 | 31 | 99 | 1 | A* | 4 | 0 | 1 | 2020 | P | 197 | -0.217 |
| (Mukherjee and Althuizen 2020) | S3 | 0 | 0 | -68 | 0 | 0 | 3.72 | 41 | 0.45 | 0 | 0 | 0 | US | 1 | 0.14 | 4.0 | 40 | 60 | 62 | 46 | 1 | A* | 4 | 0 | 1 | 2020 | C | 210 | -0.137 |
| (Mukherjee and Althuizen 2020) | S3 | 0 | 0 | -68 | 0 | 0 | 3.72 | 41 | 0.45 | 0 | 0 | 0 | US | 1 | 0.14 | 4.0 | 40 | 60 | 62 | 46 | 1 | A* | 4 | 0 | 1 | 2020 | C | 210 | 0.103 |
| (Mukherjee and Althuizen 2020) | S3 | 0 | 0 | -68 | 0 | 0 | 3.72 | 41 | 0.45 | 0 | 0 | 0 | US | 1 | 0.14 | 4.0 | 40 | 60 | 62 | 46 | 1 | A* | 4 | 0 | 1 | 2020 | C | 210 | -0.529 |
| (Mukherjee and Althuizen 2020) | S3 | 0 | 0 | -68 | 0 | 0 | 3.72 | 41 | 0.45 | 0 | 0 | 0 | US | 1 | 0.14 | 4.0 | 40 | 60 | 62 | 46 | 1 | A* | 4 | 0 | 1 | 2020 | C | 210 | -0.670 |
| (Mukherjee and Althuizen 2020) | S4 | 0 | 0 | -50 | 0 | 0 | 3.72 | 37 | 0.55 | 0 | 0 | 0 | US | 1 | 0.14 | 4.0 | 40 | 60 | 62 | 46 | 1 | A* | 4 | 0 | 1 | 2020 | C | 151 | 0.128 |
| (Mukherjee and Althuizen 2020) | S4 | 0 | 0 | -50 | 0 | 0 | 3.72 | 37 | 0.55 | 0 | 0 | 0 | US | 1 | 0.14 | 4.0 | 40 | 60 | 62 | 46 | 1 | A* | 4 | 0 | 1 | 2020 | C | 153 | -0.652 |
| (Ahmad et al. 2022) | S1 | 1 | 0 | -83 | 0 | 0 | 3.72 | 20 | 0.44 | 0 | 0 | 0 | US | 1 | 0.14 | 4.0 | 40 | 60 | 62 | 46 | 1 | A | 3 | 0 | 1 | 2022 | C | 99 | 0.199 |
| (Ahmad et al. 2022) | S1 | 1 | 0 | -83 | 0 | 0 | 3.72 | 20 | 0.44 | 0 | 0 | 0 | US | 1 | 0.14 | 4.0 | 40 | 60 | 62 | 46 | 1 | A | 3 | 0 | 1 | 2022 | C | 66 | -0.282 |
| (Ahmad et al. 2022) | S1 | 1 | 0 | -83 | 0 | 0 | 3.72 | 20 | 0.44 | 0 | 0 | 0 | US | 1 | 0.14 | 4.0 | 40 | 60 | 62 | 46 | 1 | A | 3 | 0 | 1 | 2022 | C | 66 | 0.058 |
| (Ahmad et al. 2022) | S1 | 1 | 0 | -83 | 0 | 0 | 3.72 | 20 | 0.44 | 0 | 0 | 0 | US | 1 | 0.14 | 4.0 | 40 | 60 | 62 | 46 | 1 | A | 3 | 0 | 1 | 2022 | E | 99 | 0.297 |
| (Ahmad et al. 2022) | S1 | 1 | 0 | -83 | 0 | 0 | 3.72 | 20 | 0.44 | 0 | 0 | 0 | US | 1 | 0.14 | 4.0 | 40 | 60 | 62 | 46 | 1 | A | 3 | 0 | 1 | 2022 | E | 66 | -0.265 |
| (Ahmad et al. 2022) | S1 | 1 | 0 | -83 | 0 | 0 | 3.72 | 20 | 0.44 | 0 | 0 | 0 | US | 1 | 0.14 | 4.0 | 40 | 60 | 62 | 46 | 1 | A | 3 | 0 | 1 | 2022 | E | 66 | 0.260 |
| (Ahmad et al. 2022) | S2 | 1 | 0 | -83 | 0 | 0 | 3.72 | 20 | 0.49 | 0 | 0 | 0 | US | 1 | 0.14 | 4.0 | 40 | 60 | 62 | 46 | 1 | A | 3 | 0 | 1 | 2022 | C | 192 | 0.276 |
| (Ahmad et al. 2022) | S2 | 1 | 0 | -83 | 0 | 0 | 3.72 | 20 | 0.49 | 0 | 0 | 0 | US | 1 | 0.14 | 4.0 | 40 | 60 | 62 | 46 | 1 | A | 3 | 0 | 1 | 2022 | E | 192 | 0.239 |
| (Ahmad et al. 2022) | S3 | 1 | 0 | -83 | 0 | 0 | 3.72 | 20 | 0.46 | 0 | 0 | 0 | US | 1 | 0.14 | 4.0 | 40 | 60 | 62 | 46 | 1 | A | 3 | 0 | 1 | 2022 | C | 200 | 0.402 |
| (Ahmad et al. 2022) | S3 | 1 | 0 | -83 | 0 | 0 | 3.72 | 20 | 0.46 | 0 | 0 | 0 | US | 1 | 0.14 | 4.0 | 40 | 60 | 62 | 46 | 1 | A | 3 | 0 | 1 | 2022 | C | 66 | 0.138 |
| (Ahmad et al. 2022) | S3 | 1 | 0 | -83 | 0 | 0 | 3.72 | 20 | 0.46 | 0 | 0 | 0 | US | 1 | 0.14 | 4.0 | 40 | 60 | 62 | 46 | 1 | A | 3 | 0 | 1 | 2022 | C | 66 | 0.256 |
| (Ahmad et al. 2022) | S3 | 1 | 0 | -83 | 0 | 0 | 3.72 | 20 | 0.46 | 0 | 0 | 0 | US | 1 | 0.14 | 4.0 | 40 | 60 | 62 | 46 | 1 | A | 3 | 0 | 1 | 2022 | E | 200 | 0.543 |
| (Ahmad et al. 2022) | S3 | 1 | 0 | -83 | 0 | 0 | 3.72 | 20 | 0.46 | 0 | 0 | 0 | US | 1 | 0.14 | 4.0 | 40 | 60 | 62 | 46 | 1 | A | 3 | 0 | 1 | 2022 | E | 66 | 0.143 |
| (Ahmad et al. 2022) | S3 | 1 | 0 | -83 | 0 | 0 | 3.72 | 20 | 0.46 | 0 | 0 | 0 | US | 1 | 0.14 | 4.0 | 40 | 60 | 62 | 46 | 1 | A | 3 | 0 | 1 | 2022 | E | 66 | 0.233 |
| (Ahmad et al. 2022) | S4 | 1 | 0 | -74 | 0 | 0 | 3.72 | 20 | 0.47 | 0 | 0 | 0 | US | 1 | 0.14 | 4.0 | 40 | 60 | 62 | 46 | 1 | A | 3 | 0 | 1 | 2022 | C | 352 | 0.134 |
| (Ahmad et al. 2022) | S4 | 1 | 0 | -74 | 0 | 0 | 3.72 | 20 | 0.47 | 0 | 0 | 0 | US | 1 | 0.14 | 4.0 | 40 | 60 | 62 | 46 | 1 | A | 3 | 0 | 1 | 2022 | C | 352 | 0.180 |
| (Ahmad et al. 2022) | S4 | 1 | 0 | -74 | 0 | 0 | 3.72 | 20 | 0.47 | 0 | 0 | 0 | US | 1 | 0.14 | 4.0 | 40 | 60 | 62 | 46 | 1 | A | 3 | 0 | 1 | 2022 | C | 58 | -0.406 |
| (Ahmad et al. 2022) | S4 | 1 | 0 | -74 | 0 | 0 | 3.72 | 20 | 0.47 | 0 | 0 | 0 | US | 1 | 0.14 | 4.0 | 40 | 60 | 62 | 46 | 1 | A | 3 | 0 | 1 | 2022 | C | 58 | -0.052 |
| (Ahmad et al. 2022) | S4 | 1 | 0 | -74 | 0 | 0 | 3.72 | 20 | 0.47 | 0 | 0 | 0 | US | 1 | 0.14 | 4.0 | 40 | 60 | 62 | 46 | 1 | A | 3 | 0 | 1 | 2022 | C | 58 | 0.198 |
| (Ahmad et al. 2022) | S4 | 1 | 0 | -74 | 0 | 0 | 3.72 | 20 | 0.47 | 0 | 0 | 0 | US | 1 | 0.14 | 4.0 | 40 | 60 | 62 | 46 | 1 | A | 3 | 0 | 1 | 2022 | C | 58 | 0.257 |
| (Ahmad et al. 2022) | S4 | 1 | 0 | -74 | 0 | 0 | 3.72 | 20 | 0.47 | 0 | 0 | 0 | US | 1 | 0.14 | 4.0 | 40 | 60 | 62 | 46 | 1 | A | 3 | 0 | 1 | 2022 | E | 352 | 0.164 |
| (Ahmad et al. 2022) | S4 | 1 | 0 | -74 | 0 | 0 | 3.72 | 20 | 0.47 | 0 | 0 | 0 | US | 1 | 0.14 | 4.0 | 40 | 60 | 62 | 46 | 1 | A | 3 | 0 | 1 | 2022 | E | 352 | 0.505 |
| (Ahmad et al. 2022) | S4 | 1 | 0 | -74 | 0 | 0 | 3.72 | 20 | 0.47 | 0 | 0 | 0 | US | 1 | 0.14 | 4.0 | 40 | 60 | 62 | 46 | 1 | A | 3 | 0 | 1 | 2022 | E | 58 | -0.405 |
| (Ahmad et al. 2022) | S4 | 1 | 0 | -74 | 0 | 0 | 3.72 | 20 | 0.47 | 0 | 0 | 0 | US | 1 | 0.14 | 4.0 | 40 | 60 | 62 | 46 | 1 | A | 3 | 0 | 1 | 2022 | E | 58 | 0.063 |
| (Ahmad et al. 2022) | S4 | 1 | 0 | -74 | 0 | 0 | 3.72 | 20 | 0.47 | 0 | 0 | 0 | US | 1 | 0.14 | 4.0 | 40 | 60 | 62 | 46 | 1 | A | 3 | 0 | 1 | 2022 | E | 58 | 0.184 |
| (Ahmad et al. 2022) | S4 | 1 | 0 | -74 | 0 | 0 | 3.72 | 20 | 0.47 | 0 | 0 | 0 | US | 1 | 0.14 | 4.0 | 40 | 60 | 62 | 46 | 1 | A | 3 | 0 | 1 | 2022 | E | 58 | 0.213 |
| (Ahmad et al. 2022) | S5 | 1 | 0 | -63 | 0 | 0 | 3.72 | 36 | 0.50 | 0 | 0 | 0 | US | 1 | 0.14 | 4.0 | 40 | 60 | 62 | 46 | 1 | A | 3 | 0 | 1 | 2022 | C | 601 | 0.095 |
| (Ahmad et al. 2022) | S5 | 1 | 0 | -63 | 0 | 0 | 3.72 | 36 | 0.50 | 0 | 0 | 0 | US | 1 | 0.14 | 4.0 | 40 | 60 | 62 | 46 | 1 | A | 3 | 0 | 1 | 2022 | C | 601 | 0.204 |
| (Ahmad et al. 2022) | S5 | 1 | 0 | -63 | 0 | 0 | 3.72 | 36 | 0.50 | 0 | 0 | 0 | US | 1 | 0.14 | 4.0 | 40 | 60 | 62 | 46 | 1 | A | 3 | 0 | 1 | 2022 | C | 100 | -0.330 |
| (Ahmad et al. 2022) | S5 | 1 | 0 | -63 | 0 | 0 | 3.72 | 36 | 0.50 | 0 | 0 | 0 | US | 1 | 0.14 | 4.0 | 40 | 60 | 62 | 46 | 1 | A | 3 | 0 | 1 | 2022 | C | 100 | -0.075 |
| (Ahmad et al. 2022) | S5 | 1 | 0 | -63 | 0 | 0 | 3.72 | 36 | 0.50 | 0 | 0 | 0 | US | 1 | 0.14 | 4.0 | 40 | 60 | 62 | 46 | 1 | A | 3 | 0 | 1 | 2022 | C | 100 | 0.158 |
| (Ahmad et al. 2022) | S5 | 1 | 0 | -63 | 0 | 0 | 3.72 | 36 | 0.50 | 0 | 0 | 0 | US | 1 | 0.14 | 4.0 | 40 | 60 | 62 | 46 | 1 | A | 3 | 0 | 1 | 2022 | C | 100 | 0.165 |
| (Ahmad et al. 2022) | S5 | 1 | 0 | -63 | 0 | 0 | 3.72 | 36 | 0.50 | 0 | 0 | 0 | US | 1 | 0.14 | 4.0 | 40 | 60 | 62 | 46 | 1 | A | 3 | 0 | 1 | 2022 | E | 601 | 0.100 |
| (Ahmad et al. 2022) | S5 | 1 | 0 | -63 | 0 | 0 | 3.72 | 36 | 0.50 | 0 | 0 | 0 | US | 1 | 0.14 | 4.0 | 40 | 60 | 62 | 46 | 1 | A | 3 | 0 | 1 | 2022 | E | 601 | 0.341 |
| (Ahmad et al. 2022) | S5 | 1 | 0 | -63 | 0 | 0 | 3.72 | 36 | 0.50 | 0 | 0 | 0 | US | 1 | 0.14 | 4.0 | 40 | 60 | 62 | 46 | 1 | A | 3 | 0 | 1 | 2022 | E | 100 | -0.282 |
| (Ahmad et al. 2022) | S5 | 1 | 0 | -63 | 0 | 0 | 3.72 | 36 | 0.50 | 0 | 0 | 0 | US | 1 | 0.14 | 4.0 | 40 | 60 | 62 | 46 | 1 | A | 3 | 0 | 1 | 2022 | E | 100 | -0.033 |
| (Ahmad et al. 2022) | S5 | 1 | 0 | -63 | 0 | 0 | 3.72 | 36 | 0.50 | 0 | 0 | 0 | US | 1 | 0.14 | 4.0 | 40 | 60 | 62 | 46 | 1 | A | 3 | 0 | 1 | 2022 | E | 100 | 0.160 |
| (Ahmad et al. 2022) | S5 | 1 | 0 | -63 | 0 | 0 | 3.72 | 36 | 0.50 | 0 | 0 | 0 | US | 1 | 0.14 | 4.0 | 40 | 60 | 62 | 46 | 1 | A | 3 | 0 | 1 | 2022 | E | 100 | 0.191 |
| (Chatterji and Toffel 2019) | S1 | 1 | 0 | -63 | 0 | 0 | 3.72 | 36 | 0.52 | 1 | 0 | 1 | US | 1 | 0.14 | 4.0 | 40 | 60 | 62 | 46 | 1 | A | 3 | 0 | 1 | 2019 | P | 227 | -0.070 |
| (Chatterji and Toffel 2019) | S1 | 1 | 0 | -63 | 0 | 0 | 3.72 | 36 | 0.52 | 1 | 0 | 1 | US | 1 | 0.14 | 4.0 | 40 | 60 | 62 | 46 | 1 | A | 3 | 0 | 1 | 2019 | P | 1465 | 0.115 |
| (Chatterji and Toffel 2019) | S1 | 1 | 0 | -63 | 0 | 0 | 3.72 | 36 | 0.52 | 1 | 0 | 1 | US | 1 | 0.14 | 4.0 | 40 | 60 | 62 | 46 | 1 | A | 3 | 0 | 1 | 2019 | P | 146 | 0.351 |
| (Chatterji and Toffel 2019) | S2 | 1 | 0 | -87 | 0 | 0 | 3.72 | 36 | 0.53 | 0 | 0 | 1 | US | 1 | 0.14 | 4.0 | 40 | 60 | 62 | 46 | 1 | A | 3 | 0 | 1 | 2019 | S | 4615 | -0.043 |
| (Chu et al. 2022) | - | 0 | 0 | -71 | 0 | 0 | 3.72 | 36 | 0.40 | 0 | 0 | 0 | US | 1 | 0.14 | 4.0 | 40 | 60 | 62 | 46 | 1 | A | 3 | 0 | 0 | 2022 | C | 349 | 0.610 |
| (Chu et al. 2022) | - | 0 | 0 | -71 | 0 | 0 | 3.72 | 36 | 0.40 | 0 | 0 | 0 | US | 1 | 0.14 | 4.0 | 40 | 60 | 62 | 46 | 1 | A | 3 | 0 | 0 | 2022 | C | 349 | 0.740 |
| (Chu et al. 2022) | - | 0 | 0 | -71 | 0 | 0 | 3.72 | 36 | 0.40 | 0 | 0 | 0 | US | 1 | 0.14 | 4.0 | 40 | 60 | 62 | 46 | 1 | A | 3 | 0 | 0 | 2022 | C | 349 | 0.590 |
| (Chu et al. 2022) | - | 0 | 0 | -71 | 0 | 0 | 3.72 | 36 | 0.40 | 0 | 0 | 0 | US | 1 | 0.14 | 4.0 | 40 | 60 | 62 | 46 | 1 | A | 3 | 0 | 0 | 2022 | C | 349 | 0.720 |
| (Chu et al. 2022) | - | 0 | 0 | -71 | 0 | 0 | 3.72 | 36 | 0.40 | 0 | 0 | 0 | US | 1 | 0.14 | 4.0 | 40 | 60 | 62 | 46 | 1 | A | 3 | 0 | 0 | 2022 | P | 349 | 0.580 |
| (Chu et al. 2022) | - | 0 | 0 | -71 | 0 | 0 | 3.72 | 36 | 0.40 | 0 | 0 | 0 | US | 1 | 0.14 | 4.0 | 40 | 60 | 62 | 46 | 1 | A | 3 | 0 | 0 | 2022 | P | 349 | 0.660 |
| (DiRusso et al. 2022) | - | 1 | 0 | -68 | 1 | 0 | 3.39 | 40 | 0.52 | 0 | 1 | 1 | US | 1 | 0.14 | 4.0 | 40 | 60 | 62 | 46 | 1 | A | 3 | 0 | 1 | 2022 | A | 508 | -0.109 |
| (DiRusso et al. 2022) | - | 1 | 0 | -68 | 1 | 0 | 3.39 | 40 | 0.52 | 0 | 1 | 1 | US | 1 | 0.14 | 4.0 | 40 | 60 | 62 | 46 | 1 | A | 3 | 0 | 1 | 2022 | A | 508 | 0.057 |
| (DiRusso et al. 2022) | - | 1 | 0 | -68 | 1 | 0 | 3.39 | 40 | 0.52 | 0 | 1 | 1 | US | 1 | 0.14 | 4.0 | 40 | 60 | 62 | 46 | 1 | A | 3 | 0 | 1 | 2022 | C | 508 | -0.110 |
| (DiRusso et al. 2022) | - | 1 | 0 | -68 | 1 | 0 | 3.39 | 40 | 0.52 | 0 | 1 | 1 | US | 1 | 0.14 | 4.0 | 40 | 60 | 62 | 46 | 1 | A | 3 | 0 | 1 | 2022 | C | 508 | -0.107 |
| (DiRusso et al. 2022) | - | 1 | 0 | -68 | 1 | 0 | 3.39 | 40 | 0.52 | 0 | 1 | 1 | US | 1 | 0.14 | 4.0 | 40 | 60 | 62 | 46 | 1 | A | 3 | 0 | 1 | 2022 | C | 508 | -0.087 |
| (DiRusso et al. 2022) | - | 1 | 0 | -68 | 1 | 0 | 3.39 | 40 | 0.52 | 0 | 1 | 1 | US | 1 | 0.14 | 4.0 | 40 | 60 | 62 | 46 | 1 | A | 3 | 0 | 1 | 2022 | P | 508 | -0.110 |
| (DiRusso et al. 2022) | - | 1 | 0 | -68 | 1 | 0 | 3.39 | 40 | 0.52 | 0 | 1 | 1 | US | 1 | 0.14 | 4.0 | 40 | 60 | 62 | 46 | 1 | A | 3 | 0 | 1 | 2022 | S | 508 | -0.139 |
| (DiRusso et al. 2022) | - | 1 | 0 | -68 | 1 | 0 | 3.39 | 40 | 0.52 | 0 | 1 | 1 | US | 1 | 0.14 | 4.0 | 40 | 60 | 62 | 46 | 1 | A | 3 | 0 | 1 | 2022 | S | 508 | 0.110 |
| (Fernandes 2020) | S1 | 1 | 0 | -83 | 0 | 1 | 4.19 | 36 | 0.63 | 0 | 0 | 0 | US | 1 | 0.14 | 4.0 | 40 | 60 | 62 | 46 | 1 | A | 3 | 0 | 1 | 2020 | P | 207 | -0.257 |
| (Fernandes 2020) | S1 | 1 | 0 | -83 | 0 | 1 | 4.19 | 36 | 0.63 | 0 | 0 | 0 | US | 1 | 0.14 | 4.0 | 40 | 60 | 62 | 46 | 1 | A | 3 | 0 | 1 | 2020 | P | 207 | -0.286 |
| (Fernandes 2020) | S2 | 1 | 0 | -71 | 0 | 1 | 4.26 | 32 | 0.48 | 0 | 0 | 0 | US | 1 | 0.14 | 4.0 | 40 | 60 | 62 | 46 | 1 | A | 3 | 0 | 1 | 2020 | P | 385 | -0.267 |
| (Fernandes 2020) | S2 | 1 | 0 | -71 | 0 | 1 | 4.26 | 32 | 0.48 | 0 | 0 | 0 | US | 1 | 0.14 | 4.0 | 40 | 60 | 62 | 46 | 1 | A | 3 | 0 | 1 | 2020 | P | 385 | -0.313 |
| (Fernandes 2020) | S2 | 0 | 1 | -71 | 0 | 1 | 4.26 | 32 | 0.48 | 0 | 0 | 0 | US | 1 | 0.14 | 4.0 | 40 | 60 | 62 | 46 | 1 | A | 3 | 0 | 1 | 2020 | P | 385 | 0.137 |
| (Fernandes 2020) | S2 | 0 | 1 | -71 | 0 | 1 | 4.26 | 32 | 0.48 | 0 | 0 | 0 | US | 1 | 0.14 | 4.0 | 40 | 60 | 62 | 46 | 1 | A | 3 | 0 | 1 | 2020 | P | 385 | 0.289 |
| (Fernandes 2020) | S3 | 0 | 0 | -71 | 0 | 1 | 4.05 | 36 | 0.43 | 0 | 0 | 0 | US | 1 | 0.14 | 4.0 | 40 | 60 | 62 | 46 | 1 | A | 3 | 0 | 1 | 2020 | P | 791 | 0.146 |
| (Fernandes 2020) | S3 | 0 | 0 | -71 | 0 | 1 | 4.05 | 36 | 0.43 | 0 | 0 | 0 | US | 1 | 0.14 | 4.0 | 40 | 60 | 62 | 46 | 1 | A | 3 | 0 | 1 | 2020 | P | 791 | 0.311 |
| (Fernandes 2020) | S3 | 0 | 0 | -71 | 0 | 1 | 4.05 | 36 | 0.43 | 0 | 0 | 0 | US | 1 | 0.14 | 4.0 | 40 | 60 | 62 | 46 | 1 | A | 3 | 0 | 1 | 2020 | P | 791 | -0.086 |
| (Hamelberg et al. 2024) | S2 | 0 | 0 | -80 | 1 | 0 | 2.94 | 31 | 0.56 | 0 | 0 | 0 | US | 1 | 0.14 | 4.0 | 40 | 60 | 62 | 46 | 1 | A | 3 | 0 | 1 | 2024 | S | 608 | 0.101 |
| (Jin et al. 2023) | - | 0 | 0 | -71 | 0 | 0 | 3.72 | 28 | 0.64 | 0 | 0 | 0 | US | 1 | 0.14 | 4.0 | 40 | 60 | 62 | 46 | 0 | A | 3 | 0 | 0 | 2023 | C | 737 | 0.137 |
| (Jin et al. 2023) | - | 0 | 0 | -71 | 0 | 0 | 3.72 | 28 | 0.64 | 0 | 0 | 0 | US | 1 | 0.14 | 4.0 | 40 | 60 | 62 | 46 | 0 | A | 3 | 0 | 0 | 2023 | C | 737 | 0.141 |
| (Jin et al. 2023) | - | 0 | 0 | -71 | 0 | 0 | 3.72 | 28 | 0.64 | 0 | 0 | 0 | US | 1 | 0.14 | 4.0 | 40 | 60 | 62 | 46 | 0 | A | 3 | 0 | 0 | 2023 | P | 737 | -0.008 |
| (Jin et al. 2023) | - | 0 | 0 | -71 | 0 | 0 | 3.72 | 28 | 0.64 | 0 | 0 | 0 | US | 1 | 0.14 | 4.0 | 40 | 60 | 62 | 46 | 0 | A | 3 | 0 | 0 | 2023 | P | 737 | 0.057 |
| (Jungblut and Johnen 2021) | S1 | 0 | 0 | -89 | 1 | 0 | 2.88 | 31 | 0.47 | 0 | 0 | 0 | Germany | 0 | 0.32 | 3.0 | 35 | 79 | 66 | 65 | 1 | A | 3 | 0 | 1 | 2021 | C | 133 | -0.377 |
| (Jungblut and Johnen 2021) | S2 | 0 | 0 | -71 | 1 | 0 | 3.97 | 44 | 0.39 | 0 | 0 | 0 | US | 1 | 0.14 | 4.0 | 40 | 60 | 62 | 46 | 1 | A | 3 | 0 | 1 | 2021 | P | 737 | 0.173 |
| (Jungblut and Johnen 2021) | S2 | 0 | 0 | -71 | 1 | 0 | 3.97 | 44 | 0.39 | 0 | 0 | 0 | US | 1 | 0.14 | 4.0 | 40 | 60 | 62 | 46 | 1 | A | 3 | 0 | 1 | 2021 | P | 737 | -0.435 |
| (Lee et al. 2023) | - | 1 | 0 | -87 | 0 | 0 | 3.72 | 44 | 0.38 | 0 | 0 | 0 | UK | 0 | 0.08 | 3.0 | 35 | 76 | 66 | 35 | 1 | A | 3 | 0 | 0 | 2023 | C | 518 | -0.750 |
| (Lee et al .2023) | - | 1 | 0 | -87 | 0 | 0 | 3.72 | 44 | 0.38 | 0 | 0 | 0 | UK | 0 | 0.08 | 3.0 | 35 | 76 | 66 | 35 | 1 | A | 3 | 0 | 0 | 2023 | C | 518 | 0.090 |
| (Li et al. 2021) | - | 1 | 0 | -83 | 1 | 0 | 3.81 | 46 | 0.56 | 0 | 0 | 1 | US | 1 | 0.14 | 4.0 | 40 | 60 | 62 | 46 | 1 | A | 3 | 0 | 0 | 2021 | P | 121 | 0.320 |
| (Li et al. 2021) | - | 1 | 0 | -83 | 1 | 0 | 3.81 | 46 | 0.56 | 0 | 0 | 1 | US | 1 | 0.14 | 4.0 | 40 | 60 | 62 | 46 | 1 | A | 3 | 0 | 0 | 2021 | P | 121 | 0.270 |
| (Li et al. 2021) | - | 1 | 0 | -83 | 1 | 0 | 3.81 | 46 | 0.56 | 0 | 0 | 1 | US | 1 | 0.14 | 4.0 | 40 | 60 | 62 | 46 | 1 | A | 3 | 0 | 0 | 2021 | P | 121 | -0.260 |
| (Li et al. 2021) | - | 1 | 0 | -83 | 1 | 0 | 3.81 | 46 | 0.56 | 0 | 0 | 1 | US | 1 | 0.14 | 4.0 | 40 | 60 | 62 | 46 | 1 | A | 3 | 0 | 0 | 2021 | P | 121 | -0.280 |
| (Lim and Young 2021) | - | 1 | 0 | -63 | 0 | 0 | 3.72 | 36 | 0.50 | 0 | 0 | 0 | US | 1 | 0.14 | 4.0 | 40 | 60 | 62 | 46 | 1 | A | 3 | 0 | 0 | 2021 | C | 800 | -0.091 |
| (Lim and Young 2021) | - | 1 | 0 | -63 | 0 | 0 | 3.72 | 36 | 0.50 | 0 | 0 | 0 | US | 1 | 0.14 | 4.0 | 40 | 60 | 62 | 46 | 1 | A | 3 | 0 | 0 | 2021 | C | 800 | 0.072 |
| (Lim and Young 2021) | - | 1 | 0 | -63 | 0 | 0 | 3.72 | 36 | 0.50 | 0 | 0 | 0 | US | 1 | 0.14 | 4.0 | 40 | 60 | 62 | 46 | 1 | A | 3 | 0 | 0 | 2021 | C | 800 | 0.090 |
| (Lim and Young 2021) | - | 1 | 0 | -63 | 0 | 0 | 3.72 | 36 | 0.50 | 0 | 0 | 0 | US | 1 | 0.14 | 4.0 | 40 | 60 | 62 | 46 | 1 | A | 3 | 0 | 0 | 2021 | C | 800 | 0.355 |
| (Nguyen et al. 2022) | - | 1 | 0 | -87 | 0 | 0 | 3.72 | 22 | 0.31 | 0 | 0 | 0 | Vietnam | 0 | -0.12 | 1.5 | 54.4 | 58.06 | 49.33 | 61.26 | 1 | A | 3 | 0 | 0 | 2022 | C | 1133 | 0.674 |
| (Nguyen et al. 2022) | - | 1 | 0 | -87 | 0 | 0 | 3.72 | 22 | 0.31 | 0 | 0 | 0 | Vietnam | 0 | -0.12 | 1.5 | 54.4 | 58.06 | 49.33 | 61.26 | 1 | A | 3 | 0 | 0 | 2022 | C | 1133 | 0.517 |
| (Nguyen et al. 2022) | - | 1 | 0 | -87 | 0 | 0 | 3.72 | 22 | 0.31 | 0 | 0 | 0 | Vietnam | 0 | -0.12 | 1.5 | 54.4 | 58.06 | 49.33 | 61.26 | 1 | A | 3 | 0 | 0 | 2022 | C | 1133 | 0.612 |
| (Overton et al. 2021) | - | 1 | 0 | -89 | 1 | 0 | 3.74 | 47 | 0.49 | 1 | 0 | 1 | US | 1 | 0.14 | 4.0 | 40 | 60 | 62 | 46 | 1 | A | 3 | 0 | 1 | 2022 | P | 219 | -0.136 |
| (Overton et al. 2021) | - | 1 | 0 | -89 | 1 | 0 | 3.74 | 47 | 0.49 | 1 | 0 | 1 | US | 1 | 0.14 | 4.0 | 40 | 60 | 62 | 46 | 1 | A | 3 | 0 | 1 | 2022 | P | 219 | 0.189 |
| (Overton et al. 2021) | - | 1 | 0 | -89 | 1 | 0 | 3.74 | 47 | 0.49 | 1 | 0 | 1 | US | 1 | 0.14 | 4.0 | 40 | 60 | 62 | 46 | 1 | A | 3 | 0 | 1 | 2022 | S | 219 | 0.003 |
| (Overton et al. 2021) | - | 1 | 0 | -89 | 1 | 0 | 3.74 | 47 | 0.49 | 1 | 0 | 1 | US | 1 | 0.14 | 4.0 | 40 | 60 | 62 | 46 | 1 | A | 3 | 0 | 1 | 2022 | S | 219 | 0.152 |
| (Tan 2023) | S1 | 1 | 0 | -63 | 0 | 0 | 3.72 | 35 | 0.41 | 0 | 0 | 0 | Japan | 0 | 0.18 | 3.0 | 54 | 62 | 95 | 92 | 1 | A | 3 | 0 | 0 | 2023 | P | 309 | -0.071 |
| (Tan 2023) | S1 | 1 | 0 | -63 | 0 | 0 | 3.72 | 35 | 0.41 | 0 | 0 | 0 | Japan | 0 | 0.18 | 3.0 | 54 | 62 | 95 | 92 | 1 | A | 3 | 0 | 0 | 2023 | P | 309 | 0.244 |
| (Tan 2023) | S1 | 1 | 0 | -63 | 0 | 0 | 3.72 | 35 | 0.41 | 0 | 0 | 0 | Japan | 0 | 0.18 | 3.0 | 54 | 62 | 95 | 92 | 1 | A | 3 | 0 | 0 | 2023 | P | 309 | 0.424 |
| (Tan 2023) | S1 | 1 | 0 | -63 | 0 | 0 | 3.72 | 35 | 0.41 | 0 | 0 | 0 | Japan | 0 | 0.18 | 3.0 | 54 | 62 | 95 | 92 | 1 | A | 3 | 0 | 0 | 2023 | P | 309 | 0.031 |
| (Tan 2023) | S1 | 1 | 0 | -63 | 0 | 0 | 3.72 | 35 | 0.41 | 0 | 0 | 0 | Japan | 0 | 0.18 | 3.0 | 54 | 62 | 95 | 92 | 1 | A | 3 | 0 | 0 | 2023 | P | 309 | 0.068 |
| (Tan 2023) | S1 | 1 | 0 | -63 | 0 | 0 | 3.72 | 35 | 0.41 | 0 | 0 | 0 | Japan | 0 | 0.18 | 3.0 | 54 | 62 | 95 | 92 | 1 | A | 3 | 0 | 0 | 2023 | P | 309 | 0.485 |
| (Tan 2023) | S2 | 1 | 0 | -63 | 0 | 0 | 3.72 | 49 | 0.51 | 0 | 0 | 0 | Japan | 0 | 0.18 | 3.0 | 54 | 62 | 95 | 92 | 1 | A | 3 | 0 | 0 | 2023 | P | 305 | -0.087 |
| (Tan 2023) | S2 | 1 | 0 | -63 | 0 | 0 | 3.72 | 49 | 0.51 | 0 | 0 | 0 | Japan | 0 | 0.18 | 3.0 | 54 | 62 | 95 | 92 | 1 | A | 3 | 0 | 0 | 2023 | P | 305 | 0.089 |
| (Tan 2023) | S2 | 1 | 0 | -63 | 0 | 0 | 3.72 | 49 | 0.51 | 0 | 0 | 0 | Japan | 0 | 0.18 | 3.0 | 54 | 62 | 95 | 92 | 1 | A | 3 | 0 | 0 | 2023 | P | 305 | 0.388 |
| (Tan 2023) | S2 | 1 | 0 | -63 | 0 | 0 | 3.72 | 49 | 0.51 | 0 | 0 | 0 | Japan | 0 | 0.18 | 3.0 | 54 | 62 | 95 | 92 | 1 | A | 3 | 0 | 0 | 2023 | P | 305 | -0.201 |
| (Tan 2023) | S2 | 1 | 0 | -63 | 0 | 0 | 3.72 | 49 | 0.51 | 0 | 0 | 0 | Japan | 0 | 0.18 | 3.0 | 54 | 62 | 95 | 92 | 1 | A | 3 | 0 | 0 | 2023 | P | 305 | 0.264 |
| (Tan 2023) | S2 | 1 | 0 | -63 | 0 | 0 | 3.72 | 49 | 0.51 | 0 | 0 | 0 | Japan | 0 | 0.18 | 3.0 | 54 | 62 | 95 | 92 | 1 | A | 3 | 0 | 0 | 2023 | P | 305 | 0.464 |
| (Tan 2023) | S3 | 1 | 0 | -63 | 0 | 0 | 3.72 | 21 | 0.69 | 0 | 0 | 0 | Japan | 0 | 0.18 | 3.0 | 54 | 62 | 95 | 92 | 1 | A | 3 | 0 | 0 | 2023 | P | 270 | 0.018 |
| (Tan 2023) | S3 | 1 | 0 | -63 | 0 | 0 | 3.72 | 21 | 0.69 | 0 | 0 | 0 | Japan | 0 | 0.18 | 3.0 | 54 | 62 | 95 | 92 | 1 | A | 3 | 0 | 0 | 2023 | P | 270 | 0.134 |
| (Tan 2023) | S3 | 1 | 0 | -63 | 0 | 0 | 3.72 | 21 | 0.69 | 0 | 0 | 0 | Japan | 0 | 0.18 | 3.0 | 54 | 62 | 95 | 92 | 1 | A | 3 | 0 | 0 | 2023 | P | 270 | 0.387 |
| (Tan 2023) | S3 | 1 | 0 | -63 | 0 | 0 | 3.72 | 21 | 0.69 | 0 | 0 | 0 | Japan | 0 | 0.18 | 3.0 | 54 | 62 | 95 | 92 | 1 | A | 3 | 0 | 0 | 2023 | P | 270 | -0.154 |
| (Tan 2023) | S3 | 1 | 0 | -63 | 0 | 0 | 3.72 | 21 | 0.69 | 0 | 0 | 0 | Japan | 0 | 0.18 | 3.0 | 54 | 62 | 95 | 92 | 1 | A | 3 | 0 | 0 | 2023 | P | 270 | 0.199 |
| (Tan 2023) | S3 | 1 | 0 | -63 | 0 | 0 | 3.72 | 21 | 0.69 | 0 | 0 | 0 | Japan | 0 | 0.18 | 3.0 | 54 | 62 | 95 | 92 | 1 | A | 3 | 0 | 0 | 2023 | P | 270 | 0.295 |
| (Tsai et al. 2023) | - | 0 | 0 | -71 | 1 | 0 | 3.92 | 49 | 0.47 | 1 | 0 | 1 | US | 1 | 0.14 | 4.0 | 40 | 60 | 62 | 46 | 1 | A | 3 | 0 | 0 | 2023 | P | 817 | 0.430 |
| (Tsai et al. 2023) | - | 0 | 0 | -71 | 1 | 0 | 3.92 | 49 | 0.47 | 1 | 0 | 1 | US | 1 | 0.14 | 4.0 | 40 | 60 | 62 | 46 | 1 | A | 3 | 0 | 0 | 2023 | P | 817 | 0.540 |
| (Tsai et al. 2023) | - | 0 | 0 | -71 | 1 | 0 | 3.92 | 49 | 0.47 | 1 | 0 | 1 | US | 1 | 0.14 | 4.0 | 40 | 60 | 62 | 46 | 1 | A | 3 | 0 | 0 | 2023 | S | 817 | 0.500 |
| (Tsai et al. 2023) | - | 0 | 0 | -71 | 1 | 0 | 3.92 | 49 | 0.47 | 1 | 0 | 1 | US | 1 | 0.14 | 4.0 | 40 | 60 | 62 | 46 | 1 | A | 3 | 0 | 0 | 2023 | S | 817 | 0.570 |
| (Wannow et al. 2023) | S1 | 0 | 0 | -68 | 0 | 1 | 4.15 | 37 | 0.64 | 0 | 0 | 0 | US | 1 | 0.14 | 4.0 | 40 | 60 | 62 | 46 | 1 | A | 3 | 0 | 1 | 2023 | C | 179 | -0.046 |
| (Wannow et al. 2023) | S1 | 0 | 0 | -68 | 0 | 1 | 4.15 | 37 | 0.64 | 0 | 0 | 0 | US | 1 | 0.14 | 4.0 | 40 | 60 | 62 | 46 | 1 | A | 3 | 0 | 1 | 2023 | C | 179 | -0.188 |
| (Wannow et al. 2023) | S1 | 0 | 0 | -68 | 0 | 1 | 4.15 | 37 | 0.64 | 0 | 0 | 0 | US | 1 | 0.14 | 4.0 | 40 | 60 | 62 | 46 | 1 | A | 3 | 0 | 1 | 2023 | E | 179 | 0.042 |
| (Wannow et al. 2023) | S1 | 0 | 0 | -68 | 0 | 1 | 4.15 | 37 | 0.64 | 0 | 0 | 0 | US | 1 | 0.14 | 4.0 | 40 | 60 | 62 | 46 | 1 | A | 3 | 0 | 1 | 2023 | E | 179 | 0.279 |
| (Wannow et al. 2023) | S1 | 0 | 0 | -68 | 0 | 1 | 4.15 | 37 | 0.64 | 0 | 0 | 0 | US | 1 | 0.14 | 4.0 | 40 | 60 | 62 | 46 | 1 | A | 3 | 0 | 1 | 2023 | E | 179 | -0.291 |
| (Wannow et al. 2023) | S1 | 0 | 0 | -68 | 0 | 1 | 4.15 | 37 | 0.64 | 0 | 0 | 0 | US | 1 | 0.14 | 4.0 | 40 | 60 | 62 | 46 | 1 | A | 3 | 0 | 1 | 2023 | E | 179 | -0.363 |
| (Wannow et al. 2023) | S2 | 0 | 0 | -67 | 1 | 0 | 3.84 | 37 | 0.64 | 0 | 0 | 0 | US | 1 | 0.14 | 4.0 | 40 | 60 | 62 | 46 | 1 | A | 3 | 0 | 1 | 2023 | C | 244 | -0.049 |
| (Wannow et al. 2023) | S2 | 0 | 0 | -67 | 1 | 0 | 3.84 | 37 | 0.64 | 0 | 0 | 0 | US | 1 | 0.14 | 4.0 | 40 | 60 | 62 | 46 | 1 | A | 3 | 0 | 1 | 2023 | C | 244 | -0.155 |
| (Wannow et al. 2023) | S2 | 0 | 0 | -67 | 1 | 0 | 3.84 | 37 | 0.64 | 0 | 0 | 0 | US | 1 | 0.14 | 4.0 | 40 | 60 | 62 | 46 | 1 | A | 3 | 0 | 1 | 2023 | E | 244 | 0.026 |
| (Wannow et al. 2023) | S2 | 0 | 0 | -67 | 1 | 0 | 3.84 | 37 | 0.64 | 0 | 0 | 0 | US | 1 | 0.14 | 4.0 | 40 | 60 | 62 | 46 | 1 | A | 3 | 0 | 1 | 2023 | E | 244 | 0.126 |
| (Wannow et al. 2023) | S2 | 0 | 0 | -67 | 1 | 0 | 3.84 | 37 | 0.64 | 0 | 0 | 0 | US | 1 | 0.14 | 4.0 | 40 | 60 | 62 | 46 | 1 | A | 3 | 0 | 1 | 2023 | E | 244 | -0.169 |
| (Wannow et al. 2023) | S2 | 0 | 0 | -67 | 1 | 0 | 3.84 | 37 | 0.64 | 0 | 0 | 0 | US | 1 | 0.14 | 4.0 | 40 | 60 | 62 | 46 | 1 | A | 3 | 0 | 1 | 2023 | E | 244 | -0.245 |
| (Wannow et al. 2023) | S3 | 0 | 0 | -68 | 0 | 1 | 4.29 | 39 | 0.55 | 0 | 0 | 0 | US | 1 | 0.14 | 4.0 | 40 | 60 | 62 | 46 | 1 | A | 3 | 0 | 1 | 2023 | E | 205 | 0.303 |
| (Wannow et al. 2023) | S3 | 0 | 0 | -68 | 0 | 1 | 4.29 | 39 | 0.55 | 0 | 0 | 0 | US | 1 | 0.14 | 4.0 | 40 | 60 | 62 | 46 | 1 | A | 3 | 0 | 1 | 2023 | E | 205 | 0.273 |
| (Wannow et al. 2023) | S3 | 0 | 0 | -68 | 0 | 1 | 4.29 | 39 | 0.55 | 0 | 0 | 0 | US | 1 | 0.14 | 4.0 | 40 | 60 | 62 | 46 | 1 | A | 3 | 0 | 1 | 2023 | S | 205 | -0.220 |
| (Weber et al. 2023) | S2 | 0 | 0 | -68 | 0 | 1 | 4.1 | 51 | 0.35 | 0 | 0 | 1 | US | 1 | 0.14 | 4.0 | 40 | 60 | 62 | 46 | 1 | A | 3 | 0 | 1 | 2023 | C | 785 | 0.461 |
| (Weber et al. 2023) | S2 | 0 | 0 | -68 | 0 | 1 | 4.31 | 51 | 0.35 | 0 | 0 | 1 | US | 1 | 0.14 | 4.0 | 40 | 60 | 62 | 46 | 1 | A | 3 | 0 | 1 | 2023 | C | 763 | -0.460 |
| (Weber et al. 2023) | S3 | 0 | 0 | -67 | 1 | 0 | 3.42 | 39 | 0.55 | 0 | 0 | 1 | US | 1 | 0.14 | 4.0 | 40 | 60 | 62 | 46 | 1 | A | 3 | 0 | 1 | 2023 | C | 504 | 0.470 |
| (Weber et al. 2023) | S3 | 0 | 0 | -67 | 1 | 0 | 3.42 | 39 | 0.55 | 0 | 0 | 1 | US | 1 | 0.14 | 4.0 | 40 | 60 | 62 | 46 | 1 | A | 3 | 0 | 1 | 2023 | P | 504 | 0.377 |
| (Zhou et al. 2023) | S1 | 1 | 0 | -89 | 1 | 0 | 3.13 | 35 | 0.60 | 0 | 0 | 1 | US | 1 | 0.14 | 4.0 | 40 | 60 | 62 | 46 | 1 | A | 3 | 0 | 1 | 2023 | A | 334 | -0.384 |
| (Zhou et al. 2023) | S1 | 1 | 0 | -89 | 1 | 0 | 3.13 | 35 | 0.60 | 0 | 0 | 1 | US | 1 | 0.14 | 4.0 | 40 | 60 | 62 | 46 | 1 | A | 3 | 0 | 1 | 2023 | P | 334 | -0.251 |
| (Zhou et al. 2023) | S2 | 1 | 0 | -68 | 1 | 0 | 3.27 | 35 | 0.56 | 0 | 0 | 1 | US | 1 | 0.14 | 4.0 | 40 | 60 | 62 | 46 | 1 | A | 3 | 0 | 1 | 2023 | A | 320 | -0.451 |
| (Zhou et al. 2023) | S2 | 1 | 0 | -68 | 1 | 0 | 3.27 | 35 | 0.56 | 0 | 0 | 1 | US | 1 | 0.14 | 4.0 | 40 | 60 | 62 | 46 | 1 | A | 3 | 0 | 1 | 2023 | P | 320 | -0.229 |
| (Garg and Saluja 2022) | S1 | 1 | 0 | -83 | 0 | 0 | 3.72 | 37 | 0.57 | 0 | 0 | 0 | US | 1 | 0.14 | 4.0 | 40 | 60 | 62 | 46 | 1 | B | 2 | 0 | 1 | 2022 | C | 252 | 0.412 |
| (Garg and Saluja 2022) | S1 | 1 | 0 | -83 | 0 | 0 | 3.72 | 37 | 0.57 | 0 | 0 | 0 | US | 1 | 0.14 | 4.0 | 40 | 60 | 62 | 46 | 1 | B | 2 | 0 | 1 | 2022 | P | 252 | 0.379 |
| (Garg and Saluja 2022) | S2 | 1 | 0 | -68 | 0 | 0 | 3.72 | 41 | 0.58 | 0 | 0 | 0 | US | 1 | 0.14 | 4.0 | 40 | 60 | 62 | 46 | 1 | B | 2 | 0 | 1 | 2022 | C | 217 | 0.514 |
| (Garg and Saluja 2022) | S2 | 0 | 1 | -68 | 0 | 0 | 3.72 | 41 | 0.58 | 0 | 0 | 0 | US | 1 | 0.14 | 4.0 | 40 | 60 | 62 | 46 | 1 | B | 2 | 0 | 1 | 2022 | C | 217 | -0.494 |
| (Garg and Saluja 2022) | S2 | 1 | 0 | -68 | 0 | 0 | 3.72 | 41 | 0.58 | 0 | 0 | 0 | US | 1 | 0.14 | 4.0 | 40 | 60 | 62 | 46 | 1 | B | 2 | 0 | 1 | 2022 | P | 217 | 0.287 |
| (Garg and Saluja 2022) | S2 | 0 | 1 | -68 | 0 | 0 | 3.72 | 41 | 0.58 | 0 | 0 | 0 | US | 1 | 0.14 | 4.0 | 40 | 60 | 62 | 46 | 1 | B | 2 | 0 | 1 | 2022 | P | 217 | -0.486 |
| (Garg and Saluja 2022) | S3 | 1 | 0 | -68 | 0 | 0 | 3.72 | 41 | 0.60 | 0 | 0 | 0 | US | 1 | 0.14 | 4.0 | 40 | 60 | 62 | 46 | 1 | B | 2 | 0 | 1 | 2022 | C | 489 | -0.121 |
| (Garg and Saluja 2022) | S3 | 1 | 0 | -68 | 0 | 0 | 3.72 | 41 | 0.60 | 0 | 0 | 0 | US | 1 | 0.14 | 4.0 | 40 | 60 | 62 | 46 | 1 | B | 2 | 0 | 1 | 2022 | C | 489 | 0.310 |
| (Garg and Saluja 2022) | S3 | 1 | 0 | -68 | 0 | 0 | 3.72 | 41 | 0.60 | 0 | 0 | 0 | US | 1 | 0.14 | 4.0 | 40 | 60 | 62 | 46 | 1 | B | 2 | 0 | 1 | 2022 | C | 489 | -0.260 |
| (Garg and Saluja 2022) | S3 | 1 | 0 | -68 | 0 | 0 | 3.72 | 41 | 0.60 | 0 | 0 | 0 | US | 1 | 0.14 | 4.0 | 40 | 60 | 62 | 46 | 1 | B | 2 | 0 | 1 | 2022 | C | 489 | -0.358 |
| (Garg and Saluja 2022) | S3 | 1 | 0 | -68 | 0 | 0 | 3.72 | 41 | 0.60 | 0 | 0 | 0 | US | 1 | 0.14 | 4.0 | 40 | 60 | 62 | 46 | 1 | B | 2 | 0 | 1 | 2022 | P | 489 | -0.238 |
| (Garg and Saluja 2022) | S3 | 1 | 0 | -68 | 0 | 0 | 3.72 | 41 | 0.60 | 0 | 0 | 0 | US | 1 | 0.14 | 4.0 | 40 | 60 | 62 | 46 | 1 | B | 2 | 0 | 1 | 2022 | P | 489 | -0.113 |
| (Garg and Saluja 2022) | S3 | 1 | 0 | -68 | 0 | 0 | 3.72 | 41 | 0.60 | 0 | 0 | 0 | US | 1 | 0.14 | 4.0 | 40 | 60 | 62 | 46 | 1 | B | 2 | 0 | 1 | 2022 | P | 489 | 0.228 |
| (Garg and Saluja 2022) | S3 | 1 | 0 | -68 | 0 | 0 | 3.72 | 41 | 0.60 | 0 | 0 | 0 | US | 1 | 0.14 | 4.0 | 40 | 60 | 62 | 46 | 1 | B | 2 | 0 | 1 | 2022 | P | 489 | -0.377 |
| (Haupt et al. 2023) | S1 | 0 | 0 | -68 | 0 | 1 | 4.39 | 39 | 0.55 | 0 | 0 | 0 | US | 1 | 0.14 | 4.0 | 40 | 60 | 62 | 46 | 1 | B | 2 | 0 | 1 | 2023 | C | 262 | 0.256 |
| (Haupt et al. 2023) | S1 | 0 | 0 | -68 | 0 | 1 | 4.39 | 39 | 0.55 | 0 | 0 | 0 | US | 1 | 0.14 | 4.0 | 40 | 60 | 62 | 46 | 1 | B | 2 | 0 | 1 | 2023 | C | 262 | -0.301 |
| (Haupt et al. 2023) | S2 | 0 | 0 | -68 | 0 | 1 | 4.53 | 37 | 0.64 | 0 | 0 | 0 | US | 1 | 0.14 | 4.0 | 40 | 60 | 62 | 46 | 1 | B | 2 | 0 | 1 | 2023 | P | 354 | -0.319 |
| (Haupt et al. 2023) | S3 | 0 | 0 | -67 | 0 | 1 | 4.25 | 37 | 0.66 | 0 | 0 | 0 | US | 1 | 0.14 | 4.0 | 40 | 60 | 62 | 46 | 1 | B | 2 | 0 | 1 | 2023 | C | 383 | -0.222 |
| (Hong and Li 2020) | - | 1 | 0 | -63 | 1 | 0 | 3.5 | 35 | 0.47 | 0 | 0 | 1 | US | 1 | 0.14 | 4.0 | 40 | 60 | 62 | 46 | 1 | B | 2 | 0 | 0 | 2020 | C | 482 | 0.113 |
| (Hong and Li 2020) | - | 1 | 0 | -63 | 1 | 0 | 3.5 | 35 | 0.47 | 0 | 0 | 1 | US | 1 | 0.14 | 4.0 | 40 | 60 | 62 | 46 | 1 | B | 2 | 0 | 0 | 2020 | C | 482 | 0.125 |
| (Hong and Li 2020) | - | 1 | 0 | -63 | 1 | 0 | 3.5 | 35 | 0.47 | 0 | 0 | 1 | US | 1 | 0.14 | 4.0 | 40 | 60 | 62 | 46 | 1 | B | 2 | 0 | 0 | 2020 | P | 482 | -0.147 |
| (Hong and Li 2020) | - | 1 | 0 | -63 | 1 | 0 | 3.5 | 35 | 0.47 | 0 | 0 | 1 | US | 1 | 0.14 | 4.0 | 40 | 60 | 62 | 46 | 1 | B | 2 | 0 | 0 | 2020 | P | 482 | 0.054 |
| (Hong and Li 2020) | - | 1 | 0 | -63 | 1 | 0 | 3.5 | 35 | 0.47 | 0 | 0 | 1 | US | 1 | 0.14 | 4.0 | 40 | 60 | 62 | 46 | 1 | B | 2 | 0 | 0 | 2020 | P | 482 | 0.161 |
| (Hong and Li 2020) | - | 1 | 0 | -63 | 1 | 0 | 3.5 | 35 | 0.47 | 0 | 0 | 1 | US | 1 | 0.14 | 4.0 | 40 | 60 | 62 | 46 | 1 | B | 2 | 0 | 0 | 2020 | P | 482 | -0.080 |
| (Hong and Li 2021) | - | 1 | 0 | -74 | 0 | 1 | 4.39 | 38 | 0.47 | 0 | 1 | 1 | US | 1 | 0.14 | 4.0 | 40 | 60 | 62 | 46 | 1 | B | 2 | 0 | 1 | 2021 | C | 122 | 0.366 |
| (Hong and Li 2021) | - | 1 | 0 | -74 | 0 | 1 | 4.67 | 38 | 0.47 | 0 | 1 | 1 | US | 1 | 0.14 | 4.0 | 40 | 60 | 62 | 46 | 1 | B | 2 | 0 | 1 | 2021 | C | 122 | 0.177 |
| (Hong and Li 2021) | - | 1 | 0 | -74 | 0 | 1 | 4.67 | 38 | 0.47 | 0 | 1 | 1 | US | 1 | 0.14 | 4.0 | 40 | 60 | 62 | 46 | 1 | B | 2 | 0 | 1 | 2021 | P | 122 | 0.067 |
| (Hong and Li 2021) | - | 1 | 0 | -74 | 0 | 1 | 4.67 | 38 | 0.47 | 0 | 1 | 1 | US | 1 | 0.14 | 4.0 | 40 | 60 | 62 | 46 | 1 | B | 2 | 0 | 1 | 2021 | P | 122 | 0.215 |
| (Lee and Tao 2021) | - | 0 | 0 | -71 | 1 | 0 | 3.75 | 43 | 0.53 | 0 | 1 | 1 | US | 1 | 0.14 | 4.0 | 40 | 60 | 62 | 46 | 1 | B | 2 | 0 | 0 | 2022 | P | 417 | 0.738 |
| (Lee 2023) | - | 1 | 0 | -74 | 1 | 0 | 3.75 | 37 | 0.60 | 0 | 0 | 1 | US | 1 | 0.14 | 4.0 | 40 | 60 | 62 | 46 | 1 | B | 2 | 0 | 1 | 2023 | S | 505 | 0.371 |
| (Lee 2023) | - | 1 | 0 | -74 | 1 | 0 | 3.75 | 37 | 0.60 | 0 | 0 | 1 | US | 1 | 0.14 | 4.0 | 40 | 60 | 62 | 46 | 1 | B | 2 | 0 | 1 | 2023 | S | 505 | -0.298 |
| (Overton et al. 2020) | - | 1 | 0 | -83 | 1 | 0 | 3.85 | 53 | 0.56 | 1 | 0 | 1 | US | 1 | 0.14 | 4.0 | 40 | 60 | 62 | 46 | 1 | B | 2 | 0 | 0 | 2020 | P | 373 | 0.297 |
| (Overton et al. 2020) | - | 1 | 0 | -83 | 1 | 0 | 3.85 | 53 | 0.56 | 1 | 0 | 1 | US | 1 | 0.14 | 4.0 | 40 | 60 | 62 | 46 | 1 | B | 2 | 0 | 0 | 2020 | P | 373 | 0.384 |
| (Overton et al. 2020) | - | 1 | 0 | -83 | 1 | 0 | 3.85 | 53 | 0.56 | 1 | 0 | 1 | US | 1 | 0.14 | 4.0 | 40 | 60 | 62 | 46 | 1 | B | 2 | 0 | 0 | 2020 | P | 373 | 0.423 |
| (Park 2022) | - | 0 | 0 | -71 | 0 | 0 | 3.72 | 36 | 0.44 | 0 | 1 | 1 | US | 1 | 0.14 | 4.0 | 40 | 60 | 62 | 46 | 1 | B | 2 | 0 | 0 | 2022 | C | 960 | 0.030 |
| (Park 2022) | - | 0 | 0 | -71 | 0 | 0 | 3.72 | 36 | 0.44 | 0 | 1 | 1 | US | 1 | 0.14 | 4.0 | 40 | 60 | 62 | 46 | 1 | B | 2 | 0 | 0 | 2022 | C | 960 | 0.240 |
| (Park 2022) | - | 0 | 0 | -71 | 0 | 0 | 3.72 | 36 | 0.44 | 0 | 1 | 1 | US | 1 | 0.14 | 4.0 | 40 | 60 | 62 | 46 | 1 | B | 2 | 0 | 0 | 2022 | P | 960 | 0.070 |
| (Rim et al. 2022) | S1 | 0 | 0 | -67 | 0 | 0 | 3.72 | 40 | 0.58 | 0 | 0 | 1 | US | 1 | 0.14 | 4.0 | 40 | 60 | 62 | 46 | 1 | B | 2 | 0 | 1 | 2022 | C | 203 | -0.150 |
| (Rim et al. 2022) | S1 | 0 | 0 | -67 | 0 | 0 | 3.72 | 40 | 0.58 | 0 | 0 | 1 | US | 1 | 0.14 | 4.0 | 40 | 60 | 62 | 46 | 1 | B | 2 | 0 | 1 | 2022 | C | 203 | -0.002 |
| (Rim et al. 2022) | S1 | 0 | 0 | -67 | 0 | 0 | 3.72 | 40 | 0.58 | 0 | 0 | 1 | US | 1 | 0.14 | 4.0 | 40 | 60 | 62 | 46 | 1 | B | 2 | 0 | 1 | 2022 | C | 203 | 0.390 |
| (Rim et al. 2022) | S1 | 0 | 0 | -67 | 0 | 0 | 3.72 | 40 | 0.58 | 0 | 0 | 1 | US | 1 | 0.14 | 4.0 | 40 | 60 | 62 | 46 | 1 | B | 2 | 0 | 1 | 2022 | S | 203 | 0.058 |
| (Rim et al. 2022) | S1 | 0 | 0 | -67 | 0 | 0 | 3.72 | 40 | 0.58 | 0 | 0 | 1 | US | 1 | 0.14 | 4.0 | 40 | 60 | 62 | 46 | 1 | B | 2 | 0 | 1 | 2022 | S | 203 | 0.374 |
| (Rim et al. 2022) | S1 | 0 | 0 | -67 | 0 | 0 | 3.72 | 40 | 0.58 | 0 | 0 | 1 | US | 1 | 0.14 | 4.0 | 40 | 60 | 62 | 46 | 1 | B | 2 | 0 | 1 | 2022 | S | 203 | -0.406 |
| (Rim et al. 2022) | S2 | 0 | 0 | -74 | 0 | 0 | 3.72 | 38 | 0.59 | 0 | 0 | 0 | US | 1 | 0.14 | 4.0 | 40 | 60 | 62 | 46 | 1 | B | 2 | 0 | 1 | 2022 | C | 258 | 0.003 |
| (Rim et al. 2022) | S2 | 0 | 0 | -74 | 0 | 0 | 3.72 | 38 | 0.59 | 0 | 0 | 0 | US | 1 | 0.14 | 4.0 | 40 | 60 | 62 | 46 | 1 | B | 2 | 0 | 1 | 2022 | C | 258 | -0.208 |
| (Rim et al. 2022) | S2 | 0 | 0 | -74 | 0 | 0 | 3.72 | 38 | 0.59 | 0 | 0 | 0 | US | 1 | 0.14 | 4.0 | 40 | 60 | 62 | 46 | 1 | B | 2 | 0 | 1 | 2022 | C | 258 | 0.271 |
| (Rim et al. 2022) | S2 | 0 | 0 | -74 | 0 | 0 | 3.72 | 38 | 0.59 | 0 | 0 | 0 | US | 1 | 0.14 | 4.0 | 40 | 60 | 62 | 46 | 1 | B | 2 | 0 | 1 | 2022 | S | 258 | 0.092 |
| (Rim et al. 2022) | S2 | 0 | 0 | -74 | 0 | 0 | 3.72 | 38 | 0.59 | 0 | 0 | 0 | US | 1 | 0.14 | 4.0 | 40 | 60 | 62 | 46 | 1 | B | 2 | 0 | 1 | 2022 | S | 258 | 0.316 |
| (Rim et al. 2022) | S2 | 0 | 0 | -74 | 0 | 0 | 3.72 | 38 | 0.59 | 0 | 0 | 0 | US | 1 | 0.14 | 4.0 | 40 | 60 | 62 | 46 | 1 | B | 2 | 0 | 1 | 2022 | S | 258 | -0.381 |
| (Rim et al. 2022) | S3 | 0 | 0 | -71 | 0 | 0 | 3.72 | 39 | 0.50 | 0 | 0 | 0 | US | 1 | 0.14 | 4.0 | 40 | 60 | 62 | 46 | 1 | B | 2 | 0 | 1 | 2022 | C | 407 | -0.186 |
| (Rim et al. 2022) | S3 | 0 | 0 | -71 | 0 | 0 | 3.72 | 39 | 0.50 | 0 | 0 | 0 | US | 1 | 0.14 | 4.0 | 40 | 60 | 62 | 46 | 1 | B | 2 | 0 | 1 | 2022 | C | 407 | 0.000 |
| (Rim et al. 2022) | S3 | 0 | 0 | -71 | 0 | 0 | 3.72 | 39 | 0.50 | 0 | 0 | 0 | US | 1 | 0.14 | 4.0 | 40 | 60 | 62 | 46 | 1 | B | 2 | 0 | 1 | 2022 | C | 407 | 0.348 |
| (Rim et al. 2022) | S3 | 0 | 0 | -71 | 0 | 0 | 3.72 | 39 | 0.50 | 0 | 0 | 0 | US | 1 | 0.14 | 4.0 | 40 | 60 | 62 | 46 | 1 | B | 2 | 0 | 1 | 2022 | S | 407 | 0.074 |
| (Rim et al. 2022) | S3 | 0 | 0 | -71 | 0 | 0 | 3.72 | 39 | 0.50 | 0 | 0 | 0 | US | 1 | 0.14 | 4.0 | 40 | 60 | 62 | 46 | 1 | B | 2 | 0 | 1 | 2022 | S | 407 | 0.358 |
| (Rim et al. 2022) | S3 | 0 | 0 | -71 | 0 | 0 | 3.72 | 39 | 0.50 | 0 | 0 | 0 | US | 1 | 0.14 | 4.0 | 40 | 60 | 62 | 46 | 1 | B | 2 | 0 | 1 | 2022 | S | 407 | -0.406 |
| (Sauter and Jungblut 2023) | - | 0 | 0 | -71 | 0 | 0 | 3.72 | 36 | 0.50 | 0 | 0 | 0 | Germany | 0 | 0.32 | 3.0 | 35 | 79 | 66 | 65 | 1 | B | 2 | 0 | 1 | 2023 | C | 330 | -0.114 |
| (Sauter and Jungblut 2023) | - | 0 | 0 | -71 | 0 | 0 | 3.72 | 36 | 0.50 | 0 | 0 | 0 | Germany | 0 | 0.32 | 3.0 | 35 | 79 | 66 | 65 | 1 | B | 2 | 0 | 1 | 2023 | C | 330 | -0.228 |
| (Sauter and Jungblut 2023) | - | 1 | 0 | -71 | 0 | 0 | 3.72 | 36 | 0.50 | 0 | 0 | 0 | Germany | 0 | 0.32 | 3.0 | 35 | 79 | 66 | 65 | 1 | B | 2 | 0 | 1 | 2023 | C | 330 | 0.085 |
| (Sauter and Jungblut 2023) | - | 1 | 0 | -71 | 0 | 0 | 3.72 | 36 | 0.50 | 0 | 0 | 0 | Germany | 0 | 0.32 | 3.0 | 35 | 79 | 66 | 65 | 1 | B | 2 | 0 | 1 | 2023 | C | 330 | 0.067 |
| (Sauter and Jungblut 2023) | - | 0 | 0 | -71 | 0 | 0 | 3.72 | 36 | 0.50 | 0 | 0 | 0 | Germany | 0 | 0.32 | 3.0 | 35 | 79 | 66 | 65 | 1 | B | 2 | 0 | 1 | 2023 | C | 330 | -0.052 |
| (Sauter and Jungblut 2023) | - | 0 | 0 | -71 | 0 | 0 | 3.72 | 36 | 0.50 | 0 | 0 | 0 | Germany | 0 | 0.32 | 3.0 | 35 | 79 | 66 | 65 | 1 | B | 2 | 0 | 1 | 2023 | C | 330 | 0.363 |
| (Sauter and Jungblut 2023) | - | 0 | 0 | -71 | 0 | 0 | 3.72 | 36 | 0.50 | 0 | 0 | 0 | Germany | 0 | 0.32 | 3.0 | 35 | 79 | 66 | 65 | 1 | B | 2 | 0 | 1 | 2023 | C | 330 | 0.051 |
| (Sauter and Jungblut 2023) | - | 0 | 0 | -71 | 0 | 0 | 3.72 | 36 | 0.50 | 0 | 0 | 0 | Germany | 0 | 0.32 | 3.0 | 35 | 79 | 66 | 65 | 1 | B | 2 | 0 | 1 | 2023 | C | 330 | 0.400 |
| (Sauter and Jungblut 2023) | - | 0 | 1 | -71 | 0 | 0 | 3.72 | 36 | 0.50 | 0 | 0 | 0 | Germany | 0 | 0.32 | 3.0 | 35 | 79 | 66 | 65 | 1 | B | 2 | 0 | 1 | 2023 | C | 330 | 0.006 |
| (Sauter and Jungblut 2023) | - | 0 | 1 | -71 | 0 | 0 | 3.72 | 36 | 0.50 | 0 | 0 | 0 | Germany | 0 | 0.32 | 3.0 | 35 | 79 | 66 | 65 | 1 | B | 2 | 0 | 1 | 2023 | C | 330 | 0.033 |
| (Sauter and Jungblut 2023) | - | 1 | 0 | -71 | 0 | 0 | 3.72 | 36 | 0.50 | 0 | 0 | 0 | Germany | 0 | 0.32 | 3.0 | 35 | 79 | 66 | 65 | 1 | B | 2 | 0 | 1 | 2023 | E | 330 | 0.084 |
| (Sauter and Jungblut 2023) | - | 0 | 1 | -71 | 0 | 0 | 3.72 | 36 | 0.50 | 0 | 0 | 0 | Germany | 0 | 0.32 | 3.0 | 35 | 79 | 66 | 65 | 1 | B | 2 | 0 | 1 | 2023 | E | 330 | -0.002 |
| (Song and Choi 2023) | - | 1 | 0 | -71 | 1 | 0 | 3.77 | 44 | 0.47 | 1 | 0 | 1 | US | 1 | 0.14 | 4.0 | 40 | 60 | 62 | 46 | 1 | B | 2 | 0 | 0 | 2023 | C | 530 | 0.520 |
| (Song and Choi 2023) | - | 1 | 0 | -71 | 1 | 0 | 3.77 | 44 | 0.47 | 1 | 0 | 1 | US | 1 | 0.14 | 4.0 | 40 | 60 | 62 | 46 | 1 | B | 2 | 0 | 0 | 2023 | C | 530 | 0.920 |
| (Song and Choi 2023) | - | 1 | 0 | -71 | 1 | 0 | 3.77 | 44 | 0.47 | 1 | 0 | 1 | US | 1 | 0.14 | 4.0 | 40 | 60 | 62 | 46 | 1 | B | 2 | 0 | 0 | 2023 | S | 530 | 0.680 |
| (Song and Choi 2023) | - | 1 | 0 | -71 | 1 | 0 | 3.77 | 44 | 0.47 | 1 | 0 | 1 | US | 1 | 0.14 | 4.0 | 40 | 60 | 62 | 46 | 1 | B | 2 | 0 | 0 | 2023 | S | 530 | 0.760 |
| (Troy et al. 2023) | - | 1 | 0 | -87 | 0 | 0 | 3.72 | 47 | 0.50 | 0 | 0 | 1 | US | 1 | 0.14 | 4.0 | 40 | 60 | 62 | 46 | 1 | B | 2 | 0 | 1 | 2023 | P | 551 | 0.099 |
| (Troy et al. 2023) | - | 1 | 0 | -87 | 0 | 0 | 3.72 | 47 | 0.50 | 0 | 0 | 1 | US | 1 | 0.14 | 4.0 | 40 | 60 | 62 | 46 | 1 | B | 2 | 0 | 1 | 2023 | P | 551 | -0.099 |
| (Xu et al. 2021) | - | 1 | 0 | -71 | 0 | 0 | 3.72 | 47 | 0.53 | 0 | 0 | 1 | US | 1 | 0.14 | 4.0 | 40 | 60 | 62 | 46 | 1 | B | 2 | 0 | 1 | 2021 | C | 296 | -0.103 |
| (Xu et al. 2021) | - | 1 | 0 | -71 | 0 | 0 | 3.72 | 47 | 0.53 | 0 | 0 | 1 | US | 1 | 0.14 | 4.0 | 40 | 60 | 62 | 46 | 1 | B | 2 | 0 | 1 | 2021 | P | 296 | -0.005 |
| (Xu et al. 2021) | - | 1 | 0 | -71 | 0 | 0 | 3.72 | 47 | 0.53 | 0 | 0 | 1 | US | 1 | 0.14 | 4.0 | 40 | 60 | 62 | 46 | 1 | B | 2 | 0 | 1 | 2021 | P | 296 | -0.282 |
| (Zhang and Zhou 2023) | S1 | 0 | 0 | -63 | 0 | 0 | 3.72 | 37 | 0.59 | 0 | 0 | 1 | US | 1 | 0.14 | 4.0 | 40 | 60 | 62 | 46 | 1 | B | 2 | 0 | 1 | 2023 | C | 183 | -0.139 |
| (Zhang and Zhou 2023) | S1 | 0 | 0 | -63 | 0 | 0 | 3.72 | 37 | 0.59 | 0 | 0 | 1 | US | 1 | 0.14 | 4.0 | 40 | 60 | 62 | 46 | 1 | B | 2 | 0 | 1 | 2023 | C | 183 | -0.171 |
| (Zhang and Zhou 2023) | S1 | 0 | 0 | -63 | 0 | 0 | 3.72 | 37 | 0.59 | 0 | 0 | 1 | US | 1 | 0.14 | 4.0 | 40 | 60 | 62 | 46 | 1 | B | 2 | 0 | 1 | 2023 | C | 183 | -0.108 |
| (Zhang and Zhou 2023) | S1 | 0 | 0 | -63 | 0 | 0 | 3.72 | 37 | 0.59 | 0 | 0 | 1 | US | 1 | 0.14 | 4.0 | 40 | 60 | 62 | 46 | 1 | B | 2 | 0 | 1 | 2023 | C | 183 | -0.007 |
| (Zhang and Zhou 2023) | S2 | 1 | 0 | -74 | 0 | 0 | 3.72 | 38 | 0.62 | 0 | 1 | 1 | US | 1 | 0.14 | 4.0 | 40 | 60 | 62 | 46 | 1 | B | 2 | 0 | 1 | 2023 | C | 191 | -0.296 |
| (Zhang and Borden 2022) | - | 0 | 0 | -71 | 1 | 0 | 2.53 | 37 | 0.43 | 0 | 0 | 1 | US | 1 | 0.14 | 4.0 | 40 | 60 | 62 | 46 | 1 | B | 2 | 0 | 0 | 2022 | C | 590 | 0.578 |
| (Zhang and Borden 2022) | - | 0 | 0 | -71 | 1 | 0 | 2.53 | 37 | 0.43 | 0 | 0 | 1 | US | 1 | 0.14 | 4.0 | 40 | 60 | 62 | 46 | 1 | B | 2 | 0 | 0 | 2022 | P | 590 | 0.136 |
| (Zhang and Borden 2022) | - | 0 | 0 | -71 | 1 | 0 | 2.53 | 37 | 0.43 | 0 | 0 | 1 | US | 1 | 0.14 | 4.0 | 40 | 60 | 62 | 46 | 1 | B | 2 | 0 | 0 | 2022 | S | 590 | 0.537 |
| (Park and Jiang 2020) | - | 0 | 0 | -71 | 0 | 0 | 3.72 | 36 | 0.44 | 1 | 1 | 1 | US | 1 | 0.14 | 4.0 | 40 | 60 | 62 | 46 | 1 | C | 1 | 0 | 0 | 2020 | C | 960 | 0.180 |
| (Park and Jiang 2020) | - | 0 | 0 | -71 | 0 | 0 | 3.72 | 36 | 0.44 | 1 | 1 | 1 | US | 1 | 0.14 | 4.0 | 40 | 60 | 62 | 46 | 1 | C | 1 | 0 | 0 | 2020 | P | 960 | 0.210 |
| (Wang et al. 2023) | - | 1 | 0 | -83 | 1 | 0 | 3.42 | 44 | 0.44 | 0 | 1 | 1 | US | 1 | 0.14 | 4.0 | 40 | 60 | 62 | 46 | 1 | C | 1 | 0 | 0 | 2023 | C | 375 | 0.324 |
| (Wang et al. 2023) | - | 1 | 0 | -83 | 1 | 0 | 3.42 | 44 | 0.44 | 0 | 1 | 1 | US | 1 | 0.14 | 4.0 | 40 | 60 | 62 | 46 | 1 | C | 1 | 0 | 0 | 2023 | C | 375 | 0.016 |
| (You et al. 2023) | - | 1 | 0 | -71 | 0 | 0 | 3.72 | 37 | 0.45 | 1 | 1 | 1 | US | 1 | 0.14 | 4.0 | 40 | 60 | 62 | 46 | 1 | C | 1 | 0 | 0 | 2023 | P | 913 | 0.533 |
| (You et al. 2023) | - | 1 | 0 | -71 | 0 | 0 | 3.72 | 37 | 0.45 | 1 | 1 | 1 | US | 1 | 0.14 | 4.0 | 40 | 60 | 62 | 46 | 1 | C | 1 | 0 | 0 | 2023 | P | 913 | 0.277 |
| (Arkema 2022) | S2 | 1 | 0 | -68 | 0 | 0 | 3.72 | 25 | 0.51 | 0 | 0 | 0 | The Netherlands | 0 | 0.02 | 3.0 | 38 | 100 | 14 | 53 | 0 | 0 | 0 | 0 | 1 | 2022 | C | 109 | 0.374 |
| (Arkema 2022) | S2 | 1 | 0 | -68 | 0 | 0 | 3.72 | 25 | 0.51 | 0 | 0 | 0 | The Netherlands | 0 | 0.02 | 3.0 | 38 | 100 | 14 | 53 | 0 | 0 | 0 | 0 | 1 | 2022 | C | 109 | 0.635 |
| (Arkema 2022) | S2 | 1 | 0 | -68 | 0 | 0 | 3.72 | 25 | 0.51 | 0 | 0 | 0 | The Netherlands | 0 | 0.02 | 3.0 | 38 | 100 | 14 | 53 | 0 | 0 | 0 | 0 | 1 | 2022 | P | 109 | 0.351 |
| (Arkema 2022) | S2 | 1 | 0 | -68 | 0 | 0 | 3.72 | 25 | 0.51 | 0 | 0 | 0 | The Netherlands | 0 | 0.02 | 3.0 | 38 | 100 | 14 | 53 | 0 | 0 | 0 | 0 | 1 | 2022 | P | 109 | 0.608 |
| (Nam et al. 2023) | S3A | 1 | 0 | -83 | 0 | 0 | 3.72 | 34 | 0.50 | 0 | 0 | 0 | US | 1 | 0.14 | 4.0 | 40 | 60 | 62 | 46 | 0 | 0 | 0 | 0 | 1 | 2023 | C | 374 | 0.582 |
| (Nam et al. 2023) | S3B | 1 | 0 | -83 | 0 | 0 | 3.72 | 36 | 0.59 | 0 | 0 | 0 | US | 1 | 0.14 | 4.0 | 40 | 60 | 62 | 46 | 0 | 0 | 0 | 0 | 1 | 2023 | P | 600 | -0.131 |
| (Nam et al. 2023) | S4 | 1 | 0 | -83 | 0 | 0 | 3.72 | 36 | 0.46 | 0 | 0 | 0 | US | 1 | 0.14 | 4.0 | 40 | 60 | 62 | 46 | 0 | 0 | 0 | 0 | 1 | 2023 | C | 500 | 0.648 |
| (Bondi et al. 2023) | S1A | 0 | 0 | -80 | 1 | 0 | 3.76 | 33 | 0.66 | 0 | 0 | 0 | US | 1 | 0.14 | 4.0 | 40 | 60 | 62 | 46 | 0 | 0 | 0 | 0 | 1 | 2023 | C | 1153 | -0.300 |
| (Bondi et al. 2023) | S1A | 0 | 0 | -80 | 1 | 0 | 3.76 | 33 | 0.66 | 0 | 0 | 0 | US | 1 | 0.14 | 4.0 | 40 | 60 | 62 | 46 | 0 | 0 | 0 | 0 | 1 | 2023 | C | 1153 | -0.137 |
| (Bondi et al. 2023) | S1A | 0 | 0 | -80 | 1 | 0 | 3.76 | 33 | 0.66 | 0 | 0 | 0 | US | 1 | 0.14 | 4.0 | 40 | 60 | 62 | 46 | 0 | 0 | 0 | 0 | 1 | 2023 | C | 1153 | 0.144 |
| (Bondi et al. 2023) | S1A | 0 | 0 | -80 | 1 | 0 | 3.76 | 33 | 0.66 | 0 | 0 | 0 | US | 1 | 0.14 | 4.0 | 40 | 60 | 62 | 46 | 0 | 0 | 0 | 0 | 1 | 2023 | C | 1153 | 0.155 |
| (Bondi et al. 2023) | S1A | 0 | 0 | -80 | 1 | 0 | 3.76 | 33 | 0.66 | 0 | 0 | 0 | US | 1 | 0.14 | 4.0 | 40 | 60 | 62 | 46 | 0 | 0 | 0 | 0 | 1 | 2023 | C | 1153 | -0.124 |
| (Bondi et al. 2023) | S1A | 0 | 0 | -80 | 1 | 0 | 3.76 | 33 | 0.66 | 0 | 0 | 0 | US | 1 | 0.14 | 4.0 | 40 | 60 | 62 | 46 | 0 | 0 | 0 | 0 | 1 | 2023 | C | 1153 | -0.187 |
| (Bondi et al. 2023) | S1A | 1 | 0 | -80 | 1 | 0 | 3.76 | 33 | 0.66 | 0 | 0 | 0 | US | 1 | 0.14 | 4.0 | 40 | 60 | 62 | 46 | 0 | 0 | 0 | 0 | 1 | 2023 | W | 1153 | -0.131 |
| (Bondi et al. 2023) | S1A | 1 | 0 | -80 | 1 | 0 | 3.76 | 33 | 0.66 | 0 | 0 | 0 | US | 1 | 0.14 | 4.0 | 40 | 60 | 62 | 46 | 0 | 0 | 0 | 0 | 1 | 2023 | W | 1153 | 0.122 |
| (Bondi et al. 2023) | S1A | 0 | 1 | -80 | 1 | 0 | 3.76 | 33 | 0.66 | 0 | 0 | 0 | US | 1 | 0.14 | 4.0 | 40 | 60 | 62 | 46 | 0 | 0 | 0 | 0 | 1 | 2023 | W | 1153 | -0.274 |
| (Bondi et al. 2023) | S1A | 0 | 1 | -80 | 1 | 0 | 3.76 | 33 | 0.66 | 0 | 0 | 0 | US | 1 | 0.14 | 4.0 | 40 | 60 | 62 | 46 | 0 | 0 | 0 | 0 | 1 | 2023 | W | 1153 | 0.168 |
| (Bondi et al. 2023) | S1A | 0 | 1 | -80 | 1 | 0 | 3.76 | 33 | 0.66 | 0 | 0 | 0 | US | 1 | 0.14 | 4.0 | 40 | 60 | 62 | 46 | 0 | 0 | 0 | 0 | 1 | 2023 | W | 1153 | -0.108 |
| (Bondi et al. 2023) | S1A | 1 | 0 | -80 | 1 | 0 | 3.76 | 33 | 0.66 | 0 | 0 | 0 | US | 1 | 0.14 | 4.0 | 40 | 60 | 62 | 46 | 0 | 0 | 0 | 0 | 1 | 2023 | W | 1153 | -0.155 |
| (Bondi et al. 2023) | S1B | 0 | 1 | -80 | 0 | 0 | 3.72 | 36 | 0.50 | 0 | 0 | 0 | US | 1 | 0.14 | 4.0 | 40 | 60 | 62 | 46 | 0 | 0 | 0 | 0 | 1 | 2023 | C | 4612 | -0.119 |
| (Bondi et al. 2023) | S1B | 1 | 0 | -80 | 0 | 0 | 3.72 | 36 | 0.50 | 0 | 0 | 0 | US | 1 | 0.14 | 4.0 | 40 | 60 | 62 | 46 | 0 | 0 | 0 | 0 | 1 | 2023 | C | 4612 | -0.146 |
| (Bondi et al. 2023) | S1B | 1 | 0 | -80 | 0 | 0 | 3.72 | 36 | 0.50 | 0 | 0 | 0 | US | 1 | 0.14 | 4.0 | 40 | 60 | 62 | 46 | 0 | 0 | 0 | 0 | 1 | 2023 | C | 4612 | -0.068 |
| (Bondi et al. 2023) | S1B | 1 | 0 | -80 | 0 | 0 | 3.72 | 36 | 0.50 | 0 | 0 | 0 | US | 1 | 0.14 | 4.0 | 40 | 60 | 62 | 46 | 0 | 0 | 0 | 0 | 1 | 2023 | C | 4612 | 0.164 |
| (Bondi et al. 2023) | S1B | 0 | 1 | -80 | 0 | 0 | 3.72 | 36 | 0.50 | 0 | 0 | 0 | US | 1 | 0.14 | 4.0 | 40 | 60 | 62 | 46 | 0 | 0 | 0 | 0 | 1 | 2023 | C | 4612 | -0.285 |
| (Bondi et al. 2023) | S1B | 0 | 1 | -80 | 0 | 0 | 3.72 | 36 | 0.50 | 0 | 0 | 0 | US | 1 | 0.14 | 4.0 | 40 | 60 | 62 | 46 | 0 | 0 | 0 | 0 | 1 | 2023 | C | 4612 | 0.142 |
| (Bondi et al. 2023) | S1B | 0 | 1 | -80 | 0 | 0 | 3.72 | 36 | 0.50 | 0 | 0 | 0 | US | 1 | 0.14 | 4.0 | 40 | 60 | 62 | 46 | 0 | 0 | 0 | 0 | 1 | 2023 | W | 4612 | -0.093 |
| (Bondi et al. 2023) | S1B | 1 | 0 | -80 | 0 | 0 | 3.72 | 36 | 0.50 | 0 | 0 | 0 | US | 1 | 0.14 | 4.0 | 40 | 60 | 62 | 46 | 0 | 0 | 0 | 0 | 1 | 2023 | W | 4612 | -0.142 |
| (Bondi et al. 2023) | S1B | 1 | 0 | -80 | 0 | 0 | 3.72 | 36 | 0.50 | 0 | 0 | 0 | US | 1 | 0.14 | 4.0 | 40 | 60 | 62 | 46 | 0 | 0 | 0 | 0 | 1 | 2023 | W | 4612 | -0.104 |
| (Bondi et al. 2023) | S1B | 1 | 0 | -80 | 0 | 0 | 3.72 | 36 | 0.50 | 0 | 0 | 0 | US | 1 | 0.14 | 4.0 | 40 | 60 | 62 | 46 | 0 | 0 | 0 | 0 | 1 | 2023 | W | 4612 | 0.147 |
| (Bondi et al. 2023) | S1B | 0 | 1 | -80 | 0 | 0 | 3.72 | 36 | 0.50 | 0 | 0 | 0 | US | 1 | 0.14 | 4.0 | 40 | 60 | 62 | 46 | 0 | 0 | 0 | 0 | 1 | 2023 | W | 4612 | -0.275 |
| (Bondi et al. 2023) | S1B | 0 | 1 | -80 | 0 | 0 | 3.72 | 36 | 0.50 | 0 | 0 | 0 | US | 1 | 0.14 | 4.0 | 40 | 60 | 62 | 46 | 0 | 0 | 0 | 0 | 1 | 2023 | W | 4612 | 0.159 |
| (Bondi et al. 2023) | S2A | 1 | 0 | -80 | 1 | 0 | 3.76 | 35 | 0.67 | 0 | 0 | 0 | US | 1 | 0.14 | 4.0 | 40 | 60 | 62 | 46 | 0 | 0 | 0 | 0 | 1 | 2023 | C | 1754 | 0.081 |
| (Bondi et al. 2023) | S2A | 1 | 0 | -80 | 1 | 0 | 3.76 | 35 | 0.67 | 0 | 0 | 0 | US | 1 | 0.14 | 4.0 | 40 | 60 | 62 | 46 | 0 | 0 | 0 | 0 | 1 | 2023 | C | 1754 | 0.236 |
| (Bondi et al. 2023) | S2A | 0 | 1 | -80 | 1 | 0 | 3.76 | 35 | 0.67 | 0 | 0 | 0 | US | 1 | 0.14 | 4.0 | 40 | 60 | 62 | 46 | 0 | 0 | 0 | 0 | 1 | 2023 | C | 1754 | -0.244 |
| (Bondi et al. 2023) | S2A | 0 | 1 | -80 | 1 | 0 | 3.76 | 35 | 0.67 | 0 | 0 | 0 | US | 1 | 0.14 | 4.0 | 40 | 60 | 62 | 46 | 0 | 0 | 0 | 0 | 1 | 2023 | C | 1754 | -0.195 |
| (Bondi et al. 2023) | S2A | 1 | 0 | -80 | 1 | 0 | 3.76 | 35 | 0.67 | 0 | 0 | 0 | US | 1 | 0.14 | 4.0 | 40 | 60 | 62 | 46 | 0 | 0 | 0 | 0 | 1 | 2023 | W | 1754 | 0.025 |
| (Bondi et al. 2023) | S2A | 1 | 0 | -80 | 1 | 0 | 3.76 | 35 | 0.67 | 0 | 0 | 0 | US | 1 | 0.14 | 4.0 | 40 | 60 | 62 | 46 | 0 | 0 | 0 | 0 | 1 | 2023 | W | 1754 | 0.230 |
| (Bondi et al. 2023) | S2A | 0 | 1 | -80 | 1 | 0 | 3.76 | 35 | 0.67 | 0 | 0 | 0 | US | 1 | 0.14 | 4.0 | 40 | 60 | 62 | 46 | 0 | 0 | 0 | 0 | 1 | 2023 | W | 1754 | -0.216 |
| (Bondi et al. 2023) | S2A | 0 | 1 | -80 | 1 | 0 | 3.76 | 35 | 0.67 | 0 | 0 | 0 | US | 1 | 0.14 | 4.0 | 40 | 60 | 62 | 46 | 0 | 0 | 0 | 0 | 1 | 2023 | W | 1754 | -0.141 |
| (Bondi et al. 2023) | S2B | 0 | 1 | -80 | 0 | 0 | 3.72 | 36 | 0.50 | 0 | 0 | 0 | US | 1 | 0.14 | 4.0 | 40 | 60 | 62 | 46 | 0 | 0 | 0 | 0 | 1 | 2023 | C | 7016 | -0.166 |
| (Bondi et al. 2023) | S2B | 1 | 0 | -80 | 0 | 0 | 3.72 | 36 | 0.50 | 0 | 0 | 0 | US | 1 | 0.14 | 4.0 | 40 | 60 | 62 | 46 | 0 | 0 | 0 | 0 | 1 | 2023 | C | 7016 | 0.093 |
| (Bondi et al. 2023) | S2B | 1 | 0 | -80 | 0 | 0 | 3.72 | 36 | 0.50 | 0 | 0 | 0 | US | 1 | 0.14 | 4.0 | 40 | 60 | 62 | 46 | 0 | 0 | 0 | 0 | 1 | 2023 | C | 7016 | 0.210 |
| (Bondi et al. 2023) | S2B | 0 | 1 | -80 | 0 | 0 | 3.72 | 36 | 0.50 | 0 | 0 | 0 | US | 1 | 0.14 | 4.0 | 40 | 60 | 62 | 46 | 0 | 0 | 0 | 0 | 1 | 2023 | C | 7016 | -0.185 |
| (Bondi et al. 2023) | S2B | 0 | 1 | -80 | 0 | 0 | 3.72 | 36 | 0.50 | 0 | 0 | 0 | US | 1 | 0.14 | 4.0 | 40 | 60 | 62 | 46 | 0 | 0 | 0 | 0 | 1 | 2023 | W | 7016 | -0.151 |
| (Bondi et al. 2023) | S2B | 1 | 0 | -80 | 0 | 0 | 3.72 | 36 | 0.50 | 0 | 0 | 0 | US | 1 | 0.14 | 4.0 | 40 | 60 | 62 | 46 | 0 | 0 | 0 | 0 | 1 | 2023 | W | 7016 | 0.035 |
| (Bondi et al. 2023) | S2B | 1 | 0 | -80 | 0 | 0 | 3.72 | 36 | 0.50 | 0 | 0 | 0 | US | 1 | 0.14 | 4.0 | 40 | 60 | 62 | 46 | 0 | 0 | 0 | 0 | 1 | 2023 | W | 7016 | 0.177 |
| (Bondi et al. 2023) | S2B | 0 | 1 | -80 | 0 | 0 | 3.72 | 36 | 0.50 | 0 | 0 | 0 | US | 1 | 0.14 | 4.0 | 40 | 60 | 62 | 46 | 0 | 0 | 0 | 0 | 1 | 2023 | W | 7016 | -0.192 |
| (Burggraaf and Larsson 2019) | - | 1 | 0 | -68 | 0 | 1 | 4.27 | 25 | 0.38 | 0 | 0 | 0 | Sweden | 0 | 0.42 | 4.0 | 31 | 87 | 5 | 29 | 0 | 0 | 0 | 0 | 0 | 2019 | C | 115 | 0.363 |
| (Burggraaf and Larsson 2019) | - | 1 | 0 | -68 | 0 | 1 | 4.27 | 25 | 0.38 | 0 | 0 | 0 | Sweden | 0 | 0.42 | 4.0 | 31 | 87 | 5 | 29 | 0 | 0 | 0 | 0 | 0 | 2019 | C | 115 | 0.397 |
| (Burggraaf and Larsson 2019) | - | 1 | 0 | -68 | 0 | 1 | 4.27 | 25 | 0.38 | 0 | 0 | 0 | Sweden | 0 | 0.42 | 4.0 | 31 | 87 | 5 | 29 | 0 | 0 | 0 | 0 | 0 | 2019 | C | 115 | 0.546 |
| (Burggraaf and Larsson 2019) | - | 1 | 0 | -68 | 0 | 1 | 4.27 | 25 | 0.38 | 0 | 0 | 0 | Sweden | 0 | 0.42 | 4.0 | 31 | 87 | 5 | 29 | 0 | 0 | 0 | 0 | 0 | 2019 | C | 115 | 0.581 |
| (Burggraaf and Larsson 2019) | - | 1 | 0 | -68 | 0 | 1 | 4.27 | 25 | 0.38 | 0 | 0 | 0 | Sweden | 0 | 0.42 | 4.0 | 31 | 87 | 5 | 29 | 0 | 0 | 0 | 0 | 0 | 2019 | C | 115 | 0.553 |
| (Burggraaf and Larsson 2019) | - | 1 | 0 | -68 | 0 | 1 | 4.27 | 25 | 0.38 | 0 | 0 | 0 | Sweden | 0 | 0.42 | 4.0 | 31 | 87 | 5 | 29 | 0 | 0 | 0 | 0 | 0 | 2019 | P | 115 | 0.599 |
| (Burggraaf and Larsson 2019) | - | 1 | 0 | -68 | 0 | 1 | 4.27 | 25 | 0.38 | 0 | 0 | 0 | Sweden | 0 | 0.42 | 4.0 | 31 | 87 | 5 | 29 | 0 | 0 | 0 | 0 | 0 | 2019 | E | 115 | 0.517 |
| (Dodd and Supa 2013) | - | 0 | 0 | -63 | 0 | 1 | 4.17 | 24 | 0.30 | 0 | 0 | 1 | US | 1 | 0.14 | 4.0 | 40 | 60 | 62 | 46 | 0 | 0 | 0 | 0 | 1 | 2013 | P | 519 | -0.133 |
| (Edelblum 2022) | S1 | 1 | 0 | -83 | 0 | 0 | 3.72 | 36 | 0.48 | 0 | 0 | 0 | US | 1 | 0.14 | 4.0 | 40 | 60 | 62 | 46 | 0 | 0 | 0 | 0 | 1 | 2022 | S | 304 | -0.133 |
| (Edelblum 2022) | S2 | 1 | 0 | -68 | 0 | 0 | 3.72 | 30 | 0.41 | 0 | 0 | 0 | US | 1 | 0.14 | 4.0 | 40 | 60 | 62 | 46 | 0 | 0 | 0 | 0 | 1 | 2022 | S | 169 | -0.144 |
| (Edelblum 2022) | S3 | 1 | 0 | -83 | 0 | 0 | 3.72 | 36 | 0.59 | 0 | 0 | 0 | US | 1 | 0.14 | 4.0 | 40 | 60 | 62 | 46 | 0 | 0 | 0 | 0 | 1 | 2022 | S | 377 | -0.013 |
| (Enete and Sturr 2022) | - | 0 | 1 | -67 | 1 | 0 | 3.87 | 36 | 0.60 | 1 | 0 | 1 | US | 1 | 0.14 | 4.0 | 40 | 60 | 62 | 46 | 0 | 0 | 0 | 0 | 1 | 2022 | P | 367 | 0.183 |
| (Enete and Sturr 2022) | - | 0 | 1 | -67 | 1 | 0 | 3.87 | 36 | 0.60 | 1 | 0 | 1 | US | 1 | 0.14 | 4.0 | 40 | 60 | 62 | 46 | 0 | 0 | 0 | 0 | 1 | 2022 | P | 183 | 0.079 |
| (Enete and Sturr 2022) | - | 0 | 1 | -67 | 1 | 0 | 3.87 | 36 | 0.60 | 1 | 0 | 1 | US | 1 | 0.14 | 4.0 | 40 | 60 | 62 | 46 | 0 | 0 | 0 | 0 | 1 | 2022 | P | 183 | -0.486 |
| (Enete and Sturr 2022) | - | 1 | 0 | -50 | 1 | 0 | 3.87 | 36 | 0.60 | 1 | 0 | 1 | US | 1 | 0.14 | 4.0 | 40 | 60 | 62 | 46 | 0 | 0 | 0 | 0 | 1 | 2022 | P | 382 | 0.143 |
| (Enete and Sturr 2022) | - | 1 | 0 | -50 | 1 | 0 | 3.87 | 36 | 0.60 | 1 | 0 | 1 | US | 1 | 0.14 | 4.0 | 40 | 60 | 62 | 46 | 0 | 0 | 0 | 0 | 1 | 2022 | P | 191 | -0.126 |
| (Enete and Sturr 2022) | - | 0 | 1 | -50 | 1 | 0 | 3.87 | 36 | 0.60 | 1 | 0 | 1 | US | 1 | 0.14 | 4.0 | 40 | 60 | 62 | 46 | 0 | 0 | 0 | 0 | 1 | 2022 | P | 182 | -0.139 |
| (Enete and Sturr 2022) | - | 0 | 1 | -50 | 1 | 0 | 3.87 | 36 | 0.60 | 1 | 0 | 1 | US | 1 | 0.14 | 4.0 | 40 | 60 | 62 | 46 | 0 | 0 | 0 | 0 | 1 | 2022 | P | 363 | -0.162 |
| (Enete and Sturr 2022) | - | 1 | 0 | -50 | 1 | 0 | 3.87 | 36 | 0.60 | 1 | 0 | 1 | US | 1 | 0.14 | 4.0 | 40 | 60 | 62 | 46 | 0 | 0 | 0 | 0 | 1 | 2022 | P | 191 | -0.492 |
| (Enete and Sturr 2022) | - | 0 | 1 | -50 | 1 | 0 | 3.87 | 36 | 0.60 | 1 | 0 | 1 | US | 1 | 0.14 | 4.0 | 40 | 60 | 62 | 46 | 0 | 0 | 0 | 0 | 1 | 2022 | P | 182 | -0.552 |
| (Goncalves 2021) | - | 0 | 0 | -71 | 0 | 0 | 3.72 | 26 | 0.57 | 0 | 0 | 0 | Portugal | 0 | -0.34 | 3.3 | 53 | 59 | 31 | 99 | 0 | 0 | 0 | 0 | 1 | 2021 | C | 216 | 0.029 |
| (Goncalves 2021) | - | 0 | 0 | -71 | 0 | 0 | 3.72 | 26 | 0.57 | 0 | 0 | 0 | Portugal | 0 | -0.34 | 3.3 | 53 | 59 | 31 | 99 | 0 | 0 | 0 | 0 | 1 | 2021 | C | 216 | -0.083 |
| (Goncalves 2021) | - | 0 | 0 | -71 | 0 | 0 | 3.72 | 26 | 0.57 | 0 | 0 | 0 | Portugal | 0 | -0.34 | 3.3 | 53 | 59 | 31 | 99 | 0 | 0 | 0 | 0 | 1 | 2021 | C | 216 | -0.029 |
| (Goncalves 2021) | - | 0 | 0 | -71 | 0 | 0 | 3.72 | 26 | 0.57 | 0 | 0 | 0 | Portugal | 0 | -0.34 | 3.3 | 53 | 59 | 31 | 99 | 0 | 0 | 0 | 0 | 1 | 2021 | C | 216 | 0.133 |
| (Goncalves 2021) | - | 0 | 0 | -71 | 0 | 0 | 3.72 | 26 | 0.57 | 0 | 0 | 0 | Portugal | 0 | -0.34 | 3.3 | 53 | 59 | 31 | 99 | 0 | 0 | 0 | 0 | 1 | 2021 | C | 216 | 0.158 |
| (Goncalves 2021) | - | 0 | 0 | -71 | 0 | 0 | 3.72 | 26 | 0.57 | 0 | 0 | 0 | Portugal | 0 | -0.34 | 3.3 | 53 | 59 | 31 | 99 | 0 | 0 | 0 | 0 | 1 | 2021 | C | 216 | -0.084 |
| (Heffron 2019) | - | 1 | 0 | -83 | 1 | 0 | 3.23 | 36 | 0.47 | 0 | 0 | 1 | US | 1 | 0.14 | 4.0 | 40 | 60 | 62 | 46 | 0 | 0 | 0 | 0 | 1 | 2019 | P | 171 | 0.248 |
| (Heffron 2019) | - | 1 | 0 | -83 | 1 | 0 | 3.23 | 36 | 0.47 | 0 | 0 | 1 | US | 1 | 0.14 | 4.0 | 40 | 60 | 62 | 46 | 0 | 0 | 0 | 0 | 1 | 2019 | P | 171 | 0.233 |
| (Jin et al. 2022) | - | 0 | 0 | -71 | 0 | 0 | 3.72 | 28 | 0.50 | 0 | 0 | 1 | US | 1 | 0.14 | 4.0 | 40 | 60 | 62 | 46 | 1 | 0 | 0 | 0 | 0 | 2022 | C | 373 | 0.750 |
| (Jin et al. 2022) | - | 0 | 0 | -71 | 0 | 0 | 3.72 | 28 | 0.50 | 0 | 0 | 1 | US | 1 | 0.14 | 4.0 | 40 | 60 | 62 | 46 | 1 | 0 | 0 | 0 | 0 | 2022 | C | 373 | 0.766 |
| (Jin et al. 2022) | - | 0 | 0 | -71 | 0 | 0 | 3.72 | 28 | 0.50 | 0 | 0 | 1 | US | 1 | 0.14 | 4.0 | 40 | 60 | 62 | 46 | 1 | 0 | 0 | 0 | 0 | 2022 | S | 373 | 0.670 |
| (Jin et al. 2022) | - | 0 | 0 | -71 | 0 | 0 | 3.72 | 28 | 0.50 | 0 | 0 | 1 | US | 1 | 0.14 | 4.0 | 40 | 60 | 62 | 46 | 1 | 0 | 0 | 0 | 0 | 2022 | S | 373 | 0.799 |
| (Jin et al. 2022) | - | 0 | 0 | -71 | 0 | 0 | 3.72 | 28 | 0.50 | 0 | 0 | 1 | US | 1 | 0.14 | 4.0 | 40 | 60 | 62 | 46 | 1 | 0 | 0 | 0 | 0 | 2022 | W | 373 | 0.660 |
| (Jin et al. 2022) | - | 0 | 0 | -71 | 0 | 0 | 3.72 | 28 | 0.50 | 0 | 0 | 1 | US | 1 | 0.14 | 4.0 | 40 | 60 | 62 | 46 | 1 | 0 | 0 | 0 | 0 | 2022 | W | 373 | 0.731 |
| (Karikari 2023) | E2 – S1A | 1 | 0 | -83 | 1 | 0 | 2.52 | 39 | 0.34 | 1 | 1 | 1 | US | 1 | 0.14 | 4.0 | 40 | 60 | 62 | 46 | 0 | 0 | 0 | 0 | 1 | 2023 | C | 170 | 0.383 |
| (Karikari 2023) | E2 – S1A | 1 | 0 | -83 | 1 | 0 | 2.52 | 39 | 0.34 | 1 | 1 | 1 | US | 1 | 0.14 | 4.0 | 40 | 60 | 62 | 46 | 0 | 0 | 0 | 0 | 1 | 2023 | C | 170 | 0.389 |
| (Karikari 2023) | E2 – S1B | 1 | 0 | -67 | 1 | 0 | 3.49 | 39 | 0.56 | 1 | 1 | 1 | US | 1 | 0.14 | 4.0 | 40 | 60 | 62 | 46 | 0 | 0 | 0 | 0 | 1 | 2023 | C | 203 | -0.322 |
| (Karikari 2023) | E2 – S1B | 1 | 0 | -67 | 1 | 0 | 3.49 | 39 | 0.56 | 1 | 1 | 1 | US | 1 | 0.14 | 4.0 | 40 | 60 | 62 | 46 | 0 | 0 | 0 | 0 | 1 | 2023 | C | 203 | -0.172 |
| (Karikari 2023) | E2 – S2 | 0 | 0 | -74 | 1 | 0 | 3.9 | 41 | 0.55 | 1 | 1 | 1 | US | 1 | 0.14 | 4.0 | 40 | 60 | 62 | 46 | 0 | 0 | 0 | 0 | 1 | 2023 | C | 174 | 0.802 |
| (Karikari 2023) | E2 – S2 | 0 | 0 | -74 | 1 | 0 | 3.9 | 41 | 0.55 | 1 | 1 | 1 | US | 1 | 0.14 | 4.0 | 40 | 60 | 62 | 46 | 0 | 0 | 0 | 0 | 1 | 2023 | C | 174 | -0.220 |
| (Karikari 2023) | E2 – S3 | 1 | 0 | -68 | 1 | 0 | 2.99 | 40 | 0.39 | 1 | 1 | 1 | US | 1 | 0.14 | 4.0 | 40 | 60 | 62 | 46 | 0 | 0 | 0 | 0 | 1 | 2023 | C | 496 | 0.146 |
| (Karikari 2023) | E2 – S3 | 1 | 0 | -68 | 1 | 0 | 2.99 | 40 | 0.39 | 1 | 1 | 1 | US | 1 | 0.14 | 4.0 | 40 | 60 | 62 | 46 | 0 | 0 | 0 | 0 | 1 | 2023 | C | 496 | 0.360 |
| (Karikari 2023) | E3 – S1 | 0 | 0 | -68 | 1 | 0 | 3.13 | 35 | 0.34 | 1 | 1 | 1 | US | 1 | 0.14 | 4.0 | 40 | 60 | 62 | 46 | 0 | 0 | 0 | 0 | 1 | 2023 | P | 164 | -0.461 |
| (Karikari 2023) | E3 – S2 | 0 | 0 | -67 | 1 | 0 | 3.39 | 41 | 0.57 | 1 | 1 | 1 | US | 1 | 0.14 | 4.0 | 40 | 60 | 62 | 46 | 0 | 0 | 0 | 0 | 1 | 2023 | P | 345 | 0.128 |
| (Karikari 2023) | E3 – S2 | 0 | 0 | -67 | 1 | 0 | 3.39 | 41 | 0.57 | 1 | 1 | 1 | US | 1 | 0.14 | 4.0 | 40 | 60 | 62 | 46 | 0 | 0 | 0 | 0 | 1 | 2023 | P | 345 | 0.219 |
| (Karikari 2023) | E3 – S3 | 0 | 0 | -83 | 1 | 0 | 2.71 | 40 | 0.33 | 1 | 0 | 1 | US | 1 | 0.14 | 4.0 | 40 | 60 | 62 | 46 | 0 | 0 | 0 | 0 | 1 | 2023 | P | 221 | 0.131 |
| (Karikari 2023) | E3 – S3 | 0 | 0 | -83 | 1 | 0 | 2.71 | 40 | 0.33 | 1 | 0 | 1 | US | 1 | 0.14 | 4.0 | 40 | 60 | 62 | 46 | 0 | 0 | 0 | 0 | 1 | 2023 | P | 221 | 0.281 |
| (Lee 2023) | S3 | 0 | 0 | -71 | 0 | 0 | 3.72 | 41 | 0.50 | 0 | 0 | 1 | US | 1 | 0.14 | 4.0 | 40 | 60 | 62 | 46 | 0 | 0 | 0 | 0 | 0 | 2023 | C | 488 | 0.165 |
| (Lee 2023) | S3 | 0 | 0 | -71 | 0 | 0 | 3.72 | 41 | 0.50 | 0 | 0 | 1 | US | 1 | 0.14 | 4.0 | 40 | 60 | 62 | 46 | 0 | 0 | 0 | 0 | 0 | 2023 | C | 488 | 0.416 |
| (Lim et al. 2023) | - | 1 | 0 | -50 | 0 | 0 | 3.72 | 32 | 0.39 | 0 | 1 | 1 | US | 1 | 0.14 | 4.0 | 40 | 60 | 62 | 46 | 1 | 0 | 0 | 0 | 0 | 2023 | A | 300 | 0.419 |
| (Lim et al. 2023) | - | 1 | 0 | -50 | 0 | 0 | 3.72 | 32 | 0.39 | 0 | 1 | 1 | US | 1 | 0.14 | 4.0 | 40 | 60 | 62 | 46 | 1 | 0 | 0 | 0 | 0 | 2023 | C | 300 | 0.194 |
| (Lim et al. 2023) | - | 1 | 0 | -50 | 0 | 0 | 3.72 | 32 | 0.39 | 0 | 1 | 1 | US | 1 | 0.14 | 4.0 | 40 | 60 | 62 | 46 | 1 | 0 | 0 | 0 | 0 | 2023 | P | 300 | 0.143 |
| (Lim et al. 2023) | - | 1 | 0 | -50 | 0 | 0 | 3.72 | 32 | 0.39 | 0 | 1 | 1 | US | 1 | 0.14 | 4.0 | 40 | 60 | 62 | 46 | 1 | 0 | 0 | 0 | 0 | 2023 | P | 300 | 0.509 |
| (Mello 2022) | S1 | 1 | 0 | -63 | 1 | 0 | 2.04 | 25 | 0.50 | 0 | 0 | 0 | Brazil | 0 | -0.21 | 3.0 | 69 | 36 | 49 | 76 | 0 | 0 | 0 | 0 | 1 | 2022 | A | 194 | 0.014 |
| (Mello 2022) | S1 | 1 | 0 | -63 | 1 | 0 | 2.04 | 25 | 0.50 | 0 | 0 | 0 | Brazil | 0 | -0.21 | 3.0 | 69 | 36 | 49 | 76 | 0 | 0 | 0 | 0 | 1 | 2022 | C | 194 | 0.011 |
| (Mello 2022) | S1 | 1 | 0 | -63 | 1 | 0 | 2.04 | 25 | 0.50 | 0 | 0 | 0 | Brazil | 0 | -0.21 | 3.0 | 69 | 36 | 49 | 76 | 0 | 0 | 0 | 0 | 1 | 2022 | E | 194 | 0.000 |
| (Mello 2022) | S2 | 1 | 0 | -63 | 0 | 0 | 3.72 | 37 | 0.63 | 0 | 0 | 0 | US | 1 | 0.14 | 4.0 | 40 | 60 | 62 | 46 | 0 | 0 | 0 | 0 | 1 | 2022 | A | 479 | 0.003 |
| (Mello 2022) | S2 | 1 | 0 | -63 | 0 | 0 | 3.72 | 37 | 0.63 | 0 | 0 | 0 | US | 1 | 0.14 | 4.0 | 40 | 60 | 62 | 46 | 0 | 0 | 0 | 0 | 1 | 2022 | A | 479 | 0.044 |
| (Mello 2022) | S2 | 1 | 0 | -63 | 0 | 0 | 3.72 | 37 | 0.63 | 0 | 0 | 0 | US | 1 | 0.14 | 4.0 | 40 | 60 | 62 | 46 | 0 | 0 | 0 | 0 | 1 | 2022 | C | 479 | 0.017 |
| (Mello 2022) | S2 | 1 | 0 | -63 | 0 | 0 | 3.72 | 37 | 0.63 | 0 | 0 | 0 | US | 1 | 0.14 | 4.0 | 40 | 60 | 62 | 46 | 0 | 0 | 0 | 0 | 1 | 2022 | E | 479 | 0.004 |
| (Mesquita et al. 2022) | - | 0 | 0 | -80 | 0 | 1 | 4.67 | 26 | 0.40 | 0 | 0 | 0 | Brazil | 0 | -0.21 | 3.0 | 69 | 36 | 49 | 76 | 1 | 0 | 0 | 0 | 0 | 2022 | P | 206 | 0.326 |
| (Mesquita et al. 2022) | - | 0 | 0 | -80 | 0 | 1 | 4.67 | 26 | 0.40 | 0 | 0 | 0 | Brazil | 0 | -0.21 | 3.0 | 69 | 36 | 49 | 76 | 1 | 0 | 0 | 0 | 0 | 2022 | E | 206 | -0.242 |
| (Meyer 2022) | - | 1 | 0 | -82 | 0 | 0 | 3.72 | 23 | 0.49 | 0 | 0 | 0 | The Netherlands | 0 | 0.02 | 3.0 | 38 | 100 | 14 | 53 | 0 | 0 | 0 | 0 | 1 | 2022 | P | 217 | 0.171 |
| (Meyer 2022) | - | 1 | 0 | -81 | 0 | 0 | 3.72 | 23 | 0.49 | 0 | 0 | 0 | The Netherlands | 0 | 0.02 | 3.0 | 38 | 100 | 14 | 53 | 0 | 0 | 0 | 0 | 1 | 2022 | C | 217 | 0.192 |
| (Meyer 2022) | - | 1 | 0 | -80 | 0 | 0 | 3.72 | 23 | 0.49 | 0 | 0 | 0 | The Netherlands | 0 | 0.02 | 3.0 | 38 | 100 | 14 | 53 | 0 | 0 | 0 | 0 | 1 | 2022 | C | 217 | 0.351 |
| (Naouri 2022) | S2 | 1 | 0 | -71 | 0 | 0 | 3.72 | 23 | 0.73 | 0 | 0 | 0 | Morocco | 0 | 0.12 | 1.5 | 54.4 | 58.06 | 49.33 | 61.26 | 0 | 0 | 0 | 0 | 1 | 2022 | A | 200 | 0.067 |
| (Naouri 2022) | S2 | 1 | 0 | -71 | 0 | 0 | 3.72 | 23 | 0.73 | 0 | 0 | 0 | Morocco | 0 | 0.12 | 1.5 | 54.4 | 58.06 | 49.33 | 61.26 | 0 | 0 | 0 | 0 | 1 | 2022 | A | 200 | 0.050 |
| (Naouri 2022) | S2 | 1 | 0 | -71 | 0 | 0 | 3.72 | 23 | 0.73 | 0 | 0 | 0 | Morocco | 0 | 0.12 | 1.5 | 54.4 | 58.06 | 49.33 | 61.26 | 0 | 0 | 0 | 0 | 1 | 2022 | A | 200 | 0.235 |
| (Naouri 2022) | S2 | 1 | 0 | -71 | 0 | 0 | 3.72 | 23 | 0.73 | 0 | 0 | 0 | Morocco | 0 | 0.12 | 1.5 | 54.4 | 58.06 | 49.33 | 61.26 | 0 | 0 | 0 | 0 | 1 | 2022 | A | 200 | 0.065 |
| (Panizzo 2021) | - | 1 | 0 | -63 | 0 | 0 | 3.72 | 35 | 0.43 | 0 | 0 | 0 | Italy | 0 | -0.11 | 3.0 | 50 | 53 | 70 | 75 | 0 | 0 | 0 | 0 | 1 | 2021 | C | 211 | 0.177 |
| (Panizzo 2021) | - | 1 | 0 | -63 | 0 | 0 | 3.72 | 35 | 0.50 | 0 | 0 | 0 | Italy | 0 | -0.11 | 3.0 | 50 | 53 | 70 | 75 | 0 | 0 | 0 | 0 | 1 | 2021 | C | 211 | 0.180 |
| (Panizzo 2021) | - | 1 | 0 | -63 | 0 | 0 | 3.72 | 35 | 0.50 | 0 | 0 | 0 | Italy | 0 | -0.11 | 3.0 | 50 | 53 | 70 | 75 | 0 | 0 | 0 | 0 | 1 | 2021 | C | 211 | 0.179 |
| (Reichert et al. 2022) | - | 1 | 0 | -67 | 0 | 0 | 3.72 | 36 | 0.50 | 0 | 0 | 0 | Germany | 0 | 0.32 | 3.0 | 35 | 79 | 66 | 65 | 0 | 0 | 0 | 0 | 1 | 2022 | W | 183 | -0.432 |
| (Reichert et al. 2022) | - | 1 | 0 | -67 | 0 | 0 | 3.72 | 36 | 0.50 | 0 | 0 | 0 | Germany | 0 | 0.32 | 3.0 | 35 | 79 | 66 | 65 | 0 | 0 | 0 | 0 | 1 | 2022 | W | 183 | -0.196 |
| (Schleier 2021) | - | 1 | 0 | -87 | 0 | 0 | 3.72 | 26 | 0.25 | 0 | 1 | 0 | Various | 0 | 0.00 | 2.6 | 70 | 30 | 40 | 30 | 0 | 0 | 0 | 0 | 1 | 2021 | C | 328 | 0.580 |
| (Song and Lan 2022) | - | 1 | 0 | -63 | 0 | 0 | 3.72 | 32 | 0.58 | 0 | 0 | 0 | China | 0 | 0.12 | 1.0 | 80 | 43 | 66 | 30 | 1 | 0 | 0 | 0 | 0 | 2022 | C | 418 | 0.730 |
| (Valerie and Hariandja 2022) | - | 0 | 0 | -71 | 0 | 0 | 3.72 | 28 | 0.50 | 0 | 0 | 0 | Indonesia | 0 | -0.01 | 1.0 | 78 | 5 | 46 | 48 | 0 | 0 | 0 | 0 | 0 | 2022 | C | 460 | 0.233 |
| (Valerie and Hariandja 2022) | - | 0 | 0 | -71 | 0 | 0 | 3.72 | 28 | 0.50 | 0 | 0 | 0 | Indonesia | 0 | -0.01 | 1.0 | 78 | 5 | 46 | 48 | 0 | 0 | 0 | 0 | 0 | 2022 | P | 460 | 0.113 |
| (Vasquez 2022) | - | 1 | 0 | -87 | 1 | 0 | 3.93 | 36 | 0.52 | 0 | 1 | 1 | US | 1 | 0.14 | 4.0 | 40 | 60 | 62 | 46 | 1 | 0 | 0 | 0 | 1 | 2022 | C | 1048 | 0.116 |
| (Vasquez 2022) | - | 1 | 0 | -87 | 1 | 0 | 3.93 | 36 | 0.52 | 0 | 1 | 1 | US | 1 | 0.14 | 4.0 | 40 | 60 | 62 | 46 | 1 | 0 | 0 | 0 | 1 | 2022 | C | 1048 | 0.188 |
| (Vasquez 2022) | - | 1 | 0 | -87 | 1 | 0 | 3.93 | 36 | 0.52 | 0 | 1 | 1 | US | 1 | 0.14 | 4.0 | 40 | 60 | 62 | 46 | 1 | 0 | 0 | 0 | 1 | 2022 | P | 1048 | 0.131 |
| (Villagra et al. 2021) | - | 0 | 0 | -71 | 0 | 0 | 3.72 | 44 | 0.50 | 0 | 0 | 0 | Spain | 0 | -0.19 | 4.0 | 57 | 67 | 42 | 86 | 1 | 0 | 0 | 0 | 0 | 2021 | C | 1521 | 0.042 |
| (Villagra et al. 2021) | - | 0 | 0 | -71 | 0 | 0 | 3.72 | 44 | 0.50 | 0 | 0 | 0 | Spain | 0 | -0.19 | 4.0 | 57 | 67 | 42 | 86 | 1 | 0 | 0 | 0 | 0 | 2021 | C | 1521 | 0.042 |
| (Villagra et al. 2021) | - | 0 | 0 | -71 | 0 | 0 | 3.72 | 44 | 0.50 | 0 | 0 | 0 | Spain | 0 | -0.19 | 4.0 | 57 | 67 | 42 | 86 | 1 | 0 | 0 | 0 | 0 | 2021 | C | 1521 | 0.152 |
| (Villagra et al. 2021) | - | 0 | 0 | -71 | 0 | 0 | 3.72 | 44 | 0.50 | 0 | 0 | 0 | Spain | 0 | -0.19 | 4.0 | 57 | 67 | 42 | 86 | 1 | 0 | 0 | 0 | 0 | 2021 | C | 1521 | 0.155 |
| (Wang et al. 2021) | S1 | 0 | 0 | -63 | 0 | 0 | 3.72 | 29 | 0.45 | 0 | 1 | 0 | Taiwan | 0 | -0.16 | 1.0 | 58 | 40 | 45 | 69 | 1 | 0 | 0 | 0 | 1 | 2021 | P | 177 | -0.517 |
| (Wang et al. 2021) | S2 | 1 | 0 | -63 | 0 | 0 | 3.72 | 36 | 0.44 | 0 | 1 | 0 | Taiwan | 0 | -0.16 | 1.0 | 58 | 40 | 45 | 69 | 1 | 0 | 0 | 0 | 1 | 2021 | P | 247 | -0.497 |
| (Xie 2022) | S2 | 1 | 0 | -83 | 1 | 0 | 3.77 | 39 | 0.48 | 1 | 0 | 1 | US | 1 | 0.14 | 4.0 | 40 | 60 | 62 | 46 | 0 | 0 | 0 | 0 | 0 | 2022 | P | 239 | 0.172 |
| (Xie 2022) | S2 | 1 | 0 | -83 | 1 | 0 | 3.77 | 39 | 0.48 | 1 | 0 | 1 | US | 1 | 0.14 | 4.0 | 40 | 60 | 62 | 46 | 0 | 0 | 0 | 0 | 0 | 2022 | P | 239 | 0.171 |
| (Xie 2022) | S2 | 1 | 0 | -83 | 1 | 0 | 3.77 | 39 | 0.48 | 1 | 0 | 1 | US | 1 | 0.14 | 4.0 | 40 | 60 | 62 | 46 | 0 | 0 | 0 | 0 | 0 | 2022 | P | 220 | 0.172 |
| (Xie 2022) | S2 | 1 | 0 | -83 | 1 | 0 | 3.77 | 39 | 0.48 | 1 | 0 | 1 | US | 1 | 0.14 | 4.0 | 40 | 60 | 62 | 46 | 0 | 0 | 0 | 0 | 0 | 2022 | P | 220 | 0.175 |
| (Young 2017) | - | 1 | 0 | -63 | 0 | 0 | 3.72 | 36 | 0.54 | 1 | 0 | 1 | US | 1 | 0.14 | 4.0 | 40 | 60 | 62 | 46 | 0 | 0 | 0 | 0 | 0 | 2017 | C | 550 | -0.072 |
| (Young 2017) | - | 1 | 0 | -63 | 0 | 0 | 3.72 | 36 | 0.54 | 1 | 0 | 1 | US | 1 | 0.14 | 4.0 | 40 | 60 | 62 | 46 | 0 | 0 | 0 | 0 | 0 | 2017 | C | 550 | -0.156 |
| (Young 2017) | - | 1 | 0 | -63 | 0 | 0 | 3.72 | 36 | 0.54 | 1 | 0 | 1 | US | 1 | 0.14 | 4.0 | 40 | 60 | 62 | 46 | 0 | 0 | 0 | 0 | 0 | 2017 | C | 550 | -0.045 |
| (Young 2017) | - | 1 | 0 | -63 | 0 | 0 | 3.72 | 36 | 0.54 | 1 | 0 | 1 | US | 1 | 0.14 | 4.0 | 40 | 60 | 62 | 46 | 0 | 0 | 0 | 0 | 0 | 2017 | C | 550 | 0.494 |
| (Young 2017) | - | 1 | 0 | -63 | 0 | 0 | 3.72 | 36 | 0.54 | 1 | 0 | 1 | US | 1 | 0.14 | 4.0 | 40 | 60 | 62 | 46 | 0 | 0 | 0 | 0 | 0 | 2017 | C | 550 | 0.086 |
| (Young 2017) | - | 1 | 0 | -63 | 0 | 0 | 3.72 | 36 | 0.54 | 1 | 0 | 1 | US | 1 | 0.14 | 4.0 | 40 | 60 | 62 | 46 | 0 | 0 | 0 | 0 | 0 | 2017 | C | 550 | 0.416 |
| (Zhou and Dong 2022) | - | 1 | 0 | -87 | 0 | 0 | 3.72 | 37 | 0.54 | 0 | 0 | 1 | US | 1 | 0.14 | 4.0 | 40 | 60 | 62 | 46 | 1 | 0 | 0 | 0 | 1 | 2022 | P | 244 | -0.232 |
| (Zhou and Dong 2022) | - | 1 | 0 | -87 | 0 | 0 | 3.72 | 37 | 0.54 | 0 | 0 | 1 | US | 1 | 0.14 | 4.0 | 40 | 60 | 62 | 46 | 1 | 0 | 0 | 0 | 1 | 2022 | P | 244 | -0.156 |
| (Zhou and Dong 2022) | - | 1 | 0 | -87 | 0 | 0 | 3.72 | 37 | 0.54 | 0 | 0 | 1 | US | 1 | 0.14 | 4.0 | 40 | 60 | 62 | 46 | 1 | 0 | 0 | 0 | 1 | 2022 | P | 244 | -0.303 |
| (Zhou and Dong 2022) | - | 1 | 0 | -87 | 0 | 0 | 3.72 | 37 | 0.54 | 0 | 0 | 1 | US | 1 | 0.14 | 4.0 | 40 | 60 | 62 | 46 | 1 | 0 | 0 | 0 | 1 | 2022 | P | 244 | -0.167 |

*Note:* 1 = ID; 2 = Study; 3 = CA liberal political leaning; 4 = CA conservative political leaning; 5 = Sociopolitical issue controversy level, reversed coded; 6 = Liberals ; 7 = Conservatives; 8 = Audience political orientation; 9 = Age; 10 = Male gender (%); 11 = Income; 12 = Education; 13 = Racial diversity; 14 = Country; 15 = Country of residence (US); 16 = World Values Survey index to capture political involvement in a country; 17 = Edelman polarization level; 18 = Power distance; 19 = Individualism; 20 = Masculinity; 21 = Uncertainty avoidance; 22 = Publication status; 23 = ABDC ranking; 24 = ABDC ranking – continuous; 25 = Top journal; 26 = Study method; 27 = Year; 28 = Public reaction categories, in which A: Ads and social media engagement, C: Cognitive and attitudinal reactions, P: Public's intentions and actions, E: Emotional reactions, S: Social and ethical engagement, W: Workplace and employee perceptions; 29 = Sample size; 30 = Adjusted correlation.

# Appendix B: Univariate analysis

When examining the different impact of CA on various outcomes, our findings reveal notable variance in ES. Social and ethical engagement exhibits the strongest positive effect (r = 0.1895, 95% CI [0.0330, 0.3370]), followed by cognitive and attitudinal reactions (r = 0.1258, 95% CI [0.0721, 0.1786]). Emotional reactions and the public’s intentions and actions also show positive effects (r = 0.0919, 95% CI [0.0050, 0.1774], and r = 0.0512, 95% CI [0.0026, 0.0996], respectively), albeit to a lesser extent. In contrast, ads and social media engagement, as well as workplace and employee perceptions display non-significant effects (r = -0.0033, 95% CI [-0.1324, 0.1258], and r = -0.0081, 95% CI [-0.0858, 0.0697], respectively).

Per the moderating role of CA characteristics, a moderator analysis first reveals that liberal-leaning CA has a positive ES (r = 0.115 (95% CI [0.076, 0.154]). Conversely, conservative-leaning CA elicits a negative ES (r = -0.088 (95% CI [-0.148, -0.027]). Second, results show that less controversial sociopolitical issues generate a slightly positive ES (r = 0.055, 95% CI [0.011, 0.099]) compared to more controversial issues, which show an effect not different than zero (r = 0.024, 95% CI [-0.020, 0.067]).

Per target audience characteristics, considering the impact of the target audience’s political orientation, results show a small positive effect among liberals (r = 0.070, 95% CI [0.011, 0.128]) and a non-significant effect among conservatives (r = 0.091, 95% CI [-0.018, 0.198]). Per demographics, gender differences were pronounced, with female respondents demonstrating a positive response (r = 0.182, 95% CI [0.125, 0.239]), compared to the non-significant effect observed among male respondents (r = 0.017, 95% CI [-0.022, 0.057]). Income levels further influenced response, with the higher-income group showing a strong positive effect (r = 0.401, 95% CI [0.254, 0.530]), while the lower-income group exhibited a moderate positive effect (r = 0.127, 95% CI [0.009, 0.241]). Education played a role as well, where individuals with higher education levels respond more favorably (r = 0.205, 95% CI [0.125, 0.281]) compared to those with lower education (r = 0.127, 95% CI [0.040, 0.211]). Racial diversity indicated positive effects when either more (r = 0.201, 95% CI [0.097, 0.300]) or less (r = 0.166, 95% CI [0.092, 0.239]) racially diverse groups were studied. Per age, younger audiences showed a more favorable response (r = 0.166, 95% CI [0.109, 0.221]) compared to older audiences (r = 0.073, 95% CI [0.024, 0.121]). Finally, a minor discrepancy between US residents (r= 0.077, 95% CI [0.042, 0.112]) versus non-US ones (r= 0.113, 95% CI [0.050, 0.175]) was found, with both positively affecting responses to CA. The impact of other country and national culture characteristics are discussed below.

Per the methodological moderators, when considering the difference between published (r = 0.078, 95% CI [0.041, 0.115]) and unpublished work (r = 0.104, 95% CI [0.047, 0.160]), as well as across journal rankings from 'A*' with the ABDC ranking (r = 0.141, 95% CI [0.073, 0.208]) to 'C' ranked (r = -0.096, 95% CI [-0.250, 0.063]), small or no differences were identified. Inclusion in the Financial Times' (FT) top journals list also did not alter the observed effect (r = 0.027, 95% CI [-0.076, 0.130] for FT-listed journals). Although minor, these differences do attest to the methodological rigor or thematic focus of the higher quality publishing outlets. Study method revealed no significance for experiments (r = 0.0051, 95% CI [-0.0250, 0.0352]), and a positive effect of study method other than experimental (r = 0.2965, 95% CI [0.2336, 0.3568]) – mostly based on survey work and some big data studies. Table SB1 summarizes the results.

## Table S5. Univariate analysis

| **Variables** | **k** | **r** | **95%-CI-L** | **95%-CI-U** | **tau^2** | **tau** | **Q** | **I^2** |
| --- | --- | --- | --- | --- | --- | --- | --- | --- |
| Direct effect | 448 | 0.085 | 0.054 | 0.116 | 0.109 | 0.329 | 36276 | 98.80% |
| **Reaction to CA** |  |  |  |  |  |  |  |  |
| Ads and social media engagement | 13 | -0.003 | -0.132 | 0.126 | 0.054 | 0.232 | 209 | 94.30% |
| Cognitive and attitudinal reactions | 179 | 0.126 | 0.072 | 0.179 | 0.132 | 0.364 | 11678 | 98.50% |
| Public's intentions and actions | 146 | 0.051 | 0.003 | 0.100 | 0.087 | 0.295 | 17710 | 99.20% |
| Emotional reactions | 35 | 0.092 | 0.005 | 0.177 | 0.062 | 0.248 | 416 | 91.80% |
| Social and ethical engagement | 28 | 0.190 | 0.033 | 0.337 | 0.181 | 0.426 | 2302 | 98.80% |
| Workplace and employee perceptions | 47 | -0.008 | -0.086 | 0.070 | 0.072 | 0.268 | 2674 | 98.30% |
| **Main moderators** |  |  |  |  |  |  |  |  |
| *CA political leaning* |  |  |  |  |  |  |  |  |
| Liberal | 248 | 0.115 | 0.076 | 0.154 | 0.097 | 0.312 | 21801 | 98.90% |
| Conservative | 50 | -0.088 | -0.148 | -0.027 | 0.046 | 0.215 | 2945 | 98.30% |
| *Sociopolitical Issue controversy level* |  |  |  |  |  |  |  |  |
| Higher | 195 | 0.024 | -0.020 | 0.067 | 0.093 | 0.304 | 4953 | 96.10% |
| Lower | 163 | 0.055 | 0.011 | 0.099 | 0.080 | 0.283 | 22009 | 99.30% |
| *Target audience political orientation* |  |  |  |  |  |  |  |  |
| Liberal | 127 | 0.070 | 0.011 | 0.128 | 0.111 | 0.334 | 13386 | 99.10% |
| Conservative | 38 | 0.091 | -0.018 | 0.198 | 0.113 | 0.336 | 1156 | 96.80% |
| *Age* |  |  |  |  |  |  |  |  |
| Older | 214 | 0.073 | 0.024 | 0.121 | 0.129 | 0.360 | 11369 | 98.10% |
| Younger | 138 | 0.166 | 0.109 | 0.221 | 0.113 | 0.337 | 5739 | 97.60% |
| *Gender* |  |  |  |  |  |  |  |  |
| Female | 157 | 0.182 | 0.125 | 0.239 | 0.136 | 0.369 | 8923 | 98.30% |
| Male | 209 | 0.017 | -0.022 | 0.057 | 0.081 | 0.285 | 5599 | 96.30% |
| *Income* |  |  |  |  |  |  |  |  |
| Higher | 30 | 0.401 | 0.254 | 0.530 | 0.211 | 0.459 | 2979 | 99.00% |
| Lower | 32 | 0.127 | 0.009 | 0.241 | 0.114 | 0.337 | 1354 | 97.70% |
| *Education* |  |  |  |  |  |  |  |  |
| Higher | 99 | 0.205 | 0.125 | 0.281 | 0.168 | 0.410 | 6480 | 98.50% |
| Lower | 40 | 0.127 | 0.040 | 0.211 | 0.074 | 0.272 | 640 | 93.90% |
| *Racial diversity* |  |  |  |  |  |  |  |  |
| More diverse | 61 | 0.201 | 0.097 | 0.300 | 0.175 | 0.419 | 4580 | 98.70% |
| Less diverse | 88 | 0.166 | 0.092 | 0.239 | 0.129 | 0.359 | 5084 | 98.30% |
| *Country of residence* |  |  |  |  |  |  |  |  |
| United States | 348 | 0.077 | 0.042 | 0.112 | 0.111 | 0.333 | 32010 | 98.90% |
| Not United States | 100 | 0.113 | 0.050 | 0.175 | 0.100 | 0.317 | 3596 | 97.20% |
| **Methodological characteristics** |  |  |  |  |  |  |  |  |
| *Publication status* |  |  |  |  |  |  |  |  |
| Published | 322 | 0.078 | 0.041 | 0.115 | 0.112 | 0.334 | 31182 | 99.00% |
| Unpublished | 126 | 0.104 | 0.047 | 0.160 | 0.101 | 0.318 | 4718 | 97.40% |
| *ABDC ranking* |  |  |  |  |  |  |  |  |
| A* | 75 | 0.141 | 0.073 | 0.208 | 0.088 | 0.296 | 2682 | 97.20% |
| A | 135 | 0.044 | -0.011 | 0.098 | 0.102 | 0.319 | 19864 | 99.30% |
| B | 84 | 0.071 | -0.001 | 0.143 | 0.112 | 0.334 | 5735 | 98.60% |
| C | 6 | -0.096 | -0.250 | 0.063 | 0.035 | 0.187 | 38 | 87.00% |
| Not ranked | 148 | 0.111 | 0.053 | 0.167 | 0.123 | 0.351 | 6670 | 97.80% |
| *Top journal* |  |  |  |  |  |  |  |  |
| Yes | 54 | 0.027 | -0.076 | 0.130 | 0.146 | 0.382 | 2875 | 98.20% |
| No | 394 | 0.093 | 0.061 | 0.125 | 0.103 | 0.321 | 33369 | 98.80% |
| *Study method* |  |  |  |  |  |  |  |  |
| Experiment: Yes | 330 | 0.0051 | -0.025 | 0.0352 | 0.0735 | 0.2711 | 10417.59 | 96.80% |
| Experiment: No | 118 | 0.2965 | 0.2336 | 0.3568 | 0.1377 | 0.371 | 25785.18 | 99.50% |
| *Year* |  |  |  |  |  |  |  |  |
| 2024 | 1 | 0.680 | 0.631 | 0.723 | -- | -- | 0 | -- |
| 2023 | 155 | 0.077 | 0.032 | 0.121 | 0.076 | 0.275 | 9088 | 98.30% |
| 2022 | 171 | 0.093 | 0.038 | 0.148 | 0.136 | 0.369 | 18090 | 99.10% |
| 2021 | 46 | 0.083 | -0.029 | 0.193 | 0.147 | 0.383 | 3604 | 98.80% |
| 2020 | 55 | 0.043 | -0.038 | 0.123 | 0.090 | 0.300 | 2813 | 98.10% |
| 2019 | 13 | 0.176 | 0.032 | 0.312 | 0.066 | 0.257 | 168 | 92.90% |
| 2017 | 6 | 0.099 | -0.099 | 0.289 | 0.058 | 0.240 | 93 | 94.60% |
| 2013 | 1 | 0.402 | 0.279 | 0.512 | -- | -- | 0 | -- |

*Note*: Variables definitions are available in SI Appendix A, Table SA2.

# Appendix C: Additional analyses

## Table S6. Country and national culture moderation analysis on response to CA

| **Variables/Model** | **Model 1** | **Model 2** | **Model 3** | **Model 4** | **Model 5** | **Model 6** |
| --- | --- | --- | --- | --- | --- | --- |
| Country political involvement | 0.263* | 0.112 |  |  |  |  |
|  | (0.117) | (0.141) |  |  |  |  |
| Country polarization level |  |  | 0.006 | -0.064 |  |  |
|  |  |  | (0.042) | (0.049) |  |  |
| Power Distance |  |  |  |  | 0.015* | 0.022** |
|  |  |  |  |  | (0.006) | (0.005) |
| Individualism |  |  |  |  | 0.008* | 0.011* |
|  |  |  |  |  | (0.004) | (0.005) |
| Masculinity |  |  |  |  | 0.001 | 0.001 |
|  |  |  |  |  | (0.003) | (0.003) |
| Uncertainty Avoidance |  |  |  |  | -0.008** | -0.007* |
|  |  |  |  |  | (0.003) | (0.003) |
| *CA political leaning* |  |  |  |  |  |  |
| Liberal |  | 0.0903*** |  | 0.0904*** |  | 0.0889*** |
|  |  | (0.015) |  | (0.015) |  | (0.015) |
| Conservative |  | -0.088*** |  | -0.088*** |  | -0.089*** |
|  |  | (0.015) |  | (0.015) |  | (0.015) |
| Sociopolitical issue controversy level |  | -0.0086*** |  | -0.009*** |  | -0.0089*** |
|  |  | (0.002) |  | (0.002) |  | (0.002) |
| *Target audience political orientation* | |  |  |  |  |  |
| Liberal |  | 0.4205*** |  | 0.4207*** |  | 0.4206*** |
|  |  | (0.005) |  | (0.005) |  | (0.005) |
| Conservative |  | 0.0725 |  | 0.0892 |  | 0.0824 |
|  |  | (0.109) |  | (0.110) |  | (0.107) |
| Intercept | 0.0585 | -0.722*** | 0.0701 | -0.4941* | -0.754 | -2.0559** |
|  | (0.032) | (0.158) | (0.158) | (0.235) | (0.519) | (0.640) |
| k | 448 | 448 | 448 | 448 | 448 | 448 |

*Note*: k is the number of effect sizes. The values in parentheses represent the standard errors. ∗p < .05; ∗∗p < .01; ∗∗∗p < .001.

Given that our sample is heavily skewed toward the United States (348 ES, 77%), we ran a robustness check for the country-level variables. Specifically, we re-ran the analysis without considering the US to test whether the significant relationships observed initially persisted across other geographical contexts. Results remain consistent, suggesting that our results are robust. Table SC2 presents these results. Additionally, we conducted a power analysis using G*Power to evaluate the statistical power of the models. Results revealed that Model 7 has a power of 0.79, Model 8 has a power of 0.15, and Model 9 has a power of 0.51.

## Table S7. Country and national culture moderation without US

|  | **Model 7** | **Model 8** | **Model 9** |
| --- | --- | --- | --- |
| Country political involvement | 0.282** |  |  |
|  | (0.095) |  |  |
| Country polarization level |  | -0.008 |  |
|  |  | (0.088) |  |
| Power Distance |  |  | 0.022* |
|  |  |  | (0.009) |
| Individualism |  |  | 0.012* |
|  |  |  | (0.005) |
| Masculinity |  |  | 0.000 |
|  |  |  | (0.003) |
| Uncertainty Avoidance |  |  | -0.007* |
|  |  |  | (0.003) |
| Intercept | 0.050 | 0.097 | -1.472 |
|  | (0.073) | (0.246) | (0.830) |
| k | 100 | 100 | 100 |

*Note*: k is the number of effect sizes. The variables age and gender were mean-centered. The values in parentheses represent the standard errors. ∗p < .05; ∗∗p < .01; ∗∗∗p < .001.

## Table S8. Dependent variable – by category of outcomes

| **Moderators** | **Model 1 Ad attitudes and engagement** | **Model 2 Cognitive and attitudinal reactions** | **Model 3 Public's intentions and actions** | **Model 4 Emotional reactions** | **Model 5 Social and ethical engagement** | **Model 6 Workplace and employee perceptions** |
| --- | --- | --- | --- | --- | --- | --- |
| **Main effect** | -0.0419 | 0.1682 *** | 0.073 | 0.1337* | 0.1724 | 0.0262 |
|  | (0.1023) | (0.0412) | (0.0395) | (0.0614) | (0.0937) | (0.0892) |
| **Main variables** |  |  |  |  |  |  |
| *CA political leaning* |  |  |  |  |  |  |
| Liberal | 0.1183 | 0.0864 ** | -0.0293 | 0.3771 | -0.2007 | -0.4316 |
|  | (0.2343) | (0.0332) | (0.1018) | (0.1944) | (0.2036) | (0.2343) |
| Conservative |  | -0.1396 *** | 0.0234 | 0.2835 |  | -0.5818 * |
|  |  | 0.0333 | (0.1069) | (0.2094) |  | (0.2343) |
| *Sociopolitical issue controversy level* | 0.0142 | -0.0047 | -0.0056 * | -0.0033 | 0.0098 | 0.0047 |
|  | (0.0081) | (0.0050) | (0.0025) | (0.0085) | (0.0144) | (0.0091) |
| *Target audience political orientation* |  |  |  |  |  |  |
| Liberal | -0.2582 | -0.0108 | 0.1625 | -0.0017 | 0.2601 | 0.0910 |
|  | (0.1789) | (0.0983) | (0.1438) | (0.2156) | (0.1859) | (0.2598) |
| Conservative |  | -0.0714 | 0.4216 *** | 0.2317 | -0.4812 |  |
|  |  | (0.1474) | (0.0054) | (0.2055) | (0.4004) |  |
| Intercept | 0.9398 | -0.2028 | -0.4948 ** | -0.4292 | 0.9152 | 0.7432 |
|  | (0.5783) | (0.3610) | (0.1911) | (0.6931) | (1.0535) | (0.6764) |
| k | 13 | 179 | 146 | 35 | 28 | 47 |

*Note*: Each DV category has effects sizes (k) from at least five different scholarly works. The small number of effect sizes in models 1, 5, and 6, resulted in high collinearity between moderators, with some moderators been dropped from the model. The values in parentheses represent the standard errors. ∗p < .05; ∗∗p < .01; ∗∗∗p < .001.

# Appendix D: Sensitivity analysis

## Table S9. Models of response to CA with continuous target audience political orientation

| **Variables** | **Model 1** | **Model 2** | **Model 3** | **Model 4** | **Model 5** |
| --- | --- | --- | --- | --- | --- |
| *Target audience political orientation* | -2.9510 *** | -2.9677 *** | -3.1069 *** | -2.6960 *** | -2.9570 *** |
|  | (0.1848) | (0.1856) | (0.1901) | (0.1913) | (0.1849) |
| *CA political leaning* |  |  |  |  |  |
| Liberal |  | 0.1048 *** | 0.1044 *** | 4.5036 *** | 0.1336 *** |
|  |  | (0.0182) | (0.0182) | (0.3839) | (0.0183) |
| Conservative |  | -0.0812 *** | -0.0814 *** | -0.1038 *** | -5.6933 *** |
|  |  | (0.0182) | (0.0182) | (0.0183) | (0.3961) |
| *Interactions* |  |  |  |  |  |
| Liberal CA x Target audience political orientation |  |  |  | -1.1639 *** | |
|  |  |  |  | (0.1015) |  |
| Conservative CA x Target audience political orientation |  |  |  |  | 1.4849 *** |
|  |  |  |  |  | (0.1047) |
| *Sociopolitical issue controversy level* |  | -0.0143 *** | -0.0142 *** | -0.0120 *** | -0.0112 *** |
|  |  | (0.0026) | (0.0026) | (0.0026) | (0.0026) |
| *Target audience demographics* |  |  |  |  |  |
| Age |  |  | 0.0663 |  |  |
|  |  |  | (0.0585) |  |  |
| Gender |  |  | 5.5337. |  |  |
|  |  |  | (3.0544) |  |  |
| Country of residence |  |  | 0.1702 |  |  |
|  |  |  | (1.1754) |  |  |
| **Methodological characteristics** |  |  |  |  |  |
| Publication status |  |  | -2.6694 * |  |  |
|  |  |  | (1.1479) |  |  |
| ABDC ranking |  |  | -0.7108. |  |  |
|  |  |  | (0.4108) |  |  |
| Top journal |  |  | 0.5170 |  |  |
|  |  |  | (1.3224) |  |  |
| Study method |  |  | 0.0757 |  |  |
|  |  |  | (0.6717) |  |  |
| Year |  |  | -0.4450 ** |  |  |
|  |  |  | (0.1632) |  |  |
| Intercept | 10.9118 *** | 9.8724 *** | 911.9326 ** | 8.9385 *** | 10.0620 *** |
|  | (0.7228) | (0.7538) | (330.3070) | (0.7811) | (0.7498) |
| k | 165 | 165 | 165 | 165 | 165 |
| *Note*: k is the number of effect sizes. The variables age and gender were mean-centered. The values in parentheses represent the standard errors. ∗p < .05; ∗∗p < .01; ∗∗∗p < .001. | | | | | |

## Table S10. Models of response to CA with unknown categories for main variables

| **Moderators** | **Model 1 Main variables only** | **Model 2 Main and control variables** |
| --- | --- | --- |
| **Main variables** |  |  |
| *CA characteristics* |  |  |
| CA political leaning: Liberal (vs Conservative) | 0.1786 *** | 0.1787 *** |
|  | (0.0053) | (0.0053) |
| CA political leaning: Unknown (vs Conservative) | 0.088 *** | 0.0878 *** |
|  | (0.0150) | (0.0150) |
| Sociopolitical issue controversy level | -0.0089*** | -0.0092 *** |
|  | (0.0021) | (0.0022) |
| *Target audience political orientation* |  |  |
| Political orientation: Liberal (vs Conservative) | 0.3469 ** | 0.3100 ** |
|  | (0.1098) | (0.1140) |
| Political orientation: Unknown (vs Conservative) | -0.7346 *** | -0.1107 |
|  | (0.1828) | (0.1139) |
| *Target audience demographics* |  |  |
| Age: Younger (vs Older) |  | -0.0121* |
|  |  | (0.0058) |
| Gender: Male (vs Female) |  | 0.1630 |
|  |  | (0.3714) |
| Income: Lower (vs Higher) |  | -0.2119 |
|  |  | (0.1748) |
| Income: Unknown (vs Higher) |  | -0.1422 |
|  |  | (0.1373) |
| Education: Lower (vs Higher) |  | -0.1673 |
|  |  | (0.1466) |
| Education: Unknown (vs Higher) |  | -0.0789 |
|  |  | (0.1051) |
| Racial diversity: More (vs Less) |  | -0.0746 |
|  |  | (0.1164) |
| Racial diversity: Unknown (vs Less) |  | 0.0978 |
|  |  | (0.1395) |
| Country: US (vs Not-US) |  | 0.0622 |
|  |  | (0.1060) |
| **Methodological characteristics** |  |  |
| Publication status: Unpublished (vs Published) |  | -0.0426 |
|  |  | (0.1241) |
| ABDC journal rank |  | -0.0120 |
|  |  | (0.0392) |
| Top journal: Yes (vs No) |  | 0.0170 |
|  |  | (0.1357) |
| Experiment: Yes (vs. No) |  | -0.3361*** |
|  |  | (0.0863) |
| Year |  | -0.0049 |
|  |  | (0.0250) |
| Intercept | -0.0736 | 9.6116 *** |
|  | (0.1098) | (50.6182) |
| k | 448 | 448 |
| *Note:* k is the number of effect sizes. The values in parentheses represent the standard errors. ∗p < .05; ∗∗p < .01; ∗∗∗p < .001. | | |

## Table S11. Models of response to CA for an alternative measure of target audience alignment with CA

| **Variable Name** | **Model 1** | **Model 2** |
| --- | --- | --- |
| Aligned audience | 0.0141 *** | 0.0141 *** |
|  | (0.0006) | (0.0006) |
| Misaligned audience | -0.0049 *** | -0.0049 *** |
|  | (0.0006) | (0.0006) |
| Sociopolitical issue controversy level |  | -0.0058 ** |
|  |  | (0.0020) |
| Age (continuous) |  | -0.0078 |
|  |  | (0.0043) |
| Gender (Male) |  | -0.1900 |
|  |  | (0.2839) |
| Country |  | 0.1127 |
|  |  | (0.0697) |
| Publication status |  | -0.0459 |
|  |  | (0.0900) |
| ABDC ranking |  | -0.0047 |
|  |  | (0.0292) |
| Top journal |  | -0.0476 |
|  |  | (0.1027) |
| Study method |  | -0.3203 *** |
|  |  | (0.0628) |
| Year |  | 0.0264 |
|  |  | (0.0185) |
| Intercept | 0.0901 ** | -53.5776 |
|  | (0.0284) | (37.3577) |
| k | 448 | 448 |
| *Note:* k is the number of effect sizes. The values in parentheses represent the standard errors. ∗p < .05; ∗∗p < .01; ∗∗∗p < .001 | | |

# Appendix E: Funnel plots


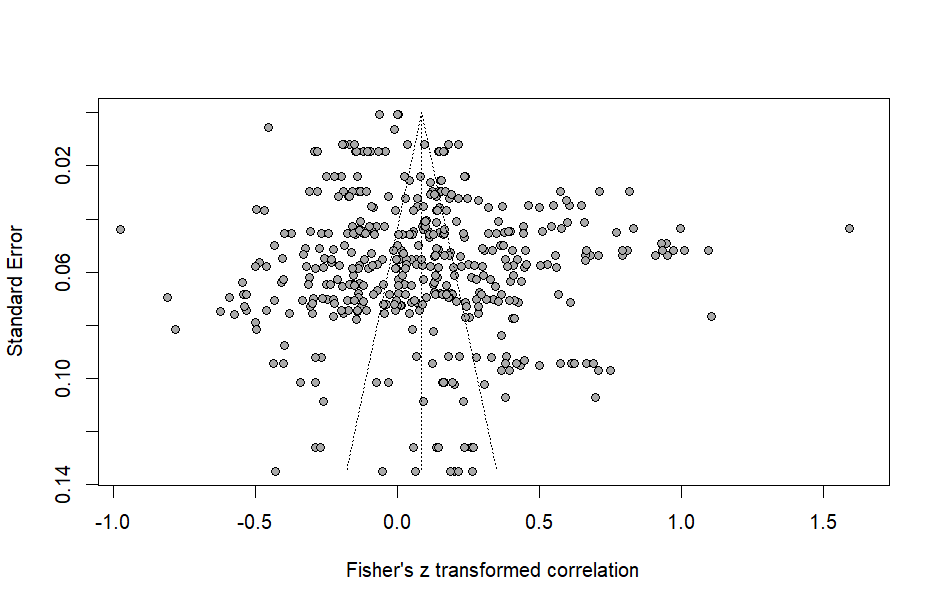


## Figure S1. Funnel plot of all effect sizes.


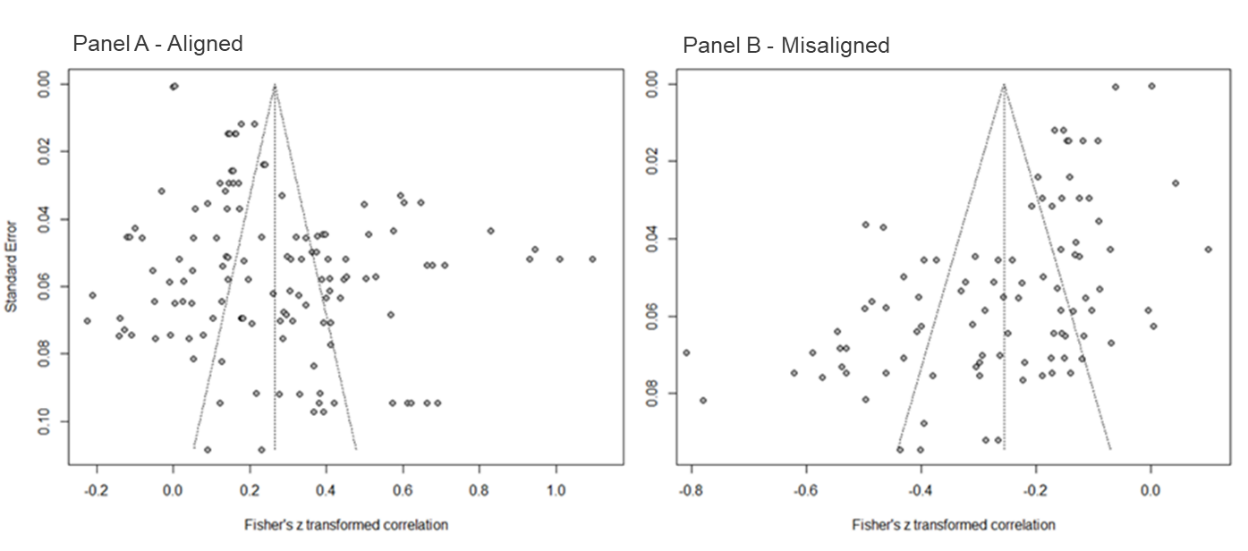


## Figure S2. Funnel plots of alignment between CA and audience political orientations (aligned vs. misaligned).


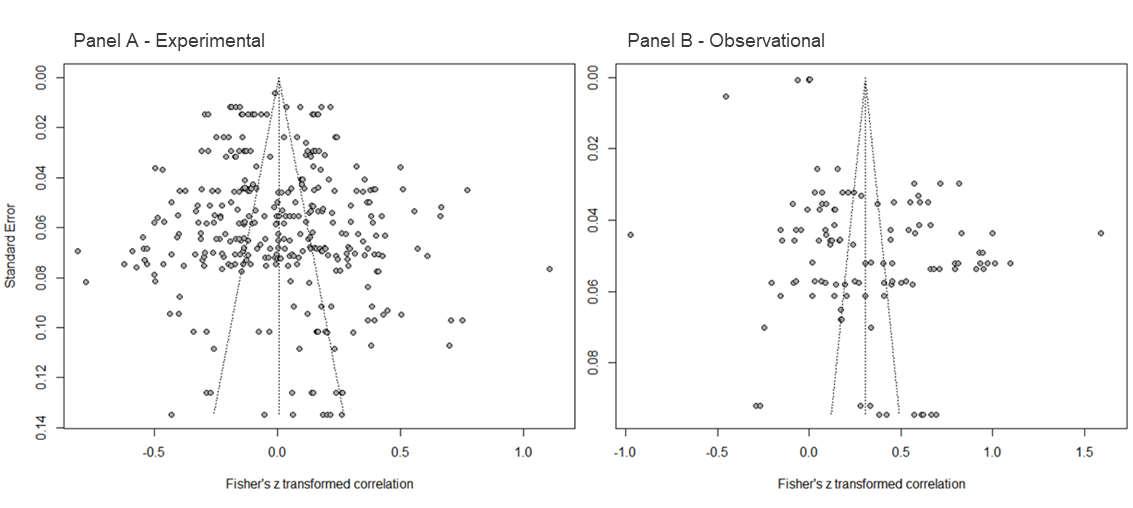


## Figure S3. Funnel plots of study methodology (experimental vs. observational).

# References

1. Global Strategy Group, “Business & Politics: Do They Mix? Third Annual Study” (2016).

2. Global Strategy Group, “Call Out Culture: Brands and Politics Collide in 2020. 7th Annual Business & Politics Study” (2020).

3. Global Strategy Group, “The Shifting Politics of Doing Good in America” (2023).

4. G. Hofstede, The Cultural Relativity of Organizational Practices and Theories. *J. Int. Bus. Stud.* 75–89 (1983).

5. C. Haerpfer, *et al.*, “World Values Survey: Round Seven” (2020).

6. Edelman, “2023 Edelman Trust Barometer: Global Report” (2023).

# References included in the meta-analysis (Table S4)

Ahmad F, Guzmán F, Kidwell B. 2022. Effective Messaging Strategies to Increase Brand Love for Sociopolitical Activist Brands. J Bus Res. 151(January):609–622. doi:10.1016/j.jbusres.2022.07.031.

Appels M. 2022. CEO Sociopolitical Activism as a Signal of Authentic Leadership to Prospective Employees. J Manage. 0(0):1–39. doi:10.1177/01492063221110207.

Arkema BF. 2022. Brand Activism and the Role of Authenticity and Identity Signalling. [Master dissertation, University of Twente]. http://essay.utwente.nl/93366/.

Atanga BA, Mattila AS. 2023. Corporate sociopolitical activism (CSA): The role of perceived impact on consumer response to contribution type. Int J Hosp Manag. 113(April):103506. doi:10.1016/j.ijhm.2023.103506.

Atanga BA, Xue X, Mattila AS. 2022. The Impact of Corporate Sociopolitical Activism (CSA) on Brand Attitude. Int J Hosp Manag. 107(August):103290. doi:10.1016/j.ijhm.2022.103290.

Bondi T, Burbano V, Dell’acqua F. 2023. When to Talk Politics in Business: Theory and Experimental Evidence of Stakeholder Responses to CEO Political Activism. http://www.vanessaburbano.com/uploads/2/5/0/4/25049117/bondi_burbano_dellacqua_whentotalkpolitics_20230213.pdf.

Burbano VC. 2021. The Demotivating Effects of Communicating a Social- Political Stance: Field Experimental Evidence from an Online Labor Market Platform. Manage Sci. 67(2):1004–1025. doi:10.1287/mnsc.2019.3562.

Burggraaf J, Larsson D. 2019. Speak Up or Stay Quiet: A Quantitative Study on the Influence of CEO/Brand Activism on Brand Equity and Purchase Intent in Sweden. [Master dissertation, Lund University]. https://lup.lub.lu.se/luur/download?func=downloadFile&recordOId=8990996&fileOId=8990997.

Chatterji AK, Toffel MW. 2019. Assessing the Impact of CEO Activism. Organ Environ. 32(2):159–185. doi:10.1177/1086026619848144.

Chu S-C, Kim H, Kim Y. 2022. When Brands Get Real: The Role of Authenticity and Electronic Word-of-Mouth in Shaping Consumer Response to Brands Taking a Stand. Int J Advert. 0(0):1–28. doi:10.1080/02650487.2022.2138057.

DiRusso C, Buckley C, Diddi P, Dardis FE, Vafeiadis M, Eng N. 2022. Designing Effective Corporate Social Advocacy Campaigns Using Valence, Arousal, and Issue Salience. Public Relat Rev. 48(3):102207. doi:10.1016/j.pubrev.2022.102207.

Dodd MD, Supa DW. 2013. Corporate Social Responsibility Re-Examined: Measuring the Influence of “Corporate Social Advocacy” Communication on Consumer Purchase Intention. In: 16th International Public Relations Research Conference. p. 232–245.

Edelblum AB. 2022. Stakeholder Implications of Corporate Sociopolitical Activism. [Doctoral dissertation, University of Oregon]. https://www.proquest.com/docview/2734685855?pq-origsite=gscholar&fromopenview=true.

Enete S, Sturr T. 2022. How Corporate Sociopolitical Activism (CSA) Impacts Portfolio Allocations: An Experiment. SSRN Electron J.:[availabe at: https://ssrn.com/abstract=4011877]. doi:10.2139/ssrn.4011877.

Fernandes D. 2020. Politics at the Mall: The Moral Foundations of Boycotts. J Public Policy Mark. 39(4):494–513. doi:10.1177/0743915620943178.

Garg N, Saluja G. 2022. A Tale of Two “Ideologies”: Differences in Consumer Response to Brand Activism. J Assoc Consum Res. 7(3):325–339. doi:10.1086/719584.

Goncalves RFA. 2021. The Power of Controversy, The Allure of Controversial Brands and People. [Master dissertation, Universidade Nova de Lisboa]. https://www.proquest.com/docview/2674875393?pq-origsite=gscholar&fromopenview=true.

Hamelberg K, de Ruyter K, van Dolen W, Konuş U. 2024. Finding the Right Voice: How CEO Communication on the Russia-Ukraine War Drives Public Engagement and Digital Activism. J Public Policy Mark. doi:10.1177/07439156241230910.

Haupt M, Wannow S, Marquardt L, Graubner JS, Haas A. 2023. Who is more responsive to brand activism? The role of consumer-brand identification and political ideology in consumer responses to activist brand messages. J Prod Brand Manag.(April). doi:10.1108/JPBM-10-2022-4193.

Heffron E. 2019. Nike’s Corporate Social Advocacy (CSA) Practices as Related to Strategic Issues Management (SIM) and Threats to Organizational Legitimacy. [Master dissertation, University of Central Florida]. https://stars.library.ucf.edu/etd/6502/.

Hong C, Li C. 2020. To Support or to Boycott : A Public Segmentation Model in Corporate Social Advocacy. J Public Relations Res. 32(5–6):160–167. doi:10.1080/1062726X.2020.1848841.

Hong C, Li C. 2021. Will Consumers Silence Themselves When Brands Speak up about Sociopolitical Issues? Applying the Spiral of Silence Theory to Consumer Boycott and Buycott Behaviors. J Nonprofit Public Sect Mark. 33(2):193–211. doi:10.1080/10495142.2020.1865234.

Hou Y, Poliquin CW. 2022. The Effects of CEO Activism: Partisan Consumer Behavior and Its Duration. Strateg Manag J. 0(0):1–32. doi:10.1002/smj.3451.

Hydock C, Paharia N, Blair S. 2020. Should Your Brand Pick a Side? How Market Share Determines the Impact of Corporate Political Advocacy. J Mark Res. 57(6):1135–1151. doi:10.1177/0022243720947682.

Jin J, Mitson R, Qin YS, Vielledent M, Men LR. 2022. Can CEO Activism Be Good for the Organization? The Importance of Authenticity, Morality, and Timeliness. Journal Mass Commun Q. 00(0). doi:10.1177/10776990221116377.

Jin J, Mitson R, Qin YS, Vielledent M, Men LR. 2023. Enhancing young consumer’s relational and behavioral outcomes: The impact of CEO activism authenticity and value alignment. Public Relat Rev. 49(2):102312. doi:10.1016/j.pubrev.2023.102312.

Jungblut M, Johnen M. 2022. When Brands (Don’t) Take My Stance: The Ambiguous Effectiveness of Political Brand Communication. Communic Res. 49(8):1092–1117. doi:10.1177/00936502211001622.

Karikari S. 2023. Riding The Brand Activism Wave: Three Essays On Responses To Incongruence In Brand Activism. [Doctoral dissertation, Morgan State Universty]. https://www.proquest.com/docview/2814281241?pq-origsite=gscholar&fromopenview=true.

Lee E. 2023. Perceived Authenticity in Organizational Advocacy. [Doctoral dissertation, Indiana University]. https://www.proquest.com/docview/2811849913?pq-origsite=gscholar&fromopenview=true.

Lee SY, Chung S. 2023. Publics’ Views of Corporate Social Advocacy Initiatives: Exploring Prior Issue Stance, Attitude Toward a Company, and News Credibility. Manag Commun Q. 37(2):281–309. doi:10.1177/08933189221105808.

Lee Y, Tao W. 2021. Does Perceived Morality of CEO Activism Matter? Understanding Employees’ Responses to CEO Actions on Sociopolitical Issues. Manag Decis. 59(10):2329–2354. doi:10.1108/MD-04-2020-0498.

Lee Z, Spry A, Ekinci Y, Vredenburg J. 2023. From warmth to warrior: impacts of non-profit brand activism on brand bravery, brand hypocrisy and brand equity. J Brand Manag.(0123456789). doi:10.1057/s41262-023-00319-8.

Li J, Kim JK, Alharbi K. 2021. Exploring the Role of Issue Involvement and Brand Attachment in Shaping Consumer Response Toward Corporate Social Advocacy (CSA) Initiatives: The Case of Nike’s Colin Kaepernick Campaign. Int J Advert. 41(2):233–257. doi:10.1080/02650487.2020.1857111.

Liaukonytė J, Tuchman A, Zhu X. 2023. Rejoinder: Spilling More Beans on Political Consumerism: It’s More of the Same Tune. Mark Sci. 42(1):32–36. doi:10.1287/mksc.2022.1411.

Lim HS, Moon WK, Ciszek E. 2023. Advertising for Brands and Society: The Role of Perceived Authenticity in Corporate Transgender Advocacy Advertising Campaigns. J Homosex. 00(00):1–29. doi:10.1080/00918369.2023.2245522.

Lim JS, Young C. 2021. Effects of Issue Ownership , Perceived Fit , and Authenticity in Corporate Social Advocacy on Corporate Reputation. Public Relat Rev. 47(4):102071. doi:10.1016/j.pubrev.2021.102071.

Mello NSB. 2022. The Effects of Corporate Sociopolitical Advocacy on Consumer Cause-Related Engagement. [Master dissertation, Universidade Federal do Rio Grande do Sul]. https://www.lume.ufrgs.br/handle/10183/248006.

Mesquita E, Lopes EL, Herrero E. 2022. “What if a Person Gets the Vaccine and Turns into an Alligator?”: A Study of the Effect of Ideological Polarization on Purchase Intention. Brazilian Bus Rev. 19(5):565–583. doi:10.15728/bbr.2022.19.5.6.en.

Meyer L. 2022. The Impact of Corporate Brand Activism and Brand Attachment on Consumer Responses in the Sportwear Industry. master dissertation, Department of Communication Science, University of Twente, The Netherlands.

Mukherjee S, Althuizen N. 2020. Brand Activism: Does Courting Controversy Help or Hurt a Brand? Int J Res Mark. 37(4):772–788. doi:10.1016/j.ijresmar.2020.02.008.

Nam J, Balakrishnan M, De Freitas J, Brooks AW. 2023. Speedy activists: How firm response time to sociopolitical events influences consumer behavior. J Consum Psychol. 33(4):632–644. doi:10.1002/jcpy.1380.

Naouri A. 2022. The Power of Controversy – The Allure of Controversial Brands. Nova School of Business and Economics.

Nguyen DT, Le DHA, Truong LG, Truong NG, Vu VV. 2022. The effect of Generation Z’s Perceptions of Brand Activism on Brand Loyalty: Evidence from Vietnam. Asia Pacific J Mark Logist. 0(0):1–19. doi:10.1108/APJML-02-2022-0165.

Overton H, Choi M, Weatherred JL, Zhang N. 2020. Testing the Viability of Emotions and Issue Involvement as Predictors of CSA Response Behaviors. J Appl Commun Res. 48(6):695–713. doi:10.1080/00909882.2020.1824074.

Overton H, Kim JK, Zhang N, Huang S. 2021. Examining Consumer Attitudes Toward CSR and CSA Messages. Public Relat Rev. 47(4):102095. doi:10.1016/j.pubrev.2021.102095.

Panizzo R. 2021. Effectiveness of Corporate Sociopolitical Activism on brand related outcomes: an Italian perspective. [Master disseration, Università Ca’Foscari Venezia]. http://dspace.unive.it/handle/10579/21094.

Park K. 2022. The mediating role of skepticism: how corporate social advocacy builds quality relationships with publics. J Mark Commun. 28(8):821–839. doi:10.1080/13527266.2021.1964580.

Park K, Jiang H. 2020. Signaling, Verification, and Identification: The Way Corporate Social Advocacy Generates Brand Loyalty on Social Media. Int J Bus Commun. 0(0):1–25. doi:10.1177/2329488420907121.

Reichert I, Drzimalla M, Böthig A. 2022. Is Brand Activism Boosting Employer Brand Attractiveness? In: Special Issue Innovative Brand Management III. p. 39–57.

Rim H, Xu H, Dong C. 2022. Triadic Public-Company-Issue Relationships and Publics’ Reactions to Corporate Social Advocacy (CSA): An Application of Balance Theory. J Public Relations Res. 34(3–4):109–134. doi:10.1080/1062726X.2022.2071273.

Sauter S, Jungblut M. 2023. It’s Good for Our Reputation (?!)–the Impact of Socio-Political CEO Communication on Corporate Reputation. Int J Strateg Commun. 00(00):1–19. doi:10.1080/1553118X.2023.2236090.

Schleier M. 2021. Standing Up for Societal Change: The Phenomenon of Brand Activism. A Quantitative Investigation on Young Consumers’ Attitude Towards an Environmental Activist Brand. [Master dissertation, Lund University]. https://lup.lub.lu.se/luur/download?func=downloadFile&recordOId=9051281&fileOId=9051285.

Song B, Choi M. 2023. Testing Publics’ Perceptions of Corporate Social Advocacy Messaging: Linking Organizational and Social Outcomes. Int J Strateg Commun. 00(00):1–20. doi:10.1080/1553118X.2023.2228293.

Song B, Lan X. 2022. Meeting Consumers’ Expectations: Exploring Corporate Social Advocacy Communication in China. Sustain. 14(4):2385. doi:10.3390/su14042385.

Tan CSL. 2023. The times they are a-changing: examining the effects of luxury brand activism on political consumerism and eWOM. J Strateg Mark. 00(00):1–20. doi:10.1080/0965254X.2023.2232791.

Troy CLC, Norman MLP, Eng N, Freeman J, Bortree DS. 2023. Effects of climate CSA and CSR messaging: the moderating role of green consumer identity. Corp Commun. doi:10.1108/CCIJ-02-2023-0019.

Tsai JY, Yuan S, Coman IA. 2023. Theorizing issue-driven public attention and expectations in audience responses to corporate sociopolitical activism: A mixed-method analysis. Public Relat Rev. 49(4):102353. doi:10.1016/j.pubrev.2023.102353.

Valerie I, Hariandja ES. 2022. Influence of Values, Brand Activism, Brand Consciousness, and Behavioral Intentions in Predicting Nike’s Sportswear Consumption in Indonesia. In: Proceeding of the 5th National Conference Business, Management, and Accounting on The Opportunity of Digital and Technology Disruption. Tangerang: Universitas Pelita Harapan, Indonesia. p. 535–558.

Vasquez R. 2022. CSR, CSA, or CPA? Examining Corporate Climate Change Communication Strategies, Motives, and Effects on Consumer Outcomes. Sustainability. 14(6):3604. doi:10.3390/su14063604.

Villagra N, Clemente-Mediavilla J, López-Aza C, Sánchezherrera J. 2021. When Polarization Hits Corporations: The Moderating Effect of Political Ideology on Corporate Activism. Prof la Inf. 30(6):1–20. doi:10.3145/epi.2021.nov.02.

Wang CC, Chang SC, Chen PY. 2021. The brand sustainability obstacle: Viewpoint incompatibility and consumer boycott. Sustain. 13(9):1–23. doi:10.3390/su13095174.

Wang Y, Bouroncle L. 2023. To Trust or Not to Trust: Consumer Perceptions of Corporate Sociopolitical Activism. Bus Prof Commun Q. doi:10.1177/23294906231166141.

Wannow S, Haupt M, Ohlwein M. 2023. Is brand activism an emotional affair? The role of moral emotions in consumer responses to brand activism. J Brand Manag. 31(0123456789):168–192. doi:10.1057/s41262-023-00326-9.

Weber TJ, Joireman J, Sprott DE, Hydock C. 2023. Differential Response to Corporate Political Advocacy and Corporate Social Responsibility: Implications for Political Polarization and Radicalization. J Public Policy Mark. 42(1):74–93. doi:10.1177/07439156221133073.

Xie T. 2022. A Theory of Corporate Social Advocacy Legitimacy: Perspective Change, Scale Development, and Model Testing. [Doctoral dissertation, University of Georgia]. https://www.proquest.com/docview/2685418497?pq-origsite=gscholar&fromopenview=true.

Xu H, Lee E, Rim H. 2021. Should businesses take a stand? Effects of perceived psychological distance on consumers’ expectation and evaluation of corporate social advocacy. J Mark Commun. 00(00):1–24. doi:10.1080/13527266.2021.1969588.

You L, Wang R, Lan X, Hon L. 2023. Exploring predictors of consumer digital engagement and political consumerism in corporate political advocacy. Telemat Informatics. 79(February):101955. doi:10.1016/j.tele.2023.101955.

Young C. 2017. The Impact of Ben & Jerry’s Corporate Social Advocacy on Corporate Reputation and Brand Loyalty. [Master dissertation, Syracuse University]. https://www.proquest.com/docview/2430682965?pq-origsite=gscholar&fromopenview=true.

Zhang X, Zhou Z. 2023. The influence of issue attitude, value involvement and consumer-company identification on consumers’ reactions to corporate social advocacy: a moderated mediation through cognitive dissonance. J Commun Manag. doi:10.1108/JCOM-05-2022-0053.

Zhang XA, Borden J. 2022. How legitimate are corporate social advocacy campaigns? An examination of the role of legitimacy in stakeholder perceptions of CSA. J Mark Commun. 00(00):1–25. doi: 10.1080/13527266.2022.2105931.

Zhou X, Lou C, Huang X. 2023. Transcendent Brand Activism Advertising: Explicating the Roles of Color and Message Framing in Advertising Effectiveness. J Advert. 0(0):1–17. doi:10.1080/00913367.2023.2217866.

Zhou Z, Dong C. 2022. Matching Words with Actions: Understanding the Effects of CSA Stance-Action Consistency on Negative Consumer Responses. Corp Commun An Int J. 27(1):167–187. doi:10.1108/CCIJ-05-2021-0060.
